# Low versus high dose erythropoiesis-stimulating agents in hemodialysis patients with anemia: A randomized clinical trial

**DOI:** 10.1371/journal.pone.0172735

**Published:** 2017-03-01

**Authors:** Valeria Saglimbene, Suetonia C. Palmer, Jonathan C. Craig, Marinella Ruospo, Antonio Nicolucci, Marcello Tonelli, David Johnson, Giuseppe Lucisano, Gabrielle Williams, Miriam Valentini, Daniela D’Alonzo, Fabio Pellegrini, Paolo Strippoli, Mario Salomone, Antonio Santoro, Stefano Maffei, Jörgen Hegbrant, Gianni Tognoni, Giovanni F. M. Strippoli, for the CE-DOSE Study Investigators

**Affiliations:** 1 Sydney School of Public Health, Edward Ford Building, The University of Sydney, New South Wales, Australia; 2 Diaverum Renal Services Group, Lund, Sweden; 3 Department of Medicine, University of Otago Christchurch, Christchurch, New Zealand; 4 Amedeo Avogadro University of Eastern Piedmont, Novara, Italy; 5 Center for Outcomes Research and clinical Epidemiology (CORESEARCH), Pescara, Italy; 6 Cumming School of Medicine, Health Sciences Centre, University of Calgary, Foothills Campus, Calgary, Alberta, Canada; 7 Department of Renal Medicine, Division of Medicine, University of Queensland at the Princess Alexandra Hospital, Woolloongabba, Queensland, Australia; 8 Translational Research Institute, University of Queensland, Woolloongabba, Queensland, Australia; 9 Quintiles srl, Milan, Italy; 10 ASL, Lanciano Vasto, Chieti, Italy; 11 Global Medical Biogen Idec, Cambridge, Massachusetts, United States of America; 12 Department of Nephrology and Dialysis, Ospedale " A. Perrino", Brindisi, Italy; 13 Department of Nephrology and Dialysis, Ospedale Maggiore di Chieri, Chieri, Italy; 14 Department of Nephrology and Dialysis, Policlinico S. Orsola-Malpighi, Bologna, Italy; 15 Mario Negri Institute for Pharmacological Research, Milano, Italy; 16 Department of Emergency and Organ Transplantation, University of Bari, Bari, Italy; Cardiff University, UNITED KINGDOM

## Abstract

The increased risks of death and adverse events with erythropoiesis-stimulating agent (ESA) therapy targeting a higher hemoglobin level are established. It is uncertain whether the adverse effects of ESA therapy are related to dose and are mitigated when a fixed low ESA dose is used. We conducted a multicenter, prospective randomized open-label, blinded-endpoint (PROBE) trial to evaluate fixed low versus high dose ESA therapy on patient outcomes. We intended to recruit 2104 hemodialysis patients >18 years with anemia or receiving ESA treated at dialysis clinics in Italy. The intervention was fixed low (4000 IU epoetin alfa equivalent weekly) or high (18,000 IU epoetin alfa equivalent weekly) dose ESA for 12 months. Primary outcomes were serum transferrin, ferritin, albumin, C-reactive protein and ESA dose. Secondary outcomes were the composite of death or cardiovascular event, all-cause mortality, cardiovascular mortality, myocardial infarction, stroke, cardiovascular hospitalization, and quality of life. Study recruitment was terminated after inclusion of 656 participants with convergence of ESA dose between groups during follow up. Fixed low dose ESA had uncertain effects on serum ferritin (delta of delta (DD) 3.9 ng/ml, 95% CI -85.0 to 92.8), transferrin (9.2 mg/dl, -6.3 to 24.8), transferrin saturation (3.7%, -5.0 to 12.3), serum albumin (-0.03 g/dl, -0.2 to 0.1), or C-reactive protein (-0.6 mg/l, -3.3 to 2.1). In addition, fixed dose therapy had inconclusive effects on the composite endpoint of mortality and cardiovascular events (hazard ratio [HR] 0.95, 95% CI 0.66 to 1.37), death (0.98, 0.64 to 1.52), nonfatal myocardial infarction (0.52, 0.18 to 1.52), nonfatal stroke (no events), hospital admission for cardiovascular causes (0.93, 0.50 to 1.72) or health-related quality of life. A fixed low ESA dose in hemodialysis patients has uncertain effects on serum parameters, mortality, cardiovascular events, and quality of life. Hemoglobin targets may be so entrenched in nephrology practice that a trial of ESA dose is no longer possible.

## Introduction

Erythropoiesis—stimulating agents (ESA) revolutionized anemia management in chronic kidney disease (CKD) after early proof-of-concept studies and randomized trials showed that ESAs increased hemoglobin levels and reduced the need for blood transfusions.[1–4] Observational studies found that lower hemoglobin levels were associated with cardiovascular and mortality outcomes,[5, 6] which prompted widespread anemia correction with ESAs.[7] As the focus of randomized trials changed from anemia management measured by hemoglobin levels to prevention of cardiovascular endpoints, larger randomized trials compared different hemoglobin targets on mortality and vascular events.[8, 9] Unexpectedly, these studies showed that complete anemia correction increased the risks of death[10] and major cardiovascular events.[11] This evidence led to lower recommended hemoglobin levels and re-evaluation of the relative roles of ESA dose and targeted hemoglobin levels on adverse patient outcomes.[12]

Secondary analyses of hemoglobin target trials and observational studies suggested that higher ESA doses needed to achieve anemia correction were associated with higher risks of all-cause mortality and cardiovascular events,[13–15] raising the possibility that the association between a higher hemoglobin target and adverse patient outcomes was mediated through ESA dose and the need for a trial to test the hypothesis. However, the titration of ESA to hemoglobin target has been so universal in both research and clinical practice that no major trial has evaluated whether a fixed low treatment dose can correct anemia while mitigating adverse effects of treatment on mortality or cardiovascular outcomes and maintain quality of life.[11]

We conducted the Clinical Evaluation of the DOSE of erythropoietin (C.E. DOSE) trial to test the hypothesis that, in patients with anemia and end-stage kidney disease treated with dialysis, fixed low dose ESA would lower rates of mortality and cardiovascular complications but maintain health-related quality of life compared with a fixed high dose strategy.[16]

## Methods

This study was a multicenter, Prospective Randomized Open—label, Blinded-Endpoint (PROBE)[17], parallel group, controlled trial compared fixed low dose with fixed high dose ESA treatment. The protocol has been published and is provided as S1 File (English) and S2 File (Italian).[16] The study was designed in 2005 and received funding in 2006.[18] The study was approved by ethics committees (S1 Appendix) and coordinated by the Fondazione Mario Negri Sud with funding from the Italian Medicines Agency (Agenzia Italiana del Farmaco [AIFA]: grant number FARM6X822T) (S2 Appendix). The trial was registered at ClinicalTrials.gov, number NCT00827021 (https://clinicaltrials.gov/ct2/show/NCT00827021). The trial has been reported according to the 2010 CONSORT checklist of information to include when reporting a randomized trial (S3 Appendix). All participants gave written informed consent before involvement in the study.

### Study population

The 41 study sites included 38 public hospitals and three private outpatient hemodialysis clinics in Italy. Eligible patients were adults aged ≥18 years, long—term outpatient based hemodialysis, anemia without ESA treatment (hemoglobin below 10 g/dl) or were already receiving treatment with an ESA (and had a hemoglobin level below 13 g/dl). Patients who had a known contraindication to ESA treatment were ineligible. Participant recruitment occurred between 13 July 2009 and 19 July 2013.

### Intervention

Participants were randomized to fixed low dose ESA (epoetin alfa or beta 4,000 IU or darbepoetin alfa 20 mcg weekly) or fixed high dose ESA (epoetin alfa or beta 18,000 IU or darbepoetin alfa 90 mcg weekly) without a washout period. Although this was a fixed dose trial, a hemoglobin value outside the pre—specified safety range of 9.5–12.5 g/dl prompted the treating physician to increase or decrease the study drug dose by 25% by protocol. After dose adjustment, a fixed treatment dose was again maintained. The dosing algorithm for ESA used by clinicians is described the S4 Appendix.

### Randomization and blinding

Patients were randomly assigned to low dose or high dose ESA with randomization stratified by dialysis clinic and in randomly permuted blocks of six. The random sequence for the allocation program was created in FileMaker Pro 10 and allocation was concealed to researchers by using remote, central assignment of treatment via telephone contact with masked researchers at the central trial coordination unit. Participants and investigators were not masked to group allocation, but assessors collecting and evaluating data for treatment outcomes were unaware of treatment assignment. Participants received non—randomized co—interventions according to the usual clinical practice within the study center to achieve and maintain clinical quality performance measures (S5 Appendix).

### Follow-up

Routine data were collected by study researchers at the clinical sites using a paper-based case report form derived from routinely-collected dialysis care records, laboratory systems, or from information provided by the participants to their dialysis physician. Measurements of hemoglobin levels and vital signs were performed at visits at 0.5, 1, 1.5, 2, 2.5, 3, 6, and 12 months after randomization. Other laboratory assessments were performed at baseline, and 6 and 12 months. Health-related quality of life was self—reported by participants using the Kidney Disease Quality of Life Short Form (KDQOL-SF^™^) version 1.3 at 0, 6, and 12 months. Information about deaths was collected from death certificates generated by regional health authorities. Adverse events were assigned as serious adverse events by the treating clinician at the investigation site and field—based researchers who were unaware of treatment allocation assigned the adverse events to specific Medical Dictionary for Regulatory Activities (MedDRA) categories.

### Outcomes

Primary outcomes were serum transferrin, ferritin, albumin, C-reactive protein and ESA dose. Secondary outcomes were the composite of death or cardiovascular event, all-cause mortality, cardiovascular death, dialysis vascular access thrombosis, hypertension, seizures, blood transfusion, or health-related quality of life domains at months 6 and 12. Follow up was completed on 19 July 2014. Definitions of study endpoints are reported in S6 Appendix. An independent end-point adjudication committee including two cardiologists and two nephrologists who were unaware of treatment allocation reviewed all available endpoint documentation (including treatment charts, death certificates and medical records) to provide masked adjudication of mortality and cardiovascular outcome events.

### Sample size

We aimed to recruit 2104 adults to detect a risk reduction in the composite end-point of mortality and major cardiovascular event with the experimental intervention (low fixed ESA dose) of 15% (hazard ratio = 0.85) at 4 years with an expected annual incidence of the composite endpoint of 15% based on data from existing trials.[9, 19] We determined that this number of participants would provide the study with a power of 80%, with a two—sided type 1 error of 5% and allowing for a non-adherence rate of 5%. An on-treatment risk of 15% per annum was based on existing randomized trials evaluating erythropoiesis stimulating agents in adults with end-stage kidney disease.[9]

During the trial recruitment period (2009–2014), clinical guidelines were published in 2012 that suggested a more conservative approach to ESA treatment than previously, by starting ESA therapy when then hemoglobin was between 9.0–10.0 g/dl.[12] A warning was issued by the US Food and Drug Administration in 2011 that ESA treatment should not be used to maintain hemoglobin concentrations above 11.0 g/dl.[20] As a result of these new recommendations, slow recruitment of the trial occurred. The trial sponsor changed the primary outcome from the composite of death or major cardiovascular event to biochemical markers of ESA responsiveness including the individual endpoints of ferritin, transferrin, transferrin saturation, serum albumin, and C-reactive protein and mean ESA dose at end of treatment. The sample size was recalculated by the sponsor to include 900 patients followed for 12 months to detect a “small effect size” (standardized mean difference of 0.2, with an alpha = 0.05 and 1-beta = 0.80) of fixed dose treatment on these individual endpoints. The trial recruitment was stopped early at 656 participants by the trial steering committee due to slow recruitment and convergence of ESA dose in the two treatment groups.

### Statistical analysis

Summaries of continuous variables were calculated as means (SD) for normally distributed data and as medians with interquartile ranges for skewed data; categorical variables are presented as frequencies (proportions). Survival curves were generated according to the Kaplan—Meier method and were expressed as a cumulative incidence. We compared the two treatment groups using Cox proportional hazards model to estimate the hazard ratio (HR) and 95% CI. For the analysis of quality of life, blood pressure and individual biochemical endpoints, we used multilevel regression models with an unstructured correlation-type matrix to account for repeated data measures, and expressed the difference between groups as a delta of delta (DD; the difference between dose (low versus high) over time (12 months versus baseline) together with the corresponding 95% CI.[21] The risk of serious and any adverse event during the follow-up was estimated through a Poisson regression analysis. Incidence rates (IR) for adverse events were expressed as the number of events per 1000 participant-years of follow up and the risk between groups was expressed as an incidence rate ratio (IRR). All patients were followed until death or the end of the trial, with censoring of data at the time a patient underwent kidney transplantation or was lost to follow up. For the composite outcome, a subgroup analysis by age, gender, diabetes, and presence of cardiovascular disease was performed. All analyses were conducted according to the intention-to-treat principle without imputation.

To determine whether the treatment effect varied according to the clinical response to ESA treatment status, *a priori* sensitivity analyses were planned with inclusion of only patients who showed ESA resistance. We used a modified definition described in the Handling Erythropoietin Resistance with Oxpentifylline (HERO) Trial, in which we considered patients to have ESA resistance if they had a hemoglobin level ≤12 g/deciliter on average during the 3 months before randomization and a weekly ESA dose at randomization ≥200 IU/kg body weight/week for epoetin alfa or beta or ≥ 1 μg/kg body weight/week for darbepoetin at randomization.[22] All statistical tests were two—tailed. Analyses were performed using SAS Software Release 9.4 (SAS Institute, Cary, NC).

## Results

### Recruitment and baseline characteristics

**Fig 1:**
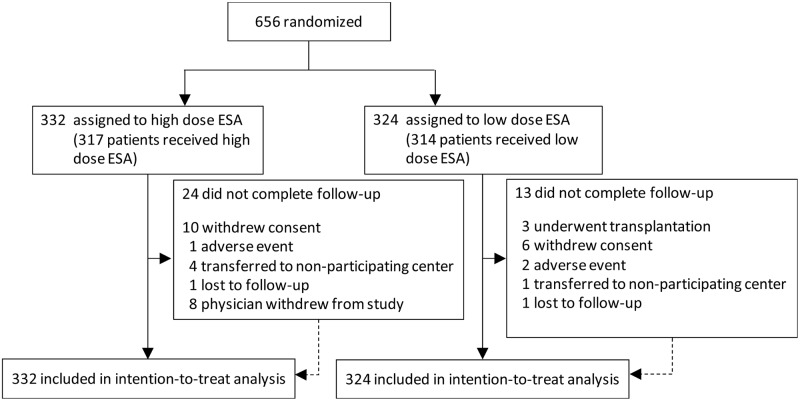
CONSORT flow diagram of patient participation and follow up.

656 patients were recruited (Fig 1). After randomization, 25 patients did not receive allocated treatment (10 in the low dose group and 15 in the high dose group). Of the ten patients randomized to low dose and who did not receive allocated treatment, one was not prescribed treatment, eight received the incorrect dose, and one was not treated based on a physician’s decision. Of the 15 patients randomized to high dose therapy who did not receive allocated treatment, one patient withdrew consent, seven did not meet inclusion criteria, and seven did not receive the correct treatment dose. The study included a median follow-up duration of 364 days (95% CI 21–365 days). At that time, 619 patients (94.4%) had completed a 12-month follow-up visit or had died. This included 311 (96.0%) in the low dose and 308 (92.8%) in the high dose group. Baseline characteristics were well-balanced between the treatment groups except for a history of dyslipidemia and statin use which were more frequent in the high dose group (Table 1). The raw study data are provided in Tables A-D in S3 File.

**Table 1:** Baseline characteristics.

Variable	Low dose group (n = 324)	High dose group (n = 332)
Age (years)	65.2 ± 15.2	66.6 ± 12.9
Sex		
Female	117 (36.1)	135 (40.7)
Male	207 (63.9)	197 (59.3)
Ethnic origin		
White	304 (93.8)	314 (94.6)
Black	2 (0.6)	2 (0.6)
Other	2 (0.6)	2 (0.6)
Primary cause of end-stage kidney disease		
Primary glomerulonephritis	54 (16.7)	58 (16.7)
Hypertension/diabetes/vascular disease	133 (41.0)	147 (44.3)
Congenital including cystic disease	25 (7.7)	23 (6.9)
Interstitial nephritis	5 (1.5)	6 (1.8)
Pyelonephritis	16 (4.9)	19 (5.7)
Hereditary disorder	5 (1.5)	5 (1.5)
Other	17 (5.2)	10 (3.0)
Coexisting conditions		
Hypertension	237 (73.1)	244 (73.5)
Dyslipidemia	78 (24.1)	109 (32.8)
Diabetes	70 (21.1)	90 (27.1)
Chronic lung disease	45 (13.9)	41 (12.3)
Ischemic heart disease	60 (18.5)	80 (24.1)
Transient ischemic attack	13 (4.0)	16 (4.8)
Heart failure	27 (8.3)	25 (7.5)
Cardiac arrhythmia	45 (13.9)	41 (12.3)
Seizures	3 (0.9)	4 (1.2)
Thromboembolic event	14 (4.3)	19 (5.7)
Wait-listing for kidney transplantation	39 (12.0)	30 (9.0)
Previous kidney transplantation	35 (10.5)	36 (10.8)
Smoking status		
Current	31 (9.6)	38 (11.4)
Former	81 (25.0)	79 (23.8)
Never smoked	195 (60.2)	199 (59.9)
Medications		
Antihypertensive		
Diuretic	29 (9.0)	29 (8.7)
Beta blocker	83 (25.6)	84 (25.3)
Calcium channel blocker	96 (29.6)	83 (25.0)
Angiotensin converting enzyme inhibitor	50 (15.4)	39 (11.8)
Angiotensin II receptor blocker	45 (13.9)	36 (10.8)
Aspirin	129 (39.8)	126 (38.0)
Vitamin D compound	190 (58.6)	196 (59.0)
Phosphate-binding agent	250 (77.2)	251 (75.6)
Calcimimetic agent	57 (17.6)	58 (17.5)
Statin	77 (23.8)	109 (32.8)
Iron therapy	184 (56.8)	191 (57.5)
Erythropoiesis-stimulating agent	303 (93.5)	313 (94.3)
Weekly erythropoietin-stimulating agent dose		
Epoetin alfa or beta (international units)	8000 (5000–15,000)	9000 (6000–16,000)
Darbepoetin alfa (μg)	40 (25–60)	30 (20–60)
Clinical characteristics		
Weight (kg)	68.3 (14.3)	69.3 (16.5)
Blood pressure (mm Hg)		
Systolic	134.9 (25.2)	134.2 (22.6)
Diastolic	71.1 (14.0)	70.5 (13.9)
Results of blood tests		
Hemoglobin (g per liter)	11.0 (1.0)	11.0 (1.0)
Hematocrit (%)	34.4 (3.8)	34.4 (3.7)
Albumin (g per deciliter)	3.71 (0.44)	3.70 (0.47)
Phosphorus (mg per deciliter)	4.66 (1.68)	4.80 (1.56)
Serum transferrin (mg per deciliter)	189 (159–220)	187 (154–214)
Transferrin saturation (%)	23.8 (17.0–32.7)	23.0 (17.0–31.9)
Ferritin (ng per milliliter)	322 (408–522)	300 (376–477)
Intact parathyroid hormone** (picograms per milliliter)	211 (71–339)	187 (78–334)
C-reactive protein (mg per liter)	0.9 (0.3–4.1)	0.9 (0.4–4.0)
Dialysis characteristics		
Time treated with dialysis (months)	47 (15–80)	51 (11–83)
Duration per dialysis treatment (minutes)	232 (23.5)	232 (21.0)
Blood flow (milliliters/minute)	298 (33.5)	299 (34.3)
Kt/V urea	1.40 (0.35)	1.43 (0.38)

Data are mean (SD) or median (IQR), or frequency (percentage).

**Data were collected as pmol/l and converted to pg/ml

### Hemoglobin levels

**Fig 2:**
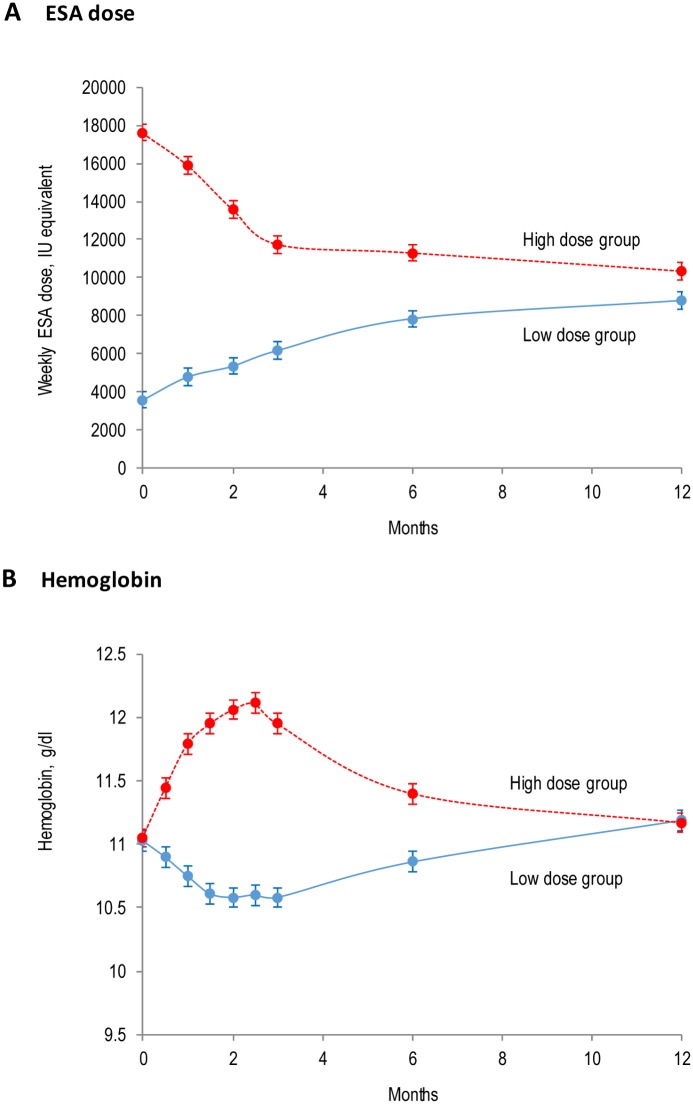
Erythropoietin-stimulating agent doses and hemoglobin levels during follow up. Data are shown as mean and the standard error of mean. Darbepoetin alfa doses have been converted to equivalent dose of epoetin alfa or beta. ESA, erythropoiesis-stimulating agent.

The hemoglobin levels were similar in the low dose and high dose groups at baseline (Table 1) and diverged significantly reaching maximal separation at between 2 and 3 months, before complete convergence of hemoglobin levels at 12 months, in parallel with loss of ESA dose separation (mean hemoglobin at 12 months 11.2 ± 0.7 g/dl in low dose; p = 0.73 versus 11.2 ± 0.8 g/dl in high dose group) (Fig 2).

### Outcomes

#### Primary outcomes

There was initial separation of ESA dose between treatment groups at randomization, with rapid subsequent convergence of ESA dose (Fig 2). ESA dose remained significantly different in the low dose and high dose groups at 12 months (8770 IU, 95% CI 7863–9676 IU in low dose group versus 10,337 IU, 95% CI 9432–11,242 IU in high dose group; p = 0.01). At the end of follow-up, 147 participants were receiving allocated fixed dose ESA (n = 61 in high dose group and n = 86 in low dose group).

The median time from randomization to the first ESA dose adjustment was 61 days (IQR 32–110) in the low dose and 54 days (IQR 31–92) in the high dose group (p = 0.17) (S1 Table). The median time from randomization to stabilization of hemoglobin levels was 34 days (IQR 28–87) in the low dose and 34 days (IQR 28–92) in the high dose group (p = 0.41). The total number of dose changes in the same time period was 28.0 per 100 patient-months (95% CI 22.9–34.3) with low dose (p = 0.89) and 28.5 per 100 patient-months (95% CI 24.3–33.4) with high dose ESA.

There was no difference at end of treatment between low dose and high dose groups for serum ferritin, serum transferrin, or transferrin saturation, albumin, or C-reactive protein levels (Table 2).

**Table 2:** Primary endpoints.

Hematinic	Baseline		12 months		Difference between high dose and low dose over time (12 months versus baseline; 95% CI) ^b^	p value
	Low dose ^a^	High dose ^a^	Low dose ^a^	High dose ^a^		
Serum ferritin, ng/ml	464.5 (28.0)	426.9 (27.6)	474.9 (31.6)	441.2 (32.2)	3.9 (-85.0 to 92.8)	0.93
Serum transferrin, mg/dl	189.9 (4.0)	187.5 (3.9)	186.4 (4.7)	193.2 (4.8)	9.2 (-6.3 to 24.8)	0.24
Transferrin saturation, %	29.4 (2.2)	29.6 (2.2)	28.1 (2.4)	32.0 (2.4)	3.7 (-5.0 to 12.3)	0.41
Serum albumin, g/dl	3.8 (0.8)	3.7 (0.6)	3.8 (0.5)	3.7 (0.6)	-0.03 (-0.2 to 0.1)	0.65
C-reactive protein, mg/l	5.3 (13.2)	3.8 (8.0)	6.1 (16.0)	5.2 (15.3)	-0.6 (-3.3 to 2.1)	0.69

^a^ Data are unadjusted means (and standard error)

^b^ Data are unadjusted means (difference between low dose and high dose treatment from baseline to 12 months; delta of delta) and 95% confidence interval

The p value indicates the difference between low dose and dose treatment over time (delta of delta [DD]).

#### Secondary outcomes

**Fig 3:**
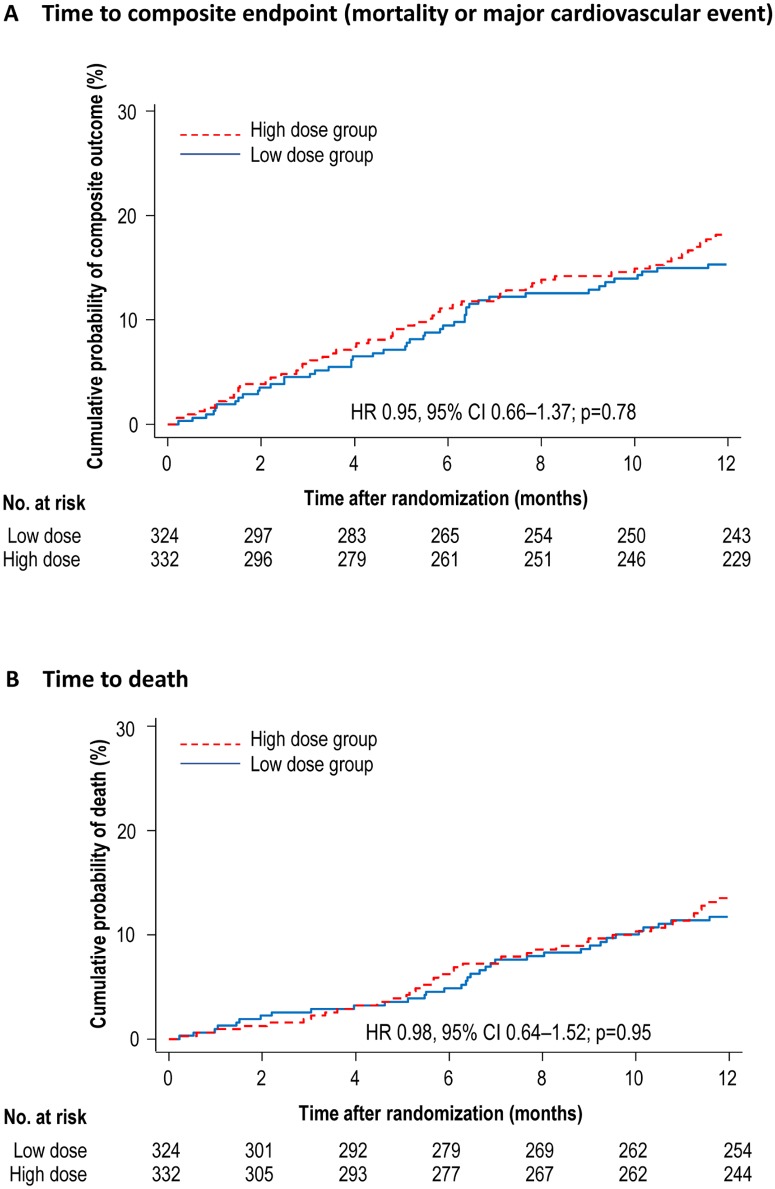
Effects of low dose versus high dose ESA on composite endpoint of mortality and major cardiovascular event) and all-cause mortality at 12 months. HR = hazard ratio.

Fixed dose ESA had uncertain effects on the composite of outcome of death from any cause, nonfatal myocardial infarction, nonfatal stroke, or hospitalization for cardiovascular cause (HR 0.95, 95% CI 0.66–1.37, p = 0.78; Table 3 and Fig 3) and individual endpoints including death (HR 0.98, 95% CI 0.64–1.52, p = 0.95), fatal myocardial infarction (HR 0.69, 95% CI 0.19–2.44, p = 0.57), nonfatal myocardial infarction (HR 0.52, 95% CI 0.18–1.52, p = 0.23), hospital admission from cardiovascular causes (HR 0.93, 95% CI 0.50–1.72, p = 0.80), dialysis vascular access thrombosis (HR 0.80, 95% CI 0.41–1.54, p = 0.50), or hypertension (HR 1.85, 95% CI 0.62–5.56, p = 0.28) (Fig 3). Risk estimates for fatal stroke, nonfatal stroke, and hospital admission for seizures were not available due to zero events in one or both treatment groups.

**Table 3:** Secondary endpoints.

Outcome	Low dose group (n = 324)	High dose group (n = 332)	Hazard ratio (95% CI)	p value
Composite of death from any cause, nonfatal myocardial infarction, nonfatal stroke, or hospitalization for cardiovascular cause*	54 (17%)	60 (18%)	0.95 (0.66–1.37)	0.78
Death from any cause	40 (12%)	43 (13%)	0.98 (0.64–1.52)	0.95
Fatal myocardial infarction	4 (1%)	6 (2%)	0.69 (0.19–2.33)	0.57
Nonfatal myocardial infarction	5 (2%)	10 (3%)	0.52 (0.18–1.52)	0.23
Fatal stroke	2 (1%)	0 (0%)	Not estimable	--
Nonfatal stroke	0	0	Not estimable	--
Hospital admission from cardiovascular causes	19 (6%)	21 (6%)	0.93 (0.50–1.72)	0.80
Dialysis vascular access thrombosis	16 (5%)	20 (6%)	0.80 (0.41–1.54)	0.50
Hospital admission for seizures	0	0	Not estimable	--
Hypertension	9 (3%)	5 (2%)	1.85 (0.62–5.56)	0.28
Red blood cell transfusion	30 (9%)	15 (5%)	2.44 (1.23–4.76)	0.007

Data were expressed as number and percent.

* Participants may have had multiple cardiovascular events during follow-up. The composite endpoint reflects only the first occurrence of any of the individual components of the endpoint.

Overall, 126 serious adverse events occurred in patients allocated to low dose ESA whereas 146 serious adverse events were experienced by patients allocated to high dose ESA (incidence rate ratio 0.88, 95% CI 0.69–1.12, p = 0.31) (Table 4). There were 227 adverse events in the low dose group and 232 in the high dose group (incidence rate ratio 1.00, 95% CI 0.83–1.20, p = 0.99). The most frequent serious adverse events related to infections and infestations, cardiac disorders, and vascular disorders (S2 and S3 Tables).

**Table 4:** Adverse events and serious adverse events.

Outcome	Low dose group (N = 324)	High dose group (N = 332)
**Adverse events**		
Any adverse event	227 (58)	232 (58)
Most common (≥2 per 1000 patient-months of follow-up) in high dose arm		
Infections and infestations	53 (14)	47 (12)
Cardiac disorders	22 (6)	21 (5)
Vascular disorders	24 (6)	14 (4)
Gastrointestinal disorders	22 (6)	11 (3)
Injury, poisoning and procedural complications	18 (5)	13 (3)
Respiratory, thoracic and mediastinal disorders	10 (3)	13 (3)
Nervous system disorders	13 (3)	8 (2)
Neoplasms (benign, malignant, unspecified)	9 (2)	9 (2)
Surgical and medical procedures	6 (2)	12 (3)
Metabolism and nutritional disorders	8 (2)	9 (2)
Skin and subcutaneous tissue disorders	3 (1)	7 (2)
Blood and lymphatic system disorders	1 (0.3)	7 (2)
**Serious adverse events**		
Any serious adverse event	126 (3)	146 (4)
Most common (≥1 per 1000 patient-months of follow-up in high dose arm)		
Infections and infestations	33 (9)	25 (6)
Cardiac disorders	22 (6)	18 (5)
Vascular disorders	13 (3)	7 (2)
Surgical and medical procedures	8 (2)	9 (2)
Respiratory, thoracic and mediastinal disorders	9 (2)	7 (2)
Neoplasms (benign, malignant, unspecified)	6 (1)	5 (1)
Gastrointestinal disorders	7 (2)	4 (1)
Injury, poisoning and procedural complications	7 (2)	3 (1)
Metabolism and nutritional disorders	7 (2)	3 (1)

Adverse events are reported as number of events (incidence rate per 1000 per 1000 patient-months of follow-up). Adverse events organized by System Organ Class (MedDRA Terminology).

Overall, 358 (54.5%) patients completed the health-related quality of life questionnaire at baseline (Table 5). A difference of 5 points or more in a domain was considered to indicate a minimally-important clinical difference. [23] During treatment, there was a higher domain score favoring low dose therapy for physical functioning (7.0 points, 95% CI 0.6 to 13.4, p = 0.04), role limitations (emotional) (13.6 points, 95% CI 0.5 to 26.7, p = 0.04), and physical composite score (2.4 points, 95% CI 0.1 to 4.7, p = 0.04).

**Table 5:** Kidney Disease Quality of Life Short Form (KDQOL SF-36) domains.

SF-36 domain	Baseline		12 months		Difference between low dose and high dose group over time (12 months versus baseline; delta of delta [DD]) (95% CI) [24]†	p value
	Low dose group*	High dose group*	Low dose group*	High dose group*		
Physical functioning	51.0 (2.4)	52.0 (2.2)	52.3 (2.8)	46.4 (2.8)	7.0 (0.6 to 13.4)	0.03
Burden of kidney disease	34.6 (1.8)	36.0 (1.8)	38.6 (2.3)	37.4 (2.2)	2.6 (-3.5 to 8.7)	0.40
Quality of social interaction	71.3 (1.6)	71.1 (1.6)	70.1 (2.1)	73.3 (2.0)	-3.5 (-9.3 to 2.3)	0.24
Cognitive function	76.1 (1.6)	73.9 (1.6)	74.9 (2.1)	74.8 (2.0)	-2.2 (-8.0 to 3.6)	0.46
Symptoms	75.5 (1.2)	77.7 (1.2)	81.8 (1.6)	80.7 (1.5)	3.3 (-1.2 to 7.8)	0.15
Effects of kidney disease	67.0 (1.6)	65.2 (1.6)	68.4 (2.1)	67.8 (2.0)	-1.3 (-6.9 to 4.3)	0.66
Sleep	60.1 (1.6)	60.6 (1.6)	62.0 (2.1)	63.6 (2.0)	-1.0 (-6.8 to 4.8)	0.73
Social support	69.2 (2.1)	71.0 (2.1)	63.3 (2.9)	67.3 (2.8)	-2.1 (-11.0 to 6.8)	0.64
Work status	23.5 (2.7)	23.7 (2.7)	30.7 (3.4)	26.7 (3.3)	4.2 (-4.9 to 13.3)	0.36
Dialysis staff encouragement	87.4 (1.3)	87.7 (1.3)	90.8 (1.6)	87.7 (1.6)	3.5 (-1.0 to 8.0)	0.13
Overall health	56.1 (1.7)	56.3 (1.7)	59.3 (2.1)	56.3 (2.1)	3.2 (-2.6 to 9.0)	0.29
Patient satisfaction	74.8 (1.5)	75.6 (1.5)	73.7 (1.9)	74.1 (1.9)	0.5 (-4.8 to 5.8)	0.86
Role limitations, physical	37.0 (3.2)	37.7 (3.2)	48.8 (4.3)	41.4 (4.2)	8.4 (-4.4 to 21.2)	0.20
Pain	60.7 (2.1)	61.7 (2.1)	67.5 (2.7)	63.2 (2.7)	5.2 (-2.5 to 12.9)	0.18
General health	37.5 (1.5)	39.1 (1.5)	38.4 (2.0)	38.6 (1.9)	1.4 (-3.9 to 6.7)	0.61
Emotional well-being	62.8 (1.6)	62.0 (1.6)	62.1 (2.0)	62.9 (2.0)	-1.6 (-7.0 to 3.8)	0.56
Role limitations, emotional	49.0 (3.4)	49.3 (3.4)	63.7 (4.5)	50.4 (3.4)	13.6 (0.5 to 26.7)	0.04
Social function	63.9 (1.9)	64.0 (1.9)	62.5 (2.5)	62.9 (2.4)	-0.3 (-6.9 to 6.3)	0.94
Energy/fatigue	47.3 (1.7)	47.4 (1.7)	47.7 (2.1)	48.3 (2.1)	-0.5 (-6.3 to 5.3)	0.87
Physical composite	35.9 (0.8)	36.6 (0.8)	38.2 (1.0)	36.5 (1.0)	2.4 (0.1 to 4.7)	0.04
Mental composite	44.5 (0.8)	44.2 (0.8)	45.1 (1.1)	45.2 (1.1)	-0.4 (-3.3 to 2.5)	0.78

*Data are estimated means (standard error of mean).

^†^Data are unadjusted means (delta of delta indicating difference between groups from baseline to 12 months) and 95% CI.

A lower score is indicative of a lower quality of life. For example, a lower pain score is indicative of a higher level of self-reported pain. Each domain has a maximum score of 100; a difference of 5 points or more in a domain score may indicate a minimally-important clinical difference. A positive delta of delta indicates a higher quality of life score with fixed low dose therapy. Data are reported in the patients who provided data for all quality of life outcomes at baseline (n = 179 in each group). Data were not reported for sexual function domain as there were only 17 patients in the high dose group and 22 patients in the low dose group who provided scores for this outcome. The p value indicates the difference between low dose and high dose treatment over time.

Participants assigned to low dose therapy experienced a higher risk of red cell blood transfusion (HR 2.44, 1.23–4.76, p = 0.007; Table 3). The mean (SD) hemoglobin at the time of first blood transfusion was 7.68 (0.85) g/dl in the low dose group and 7.95 (0.74) g/dl in the high dose group (p = 0.10).

There was no evidence of differences in pre-dialysis systolic or diastolic blood pressure between treatment groups (S4 Table).

In subgroup analyses for the composite endpoint of death from any cause, nonfatal myocardial infarction, nonfatal stroke, or hospitalization for cardiovascular cause, no differential effects based on participant age, gender, history of diabetes, or pre—existing ischemic heart disease were observed (S5 Table). When analyses for the composite endpoint were restricted to patients with ESA resistance at randomization (n = 100), there was no difference in the risk of the primary endpoint comparing low dose with high dose ESA therapy (HR 0.93, 95% CI 0.43–2.00, p = 0.84).

## Discussion

The primary objective of the C.E. DOSE trial was initially to determine whether treatment of anemia with fixed low dose ESA to treat anemia could mitigate the risk of death and major cardiovascular events among people with end-stage kidney disease while maintaining health-related quality of life compared with fixed high dose treatment. Findings from this study showed that a strategy to prescribe fixed low dose ESA in patients treated with dialysis was not feasible within a trial setting and that fixed dose therapy had uncertain effects on serum iron and inflammation markers. The study was not able to address whether fixed dose therapy had effects on mortality, cardiovascular events, or health-related quality of life.

A fixed low dose approach to ESA therapy has been postulated to improve hemoglobin levels (and potentially patient quality of life) while avoiding the harms associated with high ESA doses required to achieve complete anemia correction.[25] The results of C.E. DOSE showing no evidence of difference between low and high fixed dose ESA strategies on cardiovascular endpoints are congruent with the TREAT trial, a large-scale randomized trial comparing darbepoetin alfa treatment aiming for a hemoglobin target of approximately 13 g/dl versus placebo with rescue darbepoetin alfa therapy if the hemoglobin level fell below 9 g/dl in 4038 patients with type 2 diabetes and CKD.[26] In that study, there was no significant difference in the risk of a composite of death or cardiovascular events in the group assigned to darbepoetin alfa and no clinical improvement in any quality of life domain, although there were modest improvements in patient-reported fatigue and an absolute reduction in the proportion of patients receiving red cell blood transfusions during 29.1 months of follow up. Taken together, existing trials and the C.E. DOSE study indicate that for many patients with CKD, higher hemoglobin targets reduce life expectancy while it is uncertain whether low fixed ESA dose strategies can mitigate risks of death or cardiovascular events due to the difficulties in sustaining high fixed dose therapy within a trial setting. The benefits of higher hemoglobin targets or fixed high ESA dose therapy appear limited to the avoidance of blood transfusions. There is no evidence from the C.E. DOSE study and previous trials that a higher hemoglobin level or fixed ESA dose clearly improves quality of life,[27] and this indication alone may not be an appropriate reason to commence or continue ESA therapy targeting higher hemoglobin levels.

The C.E. DOSE study highlights the considerable challenge of recruiting participants to a trial evaluating fixed dose ESA in an era of changing practice patterns, increasing restraint in recommended hemoglobin targets, and US Food and Drug Administration cautions about ESA therapy.[20] As the C.E. DOSE trial investigators secured funding in 2006,[18] evidence of increased all-cause mortality with higher hemoglobin treatment targets was yet to emerge[10] and all existing trials of ESAs evaluated hemoglobin targets, rather than different dose approaches.[11] Secondary analyses of existing trials subsequently showed an association between high doses of ESA therapy and increased risks of death, myocardial infarction, and heart failure,[13] suggesting the need to examine the effectiveness and safety of ESA fixed dose strategies rather than hemoglobin targets in ESA trials. During the recruitment phase of C.E. DOSE, the TREAT trial reported showing no advantage for ESA treatment to a higher hemoglobin target versus placebo (with rescue darbepoetin alfa therapy),[26] and the cumulative evidence[10, 11] resulted in recommendations in 2011 from the US Food and Drug Administration for more conservative ESA dosing, warning that no trial had identified a safe hemoglobin target level, ESA dose, or dosing strategy.[20] Clinical guidelines in 2012 similarly became more conservative than previous, suggesting ESA therapy was used to avoid hemoglobin levels below 9.0 g/deciliter.[12] The practice change from near-full anemia correction to ESA therapy administered by weighing the balance of harms (mortality and cardiovascular events) with expected benefits (quality of life and avoidance of transfusions) for individual patients during the conduct of C.E. DOSE resulted in lower levels of clinician investigator equipoise than initially expected, slow recruitment, and lower ESA doses given than specified in the trial protocol, leading to loss of ESA dose separation between the two treatment groups.

Although there was no statistical difference between treatment doses on mortality and cardiovascular events, there was an increased risk of blood transfusion among patients assigned to low dose treatment. In the dialysis setting, information from this study and the TREAT trial together might suggest that patients for whom a blood transfusion might be particularly disadvantageous (such as potential recipients of a kidney transplant who wish to minimize allo-sensitization or those with iron overload diseases) might reasonably consider short-term ESA therapy at a fixed dose when the hemoglobin level is below 9 g/dl. Patients might also appropriately consider avoiding ESA therapy even with the potential to minimize risk of blood transfusions due to the uncertain risks of mortality and cardiovascular events and evidence showing increased cardiovascular events including stroke with a higher hemoglobin target.[10, 11]

This trial provides short-term data on the use of a fixed dose ESA strategy in adults with end-stage kidney disease. It is the first and only ESA fixed dose trial to date beyond initial proof of concept studies[28, 29] and was multicenter, including academic and non-academic centers to maximize applicability to a range of clinical settings and practices. However, the trial has limitations that should be considered when assessing the implications for clinical practice. First, the trial was recruited to 31% of the intended target and therefore had inadequate statistical power to provide conclusive data about the effect of fixed low dose ESA therapy on mortality and major cardiovascular events. Beneficial or harmful effects of fixed dose ESA strategies cannot be ruled in or out based on these results. Reduced planned follow up from 4 years to 12 months additionally restricted the power of the study to provide information about the longer-term implications of fixed low dose ESA. In addition, treatment doses in the high and low dose groups started to converge at three months, coinciding with the peak hemoglobin levels observed among patients in the high dose arm. At study end, complete convergence of hemoglobin levels and near merging of ESA dose reduced the separation of the treatment strategies in each arm and accordingly the potential for the trial to discern clinical effects of different fixed ESA doses. It is possible that a trial design with a lower planned dose in both arms (for example, 4000 IU/weekly epoetin alfa or beta (as high dose) versus placebo (as low dose)) might have prevented a loss of dose separation due to smaller increases in hemoglobin levels in the higher treatment arm and greater adherence to a study protocol in the context of more conservative practice for anemia care in the dialysis setting. Another limitation was the lack of masking to treatment dose for investigators that may have impacted on clinical decisions to administer blood transfusions or alter treatment doses. However, there was no statistical difference in the hemoglobin level at which blood transfusions were administered to patients in the high dose compared to low dose ESA groups.

In conclusion, fixed low dose ESA therapy had uncertain effects on serum iron and inflammation markers, all-cause mortality and cardiovascular events and health-related quality of life. Maintaining a fixed ESA dose strategy for 12 months was not feasible in a trial setting.

## Supporting information

S1 Appendix Ethics committees. (DOCX)

S2 Appendix Study investigators and contributors. (DOCX)

S3 Appendix CONSORT 2010 checklist of information to include when reporting a randomized trial. (DOCX)

S4 Appendix Therapeutic algorithm for management of ESA dose and hemoglobin levels. (DOCX)

S5 Appendix Non-randomized standards of care. (DOCX)

S6 Appendix Definitions of study endpoints. (DOCX)

S1 File Study protocol (English version). (PDF)

S2 File Study protocol (Italian version). (PDF)

S3 File Raw study data. Table A. Baseline characteristics and clinical outcomes. Table B. Adverse events. Table C. Serious adverse events. Table D. Legend.
(ZIP)

S1 Table Intervention practice characteristics. Data are mean (standard error of mean), median (interquartile range), or number (proportion). Hemoglobin level stabilization was defined as two consecutive measurements between 10 and 12 g/dL.
(DOCX)

S2 Table Adverse events. (DOCX)

S3 Table Serious adverse events. (DOCX)

S4 Table Blood pressure endpoints. ^a^ Data are unadjusted means (and standard error). ^b^ Data are unadjusted means (difference between groups from baseline to 12 months; delta of delta) and 95% confidence interval. The p value indicates the difference between low dose and high dose treatment over time (delta of delta [DD]).
(DOCX)

S5 Table Subgroup analyses for composite endpoint of death from any cause, nonfatal myocardial infarction, nonfatal stroke, or hospitalization for cardiovascular cause. Data are expressed as frequency and percent.
(DOCX)

## References

[1] Winearls CG, Oliver DO, Pippard MJ, Reid C, Downing MR, Cotes PM. Effect of human erythropoietin derived from recombinant DNA on the anaemia of patients maintained by chronic haemodialysis. Lancet. 1986;2(8517):1175–8. 2877323 10.1016/s0140-6736(86)92192-6

[2] Association between recombinant human erythropoietin and quality of life and exercise capacity of patients receiving haemodialysis. Canadian Erythropoietin Study Group. BMJ. 1990;300(6724):573–8. 2108751 10.1136/bmj.300.6724.573 PMC1662387

[3] Bahlmann J, Schoter KH, Scigalla P, Gurland HJ, Hilfenhaus M, Koch KM, et al Morbidity and mortality in hemodialysis patients with and without erythropoietin treatment: a controlled study. Contrib Nephrol. 1991;88:90–106. 2040200 10.1159/000419519

[4] Zins B, Drueke T, Zingraff J, Bererhi L, Kreis H, Naret C, et al Erythropoietin treatment in anaemic patients on haemodialysis. Lancet. 1986;2(8519):1329. 10.1016/s0140-6736(86)91449-2 2878188

[5] Locatelli F, Pisoni RL, Combe C, Bommer J, Andreucci VE, Piera L, et al Anaemia in haemodialysis patients of five European countries: association with morbidity and mortality in the Dialysis Outcomes and Practice Patterns Study (DOPPS). Nephrol Dial Transplant. 2004;19(1):121–32. 14671047 10.1093/ndt/gfg458

[6] Foley RN, Parfrey PS, Harnett JD, Kent GM, Murray DC, Barre PE. The impact of anemia on cardiomyopathy, morbidity, and and mortality in end-stage renal disease. Am J Kidney Dis. 1996;28(1):53–61. 8712222 10.1016/s0272-6386(96)90130-4

[7] Collins AJ, Ma JZ, Xia A, Ebben J. Trends in anemia treatment with erythropoietin usage and patient outcomes. Am J Kidney Dis. 1998;32(6 Suppl 4):S133–41. 9892380 10.1016/s0272-6386(98)70176-3

[8] Besarab A, Bolton WK, Browne JK, Egrie JC, Nissenson AR, Okamoto DM, et al The effects of normal as compared with low hematocrit values in patients with cardiac disease who are receiving hemodialysis and epoetin. N Engl J Med. 1998;339(9):584–90. 10.1056/NEJM199808273390903 9718377

[9] Singh AK, Szczech L, Tang KL, Barnhart H, Sapp S, Wolfson M, et al Correction of anemia with epoetin alfa in chronic kidney disease. N Engl J Med. 2006;355(20):2085–98. 10.1056/NEJMoa065485 17108343

[10] Phrommintikul A, Haas SJ, Elsik M, Krum H. Mortality and target haemoglobin concentrations in anaemic patients with chronic kidney disease treated with erythropoietin: a meta-analysis. Lancet. 2007;369(9559):381–8. 10.1016/S0140-6736(07)60194-9 17276778

[11] Palmer SC, Navaneethan SD, Craig JC, Johnson DW, Tonelli M, Garg AX, et al Meta-analysis: erythropoiesis-stimulating agents in patients with chronic kidney disease. Ann Intern Med. 2010;153(1):23–33. 10.7326/0003-4819-153-1-201007060-00252 20439566

[12] Kidney Disease: Improving Global Outcomes (KDIGO) Anemia Work Group. KDIGO Clinical Practice Guideline for Anemia in Chronic Kidney DIsease. Kidney Int Suppl. 2012;2:279–335.

[13] Szczech LA, Barnhart HX, Inrig JK, Reddan DN, Sapp S, Califf RM, et al Secondary analysis of the CHOIR trial epoetin-alpha dose and achieved hemoglobin outcomes. Kidney Int. 2008;74(6):791–8. 10.1038/ki.2008.295 18596733 PMC2902279

[14] Bellinghieri G, Condemi CG, Saitta S, Trifiro G, Gangemi S, Savica V, et al Erythropoiesis-stimulating agents: dose and mortality risk. J Ren Nutr. 2015;25(2):164–8. 10.1053/j.jrn.2014.10.012 25529282

[15] Inrig JK, Bryskin SK, Patel UD, Arcasoy M, Szczech LA. Association between high-dose erythropoiesis-stimulating agents, inflammatory biomarkers, and soluble erythropoietin receptors. BMC Nephrol. 2011;12:67 10.1186/1471-2369-12-67 22152013 PMC3254065

[16] Strippoli GF. Effects of the dose of erythropoiesis stimulating agents on cardiovascular events, quality of life, and health-related costs in hemodialysis patients: the clinical evaluation of the dose of erythropoietins (C.E. DOSE) trial protocol. Trials. 2010;11:70 10.1186/1745-6215-11-70 20534124 PMC2903576

[17] Hansson L, Hedner T, Dahlof B. Prospective randomized open blinded end-point (PROBE) study. A novel design for intervention trials. Prospective Randomized Open Blinded End-Point. Blood Press. 1992;1(2):113–9. 1366259 10.3109/08037059209077502

[18] Italian Medicines Agency (AIFA) Research & Development Working Group. Feasibility and challneges of independent research on drugs: the Italian Medicines Agency (AIFA) experience. Eur J Clin Invest. 2009;40(1):69–86. 10.1111/j.1365-2362.2009.02226.x 20055898

[19] Fellstrom BC, Jardine AG, Schmieder RE, Holdaas H, Bannister K, Beutler J, et al Rosuvastatin and cardiovascular events in patients undergoing hemodialysis. N Engl J Med. 2009;360(14):1395–407. 10.1056/NEJMoa0810177 19332456

[20] US Food and Drug Administration. FDA Drug Safety Communication: Modified Dosing Recommendations to Improve the Safe Use of Erythropoiesis-Stimulating Agents in Chronic Kidney Disease, 2011. http://www.fda.gov/Drugs/DrugSafety/ucm259639.htm. 2011.

[21] Singer J, Willett J. Applied longitudinal data analysis: Modeling change and event occurrence. New York: Oxford University Press, 2003.

[22] Johnson DW, Pascoe EM, Badve SV, Dalziel K, Cass A, Clarke P, et al A randomized, placebo-controlled trial of pentoxifylline on erythropoiesis-stimulating agent hyporesponsiveness in anemic patients with CKD: the Handling Erythropoietin Resistance With Oxpentifylline (HERO) trial. Am J Kidney Dis. 2015;65(1):49–57. 10.1053/j.ajkd.2014.06.020 25115616

[23] Wyrwich KW, Tierney WM, Babu AN, Kroenke K, Wolinsky FD. A comparison of clinically important differences in health-related quality of life for patients with chronic lung disease, asthma, or heart disease. Health Serv Res. 2005;40(2):577–91. 15762908 10.1111/j.1475-6773.2005.00373.x PMC1361158

[24] Hays R, Kallich J, Mapes D, Coons S, Amin N, Carter W, et al Kidney Disease Quality of Life Short Form (KDQOL-SFTM), Version 1.3: A manual for use and scoring. Santa Monica, CA: RAND, P-7994 1995.

[25] Zhang Y, Thamer M, Kaufman JS, Cotter DJ, Hernan MA. High doses of epoetin do not lower mortality and cardiovascular risk among elderly hemodialysis patients with diabetes. Kidney Int. 2011;80(6):663–9. 10.1038/ki.2011.188 21697811 PMC3637948

[26] Pfeffer MA, Burdmann EA, Chen CY, Cooper ME, de Zeeuw D, Eckardt KU, et al A trial of darbepoetin alfa in type 2 diabetes and chronic kidney disease. N Engl J Med. 2009;361(21):2019–32. 10.1056/NEJMoa0907845 19880844

[27] Clement FM, Klarenbach S, Tonelli M, Johnson JA, Manns BJ. The impact of selecting a high hemoglobin target level on health-related quality of life for patients with chronic kidney disease: a systematic review and meta-analysis. Arch Intern Med. 2009;169(12):1104–12. 10.1001/archinternmed.2009.112 19546410

[28] Eschbach JW, Egrie JC, Downing MR, Browne JK, Adamson JW. Correction of the anemia of end-stage renal disease with recombinant human erythropoietin. Results of a combined phase I and II clinical trial. N Engl J Med. 1987;316(2):73–8. 10.1056/NEJM198701083160203 3537801

[29] Eschbach JW, Abdulhadi MH, Browne JK, Delano BG, Downing MR, Egrie JC, et al Recombinant human erythropoietin in anemic patients with end-stage renal disease. Results of a phase III multicenter clinical trial. Ann Intern Med. 1989;111(12):992–1000. 2688507 10.7326/0003-4819-111-12-992

