# Supplementary material for: Low versus high dose erythropoiesis-stimulating agents in hemodialysis patients with anemia: A randomized clinical trial
Source: PLoS One. 2017 Mar 1;12(3):e0172735. doi: 10.1371/journal.pone.0172735 (PMC5332066; doi:10.1371/journal.pone.0172735)
Supplement: S1 Appendix — (DOCX) [file pone.0172735.s001.docx]

## S1 Appendix. Ethics committees.

| **Clinic name** | **Name of approval committee** | **Approval date** |
| --- | --- | --- |
| Presidio Ospedaliero A. Perrino, Brindisi | Comitato indipendente di etica medica della ASL BR di Brindisi | 10/31/2008 |
| Ospedale Sant'Anna, San Fermo Battaglia, Como | Comitato Etico dell'Azienda ospedaliera Sant'Anna di Como | 6/16/2011 |
| Ospedale Nuovo Sant'Anna, Cona, Ferrara | Comitato Etico della provincia di Ferrara | 9/30/2010 |
| Ospedale S. Giovanni Di Dio, Agrigento | Comitato Etico dell'ASP 1 Agrigento | 9/10/2012 |
| Ospedale G. Bernabeo, Ortona | Comitato Etico dell'Universit degli Studi "G. d'Annunzio" di Chieti-Pescara e della ASL Lanciano-Vasto-Chieti | 5/26/2011 |
| Ospedale S. Eugenio ASL RMC, Roma | Comitato Etico dell'ASL Roma C | 6/15/2009 |
| Ospedale Civile di Alghero ASL n°1, Alghero, Sassari | Comitato di bioetica dell'ASL di Sassari | 3/24/2010 |
| P.O. SUD - Formia ASL Latina, Formia, Latina | Comitato Etico dell'AUSL Latina | 3/24/2011 |
| Ospedale San Giacomo, Novi Ligure, Alessandria | Comitato Etico dell'Azienda ospedaliera SS Antonio e Biagio e Cesare Arrigo di Alessandria | 3/17/2009 |
| Ospedale Alfredo Fiorini di Terracina, Terracina, Latina | Comitato Etico Lazio 2 | 3/24/2011 |
| Policlinico S. Orsola – Malpighi, Bologna | Comitato Etico indipendente dell'Azienda ospedaliera-universitaria policlinico Sant'Orsola Malpighi di Bologna | 10/6/2009 |
| Centro Dialitico Diaverum Marsala, Trapani | Comitato Etico azienda sanitaria di Trapani | 6/7/2011 |
| Centro di Emodialisi ausl Parma, Parma | Comitato Etico unico per la provincia di Parma | 11/8/2011 |
| Ospedale A Landolfi, Solofra, Avellino | Comitato Etico della ASL di Avellino | 5/29/2012 |
| Ospedale Renzetti ASL Lanciano Vasto, Lanciano, Chieti | Comitato Etico dell'università degli Studi Gabriele D`Annunzio e della Asl 2 Lanciano-Vasto-Chieti di Chieti | 11/11/2010 |
| Ospedale S. Giovanni di Dio di Gorizia, Gorizia | Comitato Etico Indipendente dell'Azienda per i Servizi Sanitari 2 Isontina di Gorizia | 12/1/2011 |
| Centro Dialitico Diaverum, Riesi, Caltanissetta | Comitato Etico dell'ASP Caltanissetta | 12/15/2011 |
| Centro Dialitico Diaverum, Ladispoli, Roma | Comitato Etico ASL Roma/F | 7/1/2009 |
| Arcispedale S. Maria Nuova, Reggio Emilia | Comitato Etico dell'Azienda ospedaliera di Reggio Emilia | 5/23/2011 |
| Azienda ospedaliera Ospedale Civile di Legnano, Legnano, Milano | Comitato Etico dell'Azienda ospedaliera ospedale civile di Legnano | 6/21/2011 |
| Ospedale Beato Angelo, Acri, Cosenza | Comitato Etico dell'ASP di Cosenza | 5/22/2012 |
| Ospedale Bellaria, Bellaria, Bologna | Comitato Etico indipendente dell'ASLdi Bologna | 3/25/2010 |
| Ospedale di Nicosia, Nicosia, Enna | Comitato Etico dell'AUSL N 4 Enna | 5/21/2009 |
| Azienda ospedaliera universitaria di Parma, Parma | Comitato Etico unico per la provincia di Parma | 11/8/2011 |
| S. Pio da Pietrelcina, Vasto, Chieti | Comitato Etico dell'Universit degli Studi "G. d'Annunzio" di Chieti-Pescara e della ASL Lanciano-Vasto-Chieti | 11/11/2010 |
| Ospedale di Manduria, Manduria, Taranto | Comitato indipendente di etica medica della ASL BR di Brindisi | 6/23/2009 |
| Ospedale Maggiore di Chieri ASL TO 5, Chieri, Torino | Comitato Etico dell'Azienda ospedaliera San Luigi Gonzaga | 3/29/2010 |
| Arnas civico Di Cristina, Palermo | Comitato di Bioetica dell'Azienda Ospedaliera Civico e Benfratelli - Giovanni Di Cristina - Maurizio Ascoli di Palermo | 3/26/2012 |
| Ospedale SS Annunziata, Sassari | Comitato Etico dell'ASL di Sassari | 3/8/2012 |
| Ospedale Fornaroli, Magenta, Milano | Comitato Etico dell'Azienda ospedaliera ospedale civile di Legnano | 6/14/2012 |
| Azienda Ospedaliera C.T.O./C.R.F./ M. Adelaide, Torino | Comitato Etico indipendente dell'Azienda ospedaliera-universitaria San G. Battista di Torino | 4/20/2009 |
| P.P.I. Priverno, Priverno, Latina | Comitato Etico Lazio 2 | 3/24/2011 |
| Ospedali Riuniti di Anzio e Nettuno, Anzio, Roma | Comitato Etico ASL Roma/H | 5/28/2012 |
| Ospedale San Giovanni Bosco, Torino | Comitato Etico della ASL di Torino 2 | 7/20/2010 |
| Ospedale S. Barbara, Rogliano, Cosenza | Comitato Etico dell'ASP di Cosenza | 5/22/2012 |
| Azienda Ospedaliera "Ospedali Riuniti di Foggia", Foggia | Comitato Etico dell'azienda Ospedaliero-Universitaria Ospedali Riuniti di Foggia | 4/29/2009 |
| Ospedale Valle D'Itria ASL TA, Martina Franca, Taranto | Comitato Etico indipendente dell'ASL di Taranto | 1/20/2011 |
| Istituto Clinico Humanitas, Rozzano, Milano | Comitato Etico indipendente istituto clinico Humanitas | 6/8/2012 |
| Policlinico San Donato, San Donato Milanese, Milano | Comitato Etico indipendente dell'ASL di Milano 2 | 7/24/2012 |
| Ospedale S. Maria degli Angeli, Pordenone | Comitato Etico indipendente dell'Azienda ospedaliera Santa Maria degli angeli di Pordenone | 3/27/2009 |
| Jesi (Carlo Urbani), Jesi, Ancona | Comitato Etico indipendente dell'Azienda Sanitaria Unica regionale di Ancona | 11/9/2011 |
