# Supplementary material for: Low versus high dose erythropoiesis-stimulating agents in hemodialysis patients with anemia: A randomized clinical trial
Source: PLoS One. 2017 Mar 1;12(3):e0172735. doi: 10.1371/journal.pone.0172735 (PMC5332066; doi:10.1371/journal.pone.0172735)
Supplement: S2 Appendix — (DOCX) [file pone.0172735.s002.docx]

## S2 Appendix. Study investigators and contributors.

### Principal investigator

**Prof Giovanni FM Strippoli**, Department of Emergency and Organ Transplantation, University of Bari, Piazza Giulio Cesare, 70124 Bari, Italy; Sydney School of Public Health University of Sydney, Australia. Professor of nephrology, overall lead for study design, implementation, funding, data analysis and reporting. Overall guarantor for study.

### Trial coordinator

**Valeria Saglimbene**, MSc, PhD candidate, Sydney School of Public Health, University of Sydney, Australia (current affiliation); previously Diaverum Renal Services Group (Lund, Sweden) and Fondiazione Mario Negri Sud (S. Maria Imbaro, Chieti, Italy)

### Trial Steering Committee

**Prof Giovanni FM Strippoli**, Department of Emergency and Organ Transplantation, University of Bari, Piazza Giulio Cesare, 70124 Bari, Italy; Sydney School of Public Health University of Sydney, Australia (Chair); **Dr Suetonia C. Palmer**, University of Otago Christchurch, New Zealand; **Prof Jonathan C. Craig**, School of Public Health University of Sydney, Australia; **Dr Salvatore Di Giulio,** A.O. San Camillo Forlanini, Rome, Italy; **Prof Antonio Nicolucci**, Center for Outcomes Research and clinical Epidemiology (CORESEARCH), Italy; **Dr Fabio Pellegrini,** Global Medical Biogen Idec, Cambridge, MA, USA; **Dr Deni A. Procaccini**, Division of Nephrology, Riuniti Hospital, Foggia, Italy; **Dr Paolo Strippoli,** Department of Nephrology and Dialysis, Presidio Ospedaliero " A. Perrino", Brindisi, Italy; **Dr Antonio Santoro**, Department of Npehrology and Dialysis, Policlinico S. Orsola, Malpighi, Bologna, Italy; **Prof Gianni Tognoni,** Consorzio Mario Negri, Milan, Italy

### Co-investigators

**Valeria Saglimbene;** study conduct, data analysis, drafting of the report; **Suetonia C Palmer;** design, data analysis, drafting of the report; **Prof Jonathan C Craig,** design, data analysis, intellectual review of study report; **Marinella Ruospo,** study conduct, data analysis, intellectual review of study report; **Prof Antonio Nicolucci,** design, data analysis, intellectual review of study report; **Prof Marcello Tonelli,** design, data analysis, intellectual review of study report; **Prof David Johnson,** design, data analysis, intellectual review of study report; **Giuseppe Lucisano,** design, data analysis, intellectual review of study report; **Gabrielle Williams,** data analysis, intellectual review of study report; **Miriam Valentini**, study conduct, data analysis, intellectual review of study report; **Daniela D’Alonzo,** study conduct, data analysis, intellectual review of study report; **Fabio Pellegrini,** design, supervision of data analysis, intellectual review of study report; **Prof Paolo Strippoli,** study conduct, data analysis, intellectual review of study report; **Mario Salomone,** study conduct, data analysis, intellectual review of study report; **Antonio Santoro,** study conduct, data analysis, intellectual review of study report; **Stefano Maffei,** study conduct, data analysis, intellectual review of study report; **Prof Jörgen Hegbrant,** data analysis, intellectual review of study report; **Prof Gianni Tognoni,** design, data analysis, intellectual review of study report; **Prof Giovanni F.M. Strippoli,** design, study conduct, data analysis, intellectual review of study report;

### Trial coordinating center

Fondazione Mario Negri Sud, Santa Maria Imbaro (Chieti), Italy

**Valeria Saglimbene** (project management); **Barbara Di Nardo, Riccarda Memmo, Cristina Di Biase, Giuliana Di Nardo and Rosalia Di Lallo** (database design, data management)

### Clinical Research Management and Monitoring

**Miriam Valentini, Daniela D’Alonzo**, Department of Clinical Pharmacology and Epidemiology, Unit of Clinical Research Management and Monitoring, Fondazione Mario Negri Sud, Santa Maria Imbaro, Italy; **Maria Celeste Pirozzoli,** Unit of Clinical Research Management and Monitoring, Fondazione Mario Negri Sud, Santa Maria Imbaro, Italy

### Data Safety Monitoring Committee Independent Members

**Dr. Massimiliano Copetti**, Unit of Biostatistics, IRCCS Casa Sollievo della Sofferenza, San Giovanni Rotondo, Italy (statistician); **Dr. Giacomo Vespasiani**, Diabetes Unit, Madonna del Soccorso Hospital, San Benedetto del Tronto, Italy; **Dr. Angelo Marco Murgo**, Centro Rene Nissoria, Enna, Italy; **Dr Giuseppina Montalto**, Centro Rene Palagonia, Catania, Italy; **Dr Salvatore Pagano**, Centro Rene Klotho, Riesi, Italy.

### Local Researchers

**Ospedale Sant'Anna, San Fermo Battaglia, Como**, Stefano Mangano, Antonio Bellasi, Daniela Martinelli, Barbara Napoli; **Ospedale Nuovo Sant'Anna, Cona, Ferrara,** Giorgia Russo, Silvia Forcellini; **Ospedale S. Giovanni Di Dio, Agrigento,** Anna Clementi, Michele Casà; **Ospedale G. Bernabeo, Ortona,** Benito D’Angelo, Ludovico Caravelli; **Ospedale S. Eugenio ASL RMC, Roma,** Michele Ferrannini, Damiano Di Franco, Alessia Centi; **Ospedale Civile di Alghero ASL n°1, Alghero, Sassari,** Angelo Piras, Michelina Cherchi, Leopoldo Di Lauro, Annamaria Ginanni; **P.O. SUD - Formia ASL Latina, Formia, Latina,** Antonio Treglia; **Ospedale San Giacomo, Novi Ligure, Alessandria,** Elena Gori, **Policlinico S. Orsola – Malpighi, Bologna**, Elena Mancini, Marco Veronesi, **Centro di Emodialisi ausl Parma, Parma,** Davide Gerra, **Ospedale Renzetti  ASL Lanciano Vasto, Lanciano, Chieti,** Gabriella Catucci, Meriateresa Cuonzo; **Ospedale S. Giovanni di Dio di Gorizia, Gorizia,** Massimo Martone, Dorina Brindusa Berbecar; **Centro Dialitico Diaverum, Ladispoli, Roma,** Alberto De Luca, Michela Tedesco, Damano Di Franco; **Azienda ospedaliera Ospedale Civile di Legnano, Legnano, Milano,** Valter Barzaghi; **Ospedale Beato Angelo, Acri, Cosenza,** Romano Musacchio; **Ospedale Bellaria, Bellaria, Bologna**, Marcora Mandreoli, Antonia Lopez, Elena Sestigiani; **Azienda Ospedaliera Universitaria di Parma, Parma;** Lara Zerbini, Camilla Gambaretto; **S. Pio da Pietrelcina, Vasto, Chieti,** Bice Giammichele, Lucia Perilli, Assunta Antinucci; **Ospedale di Manduria, Manduria, Taranto,** Luigi Vernaglione, Viviana Nosella; **Ospedale Maggiore di Chieri ASL TO 5, Chieri, Torino,** Emanuele Stramignoni, Marina D’Amicone, Giovanni Grott, Carla Buniva, Giancarlo Priasca, Giuseppe Squiccimarro; **Arnas Civico Di Cristina, Palermo,** Carmela Zagarrigo, Onofrio Schillaci, Franca Servillo; **Ospedale SS Annunziata, Sassari,** Giovanni Francesco Branca; **Azienda Ospedaliera C.T.O./C.R.F./ M. Adelaide, Torino,** Daniela Bergamo, Stefano Maffei; **Ospedali Riuniti di Anzio e Nettuno, Anzio, Roma,** Giovanni Michele Fabozzi; **Ospedale San Giovanni Bosco, Torino,** Simona Borsa, Paola Mesiano, Pasqualina Cecere; **Ospedale S. Barbara, Rogliano, Cosenza**, Lucia Palma; **Azienda Ospedaliera "Ospedali Riuniti di Foggia", Foggia**, Rossella Perulli, Dora Porcelluzzi, Adelaide Di Lorenzo; **Ospedale Valle D'Itria ASL TA, Martina Franca, Taranto,** Daniela Mezzopane, Anna Maria Maiorano, Michele Rossini; **Policlinico San Donato, San Donato Milanese, Milano,** Angela Aiello, Chiara Rosa Ronga; **Ospedale S. Maria degli Angeli, Pordenone,** Michele Cimolino; **Jesi (Carlo Urbani), jesi, Ancona,** Tania Monteburini.
