# Supplementary material for: Low versus high dose erythropoiesis-stimulating agents in hemodialysis patients with anemia: A randomized clinical trial
Source: PLoS One. 2017 Mar 1;12(3):e0172735. doi: 10.1371/journal.pone.0172735 (PMC5332066; doi:10.1371/journal.pone.0172735)
Supplement: S4 Appendix — (DOCX) [file pone.0172735.s004.docx]

## S4 Appendix. Therapeutic algorithm for management of ESA dose and hemoglobin levels.

As for usual clinical practice, we suggest to treat patients with ESA by 2-3 epoetin alfa or beta (EPO) administrations at week or by darbepoetin alfa administration once a week, as follows:

- High ESA dose arm. Patients randomized to ESA high dose will receive:
  - EPO alfa 6000 IU for 3/week or
  - EPO beta 6000 IU for 3/week or
  - darbepoetin 90 (40+50 or 60+30 or 80+10) micrograms/once a week
- Low ESA dose arm. Patients randomized to low dose will receive:
  - EPO alfa 2000 UI for 3/week or
  - EPO beta 2000 UI for 3/week or
  - darbepoetin 20 micrograms/ once a week

If hemoglobin values, detected in two consecutive blood samples, are below 9.5 g/dL or above 12.5 g/dL, ESA dose should be gradually changed by 25%, according to the current clinical practice guidelines. A therapeutic algorithm for management of these changes is below.

**Lowering ESA dose in patients randomized to fixed high dose (epoetin alfa or beta 18000 IU/week or darbepoetin 90 micrograms/week) for persisting hemoglobin levels > 12.5 g/dL**

If patients randomized to fixed high ESA dose have hemoglobin levels >12.5 g/dL, detected after two consecutive assessments, ESA dose should be decreased, as follows:

- EPO alfa or beta 14000 IU/week or
- Darbepoetin 70 micrograms/week

If at next assessment hemoglobin level is still >12.5 g/dL and this value is confirmed by a second blood sample, ESA dose should be decreased as follows:

- EPO alfa or beta 10000 IU/week or
- Darbepoetin 50 micrograms/ week

If at next assessment hemoglobin level persists >12.5 g/dL and this value is confirmed by a second blood sample, ESA dose should be decreased as follows:

- EPO alfa or beta 8000 IU/week or
- Darbepoetin 40 micrograms/week

If at next assessment hemoglobin level persists >12.5 g/dL and this value is confirmed by a second blood sample, ESA dose should be decreased as follows:

- EPO alfa or beta 6000 IU/week or
- Darbepoetin 30 micrograms/week

If at next assessment hemoglobin level persists >12.5 g/dL and this value is confirmed by a second blood sample, ESA dose should be decreased as follows:

- EPO alfa or beta 4000 IU/week or
- Darbepoetin 20 micrograms/ week

For persisting hemoglobin level >12.5 g/dL (two consecutive assessments), detected during follow up visits (at 1, 2, 3, 6, 12, 18, 24, 30, 36, 42, 48 months) or during routine monitoring provided by standard dialysis clinical practice, ESA treatment should be interrupted until hemoglobin levels return to < 12.5 g/dL.

**Increasing ESA dose in patients randomized to fixed high dose (epoetin alfa or beta 18000 IU/week or darbepoetin 90 micrograms/week) for persisting hemoglobin levels <9.5 g/dL**

If patients randomized to fixed high ESA dose have hemoglobin levels <9.5 g/dL, detected after two consecutive assessments, ESA dose should be increased, as follows:

- EPO alfa or beta 22000 IU/week or
- Darbepoetin 110 micrograms/week

If at next assessment hemoglobin level is <9.5 g/dL and this value is confirmed by a second blood sample, ESA dose should be increased as follows:

- EPO alfa or beta 28000 UI/ week or
- Darbepoetin 140 micrograms/ week

If at next assessment hemoglobin level persists <9.5 g/dL and this value is confirmed by a second blood sample, ESA dose should be increased as follows:

- EPO alfa or beta 35000 UI/ week or
- Darbepoetin 175 micrograms/ week

For persisting hemoglobin levels <9.5 g/dL (two consecutive assessments), detected during follow up visits (at 1, 2, 3, 6, 12, 18, 24, 30, 36, 42, 48 months) or during routine monitoring provided by standard dialysis clinical practice, ESA dose should be of 35000 EPO alfa or darbepoetin 175 micrograms/week.

**Lowering ESA dose in patients randomized to fixed low dose (epoetin alfa or beta 4000 IU/week or darbepoetin 20 micrograms/week) for persisting hemoglobin levels > 12.5 g/dL**

If patients randomized to fixed low ESA dose have hemoglobin levels >12.5 g/dL, detected after two consecutive assessments, ESA dose should be decreased, as follows:

- EPO alfa or beta 3000 IU/week
- Darbepoetin 15 micrograms/week

If at next assessment hemoglobin level persists <9,5 g/dL and this value is confirmed by a second blood sample, ESA dose should be decreased as follows:

- EPO alfa o beta 2000 IU/week
- Darbepoetin 10 micrograms/ week

For persisting hemoglobin level >12.5 g/dL (two consecutive assessments), detected during follow up visits (at 1, 2, 3, 6, 12, 18, 24, 30, 36, 42, 48 months) or during routine monitoring provided by standard dialysis clinical practice, ESA treatment should be interrupted until hemoglobin levels return to < 12.5 g/dL.

**Increasing ESA dose in patients randomized to low fixed ESA dose (epoetin alfa or beta 4000 IU/week or darbepoetin 20 micrograms/week) for persisting hemoglobin levels <9.5 g/dL**

If patients randomized to fixed low ESA dose have hemoglobin levels <9.5 g/dL, detected after two consecutive assessments, ESA dose should be decreased, as follows:

- EPO alfa or beta 5000 IU/week or
- Darbepoetin 25 micrograms/week

If at next assessment hemoglobin level is still <9.5 g/dL and this value is confirmed by a second blood sample, ESA dose should be increased as follows:

- EPO alfa or beta 6000 IU/week or
- Darbepoetin 30 micrograms/ week

If at next assessment hemoglobin level persists <9.5 g/dL and this value is confirmed by a second blood sample, ESA dose should be increased as follows:

- EPO alfa or beta 8000 IU/week or
- Darbepoetin 40 micrograms/ week

For persisting hemoglobin level <9.5 g/dL (two consecutive assessments), detected during follow up visits (at 1, 2, 3, 6, 12, 18, 24, 30, 36, 42, 48 months) or during routine monitoring provided by standard dialysis clinical practice, ESA dose could be increased until hemoglobin levels become >9.5 g/dL.
