# Supplementary material for: Low versus high dose erythropoiesis-stimulating agents in hemodialysis patients with anemia: A randomized clinical trial
Source: PLoS One. 2017 Mar 1;12(3):e0172735. doi: 10.1371/journal.pone.0172735 (PMC5332066; doi:10.1371/journal.pone.0172735)
Supplement: S5 Appendix — (DOCX) [file pone.0172735.s005.docx]

## S5 Appendix. Non-randomized standards of care.

During follow up, patients received, in a non-randomized fashion, additional co-interventions as per their usual attending physician’s practice to achieve and maintain the following standard dialysis clinical performance measures:

- Kt/V_urea_ ≥ 1.3
- Serum Albumin >35 g/L
- nPCR>1.0 g/kg/day
- Ferritin 200-500 μg/L
- TSAT 30-40%
- Calcium 8.4-9.5 mg/dL (2.1–2.4 mmol/L)
- Phosphorus 3.5–5.5 mg/dL (1.1–1.8 mmol/L)
- PTH: 150–300 pg/mL (16.5–33 pmol/l)
- Systolic blood pressure (predialysis) ≤140 mmHg
- Diastolic blood pressure (predialysis) ≤90 mmHg
- Average inter-dialytic weight gain for month ≤4% of dry weight
- Dialysis blood flow rate >300 mL/min
- Total Cholesterol <175 mg/dl (4.5 mmol/l)
- LDL Cholesterol <100 mg/dl (2.59 mmol/l)
- HDL Cholesterol ≥40 mg/dl (1.0 mmol/l)
- Triglycerides <180 mg/dl (2.0 mmol/l)
