# Supplementary material for: Low versus high dose erythropoiesis-stimulating agents in hemodialysis patients with anemia: A randomized clinical trial
Source: PLoS One. 2017 Mar 1;12(3):e0172735. doi: 10.1371/journal.pone.0172735 (PMC5332066; doi:10.1371/journal.pone.0172735)
Supplement: S6 Appendix — (DOCX) [file pone.0172735.s006.docx]

## S6 Appendix. Definitions of study endpoints.

1. **All-cause mortality**: death due to any cause.
2. **Cardiovascular death:** any death which happens within 28 days of the onset of acute myocardial infarction or stroke, in the absence of concurrent causes (e.g. infection, trauma etc.), all sudden deaths, all deaths due to congestive heart failure and all deaths which are coded as cardiovascular. The absence of any evidence of non-cardiovascular cause is sufficient to define death as cardiovascular. If patient affected by a severe non cardiovascular disease dies due to a cardiovascular event, such event will be considered as the cause of death. In all other cases, the death will be considered as “death due to other causes”.
3. **Non-fatal myocardial infarction:** the presence of two or more of the following:
   1. typical ischemic chest pain, pulmonary edema, syncope or shock
   2. development of pathological Q-waves and/or appearance or disappearance of localized ST-elevation followed by T-wave inversion in two or more of twelve standard electrocardiograph leads
   3. raised concentration of serum markers consistent with myocardial damage (e.g. rise and fall of CK >2 x ULN, elevated CK-MB, elevated troponin).

“Silent” myocardial infarctions are not to be included.

1. **Non-fatal stroke:** rapid (or uncertain) onset of focal or global neurological deficit lasting more than 24 hours or leading to death. For any stroke reported, information will be sought for review of the likely etiology (ischemic or hemorrhagic), on the base of clinical and instrumental data (TAC, RM).
2. **Acute coronary syndrome (hospitalization for):** hospitalizations due to ischemic episodes at rest lasting more than 5 minutes and when at least one of the following is present:
   1. ECG evidence of myocardial ischemia with ST>0.5 mm deviation;
   2. persisting deviation of ST segments (>30 minutes) <0.5 mm;
   3. increased CPK or CPK-MB levels above normal range;
   4. increased troponin T and I levels.
3. **Transient ischemic attack (hospitalization for):** onset of focal or monocular neurological deficit (amaurosis fugax) lasting less than 24 hours and probably due to vascular cause.
4. **Non-planned coronary revascularization procedures** **(hospitalization for):** hospital treatment consisting of PCI-Percutaneous Transluminal Coronary Intervention (e.g. angioplasty, stenting, atherectomy, laser ablations) or CABG-Coronary Artery Bypass Graft within 14 days before another acute event or signs/symptoms worsening of acute coronary syndrome.
5. **Peripheral revascularization procedures (hospitalization for):** all interventions for peripheral revascularization following peripheral vasculopathy.
6. **Vascular access thrombosis:** total or partial occlusion of vascular access such as it is no longer useable for hemodialysis treatment and it requires surgical intervention for reviewing or reuse
7. **Hypertension:** hypertensive crisis with encephalopathy-like symptoms and tonic-clonic seizures that require immediate medical care (as reported by leaflet) and / or increases in blood pressure for which the physician investigator carries out the acute administration of medication.
8. **Seizures:** tonic-clonic seizures

Other definitions of fatal events

**Myocardial infarction:**

1. Death that occurs within 7 days after a documented myocardial infarction in which there is no conclusive evidence of another cause of death
2. Autopsy evidence of a recent infarct with no other conclusive evidence of another cause of death
3. Suggestive criteria for an infarct but does not meet the strict definition of a myocardial infarction:
   1. ECG changes indicative of an acute injury
   2. Abnormal markers levels without evolutional changes (death before next assessments)
   3. Other important abnormalities

**Fatal stroke:** death which occurs within 7 days after a documented stroke. Fatal stroke may have occurred before 24 hours of the onset of symptoms (neurological deficit).
