# Supplementary material for: Low versus high dose erythropoiesis-stimulating agents in hemodialysis patients with anemia: A randomized clinical trial
Source: PLoS One. 2017 Mar 1;12(3):e0172735. doi: 10.1371/journal.pone.0172735 (PMC5332066; doi:10.1371/journal.pone.0172735)
Supplement: S1 File — (PDF) [file pone.0172735.s007.pdf]

Effects of the dose of erythropoiesis stimulating agents on cardiovascular events, quality of life in hemodialysis patients: The Clinical Evaluation of the DOSe of Erythropoietin (C.E. DOSE) trial protocol.

This study has been approved and funded by the Italian Agency on drugs within the 2006 funding plan for independent research on drugs.

Study protocol N.FARM6X822T

Table of Contents

Study administration and investigators....4
Principal investigator....4
Steering Committee....4
Trial coordinating center....4
Clinical Research Management and Monitoring....4
List of abbreviations and definition of terms....5
Synopsis....6
Introduction....7
References....8
Study objectives....9
Primary end point....9
Secondary end points....9
  Clinical efficacy....9
  Safety....10
  Quality of life....10
  Clinical feasibility....10
Methods....11
Study design....11
Study population....12
  Inclusion criteria....12
  Exclusion criteria....12
Interventions....12
  Methods for assigning subjects to treatment groups....12
  Dosage regimen....12
  Study blinding....13
  Drug accountability....13
  Treatment compliance....13
Prior and concomitant medication....13
Schedule of measurement....13
  Randomization visit (0 month)....13
  Follow up visit (months 1, 2, 3, 6, 12)....13
Assessment of treatment efficacy....16

Quality of life assessment ... 16
Safety assessment ... 16
Adverse events ... 16
Serious adverse events ... 16
Data Quality Assurance ... 17
Data management procedures ... 18
Sample size estimates ... 19
Statistical analyses ... 19
Interim analyses ... 21
Ethics ... 22
Independent ethics committees (IECS) ... 22
Ethical conduct of the study ... 22
Subject / patient informed consent ... 22
Amendments to the original protocol ... 30
Appendix 1: Definitions of study endpoints ... 24
Appendix 2: Non-randomized standards of care ... 29
Appendix 3: Therapeutic algorithm for management of ESA dose ... 26

Study administration and investigators

Principal investigator

Giovanni Strippoli - Fondazione Mario Negri Sud, Santa Maria Imbaro (Chieti), Italy

Steering Committee

Strippoli Giovanni FM, Fondazione Mario Negri Sud, Santa Maria Imbaro (Chieti), Italy

Craig Jonathan C, School of Public Health University of Sydney, Australia

Strippoli Paolo, Reparto di Nefrologia e Dialisi, Presidio Ospedaliero " A. Perrino", Brindisi, Italia

Di Giulio Salvatore, A.O. San Camillo Forlanini, Roma, Italia

Nicolucci Antonio, Fondazione Mario Negri Sud, Santa Maria Imbaro (Chieti), Italy

Pellegrini Fabio, Fondazione Mario Negri Sud, Santa Maria Imbaro (Chieti), Italy

Procaccini Deni A, Divisione di Nefrologia, Ospedali Riuniti, Foggia, Italia

Santoro Antonio, U.O di Nefrologia e Dialisi, Policlinico S. Orsola – Malpighi, Bologna, Italia

Tognoni Giovanni, Fondazione Mario Negri Sud, Santa Maria Imbaro (Chieti), Italy

Triolo Giorgio, U.O.A. Nefrologia e Dialisi Azienda Ospedaliera, C.T.O./C.R.F./ M. Adelaide, Torino, Italia

Trial coordinating center

Fondazione Mario Negri Sud, Santa Maria Imbaro (Chieti), Italy

Valeria Saglimbene (trial coordinator); Barbara Di Nardo, Riccarda Memmo, Cristina Di Biase, Giuliana Di Nardo and Rosalia Di Lallo (database design, data management)

Clinical Research Management and Monitoring

Miriam Valentini, Daniela D'Alonzo and Celeste Pirozzoli, Department of Clinical Pharmacology and Epidemiology, Unit of Clinical Research Management and Monitoring, Fondazione Mario Negri Sud, Santa Maria Imbaro, Italy.

List of abbreviations and definition of terms

CKD	Chronic Kidney Disease
CRF	Case Report Form
CRP	C Reactive Protein
DCF	Data Clarification Form
ESA	Erythropoiesis stimulating agents
ESKD	End Stage Kidney Disease
FMNS	Fondazione Mario Negri Sud
GFR	Glomerular Filtration Rate
GSP	Good Clinical Practice
Hb	Hemoglobin
IEC	Independent Ethic Committee
PROBE	Prospective Randomized Open Blinded End-Point
QoL	Quality of life
QT	Quality Team
RCT	Randomized Controlled Trial
SOP	Standard Operating Procedure

Synopsis

**Name of finished product**	Any erythropoiesis stimulating agents commercially available
**Name of active ingredient(s)**	Erythropoietin alfa, beta, or equivalent doses of any other commercially available erythropoiesis stimulating agent
**Title**	Effects of the dose of erythropoiesis stimulating agents on cardiovascular events and quality of life in hemodialysis patients: The Clinical Evaluation of the DOSe of Erythropoietin (C.E. DOSE) trial protocol
**Name of principal investigator**	Gianni Tognoni
**Objectives of study**	To assess the comparative efficacy of two fixed ESA doses (low versus high) on biomarkers levels (iron and inflammation indicators) and a composite of major cardiovascular events (fatal and non-fatal) and hospitalizations for cardiovascular causes. The effect on quality of life and feasibility indicators will be also assessed
**Methodology**	This is a phase III, randomized, comparative, pragmatic trial with prospective randomized open blinded endpoint (PROBE) design. Patients with ESKD, treated with hemodialysis, and requiring ESA therapy (who was already receiving ESA, as well as any patient in which the managing physician would initiate ESA treatment) will be involved and followed for 1 year according to scheduled visits (at 0, 1, 2, 3, 6, and 12 months after randomization)
**Number of subjects**	900 planned patients
**Diagnosis and criteria for inclusion**	Patients with ESKD and anemia, receiving hemodialysis as renal replacement therapy
**Experimental drug, dose, route of administration**	18000 IU/week iv of erythropoietin alfa, beta, or equivalent doses of any other commercially available erythropoiesis stimulating agent
**Duration of treatment**	1 year
**Reference therapy**	4000 IU/week iv of erythropoietin alfa, beta, or equivalent doses of any other commercially available agent
**Criteria for evaluation of efficacy and safety**	Criteria for evaluation of efficacy and safety have been establish respectively by anindependent blinded Endpoint Committee and an independent Data and Safety Monitoring Committee
**Statistical methodology**	Intention to treat analysis<br>Generalized linear mixed model<br>Cox-proportional hazards modelling

Introduction

Anemia is highly prevalent in people with chronic kidney disease (CKD). Around 5% of patients with stage 1 and 2 CKD [normal Glomerular Filtration Rate (GFR) or ≥ 90mL/min/1,73m2 and GFR 60 to 89mL/min/1,73 m2 according to Kidney Disease Outcomes Quality Initiative (K-DOQI)), 2-10% with stage 3 CKD (GFR 30 to 59mL/min/ 1,73 m2) and almost all patients with stages 4-5 CKD (ESKD) are affected (1).

Observational studies suggest that, compared to patients with CKD whose hemoglobin (Hb) levels are on average 11 g/dL, CKD patients with Hb levels <11 g/dL experience a 20-70% higher risk of death and a 20-40% higher risk of hospitalization (2); CKD patients with Hb levels >12 g/dL have a 15-20% lower risk of hospitalization with no survival advantage (3). However these studies, due to their observational design, can only establish an association between Hb and survival, and do not demonstrate a causal relationship between Hb levels and risk of death or hospitalizations.

Randomized Controlled Trial (RCTs) have consistently shown that Hb targets ≥ 13.0 g/dL achieved with Erythropoiesis Stimulating Agents (ESA) cause an increase in adverse vascular events and mortality compared to Hb levels of 10-12 g/dL achieved with the same agents or no treatment (4-6). Two meta-analysis of RCTs confirm these data (7, 8). In addition, RCTs, both individually and in their pooled analysis, find an improvement in quality of life (QoL) with progressively higher Hb target levels.

Based upon this increased vascular risk, Hb target levels > 13.0 g/dL are discouraged, notwithstanding the benefits on QoL. The main agencies of drugs, including Italian Agency on drugs and Food and Drug Administration recommend to use the lower ESA dose needed to achieve and maintain Hb values between 10.0-12.0 g/dL.

The mechanism by which targeting higher Hb levels causes an increase in the risk of death and vascular events remains uncertain. It is possible that the ESA dose required to achieve and maintain higher Hb targets is directly linked with adverse events (instead of the Hb levels achieved themself),most particularly in patients who are resistant to the actions of administered ESA (9, 10).

There are no RCTs that formally test this hypothesis, with its related mechanisms, and the criteria to define “ESA resistance” or “ESA hypersensitivity” remain unclear.

In order to establish the optimal therapeutic strategy for the management of anemia in ESKD, the Clinical Evaluation of the DOSe of Erythropoietin (C.E. DOSE) trial aims to answer the following open questions:

- Which mechanisms make some patients resistant to the action of ESA leading to the administration of higher-potentially toxic-doses;
- Which ESA dose decreases the risk of mortality and adverse vascular events related to anemia;
- Which ESA dose improves the patient sense of well-being, expressed as QoL.

We test two therapeutic strategies that are two fixed ESA doses. The first is based on the prescription of a minimum ESA dose (4000 IU per week) and the second is based on the administration of a maximum ESA dose (18000 IU per week), independent of the Hb target level which is achieved. Both strategies include a rescue mechanism for dose tapering when Hb levels fall outside the range of 9,5 to 12,5 g/dL.

References

1. Hsu CY, McCulloch CE, Curhan GC: Epidemiology of anemia associated with chronic renal insufficiency among adults in the United States: results from the Third National Health and Nutrition Examination Survey. *J Am Soc Nephrol* 2002, 13:504-510.
2. Collins AJ, Li S, St Peter W, Ebben J, Roberts T, Ma JZ, Manning W: Death, hospitalization, and economic associations among incident hemodialysis patients with hematocrit values of 36 to 39%. *J Am Soc Nephrol* 2001, 12:2465-2473.
3. Collins AJ, Ma JZ, Xia A, Ebben J: Trends in anemia treatment with erythropoietin usage and patient outcomes. *Am J Kidney Dis* 1998, 32(6 Suppl 4):133-141.
4. Besarab A, Bolton WK, Browne JK, Egrie JC, Nissenson AR, Okamoto DM, Schwab SJ, Goodkin DA: The effects of normal as compared with low hematocrit values in patients with cardiac disease who are receiving hemodialysis and epoetin. N Engl J Med 1998, 339(Suppl 9):584-590.
5. Singh AK, Szczech L, Tang KL, Barnhart H, Sapp S, Wolfson M, Reddan D; CHOIR Investigators: Correction of anemia with epoetin alfa in chronic kidney disease. N Engl J Med 2006, 355:2085-2098.
6. Drüeke TB, Locatelli F, Clyne N, Eckardt KU, Macdougall IC, Tsakiris D, Burger HU, Scherhag A; CREATE Investigators: Normalization of hemoglobin level in patients with chronic kidney disease and anemia. N Engl J Med 2006, 355(Suppl 20):2071-2084.
7. Strippoli GF, Craig JC, Manno C, Schena FP. Hemoglobin targets for the anemia of chronic kidney disease: a meta-analysis of randomized, controlled trials. J Am Soc Nephrol 2004,15:3154-65.
8. Phrommintikul A, Haas SJ, Elsik M, Krum H. Mortality and target haemoglobin concentrations in anaemic patients with chronic kidney disease treated with erythropoietin: a meta-analysis. Lancet 2007, 369:381-8.
9. Szczech LA, Barnhart HX, Inrig JK, Reddan DN, Sapp S, Califf RM, Patel UD and SinghAK: Secondary analysis of the CHOIR trial epoetin alfa dose and achieved haemoglobin outcomes. Kid Int 2008, 79:791-798.
10. Zhang Y, Thamer M, Stefanik K, Kaufman J, Cotter DJ: Epoietin requirements predict mortality in hemodialysis patients. Am J Kidney Dis 2004, 44:866-876.

Study objectives

The study aims to:

- To investigate predictors of individual variability in response to ESA treatment, when looking specifically at the dose effect on biomarker levels, such iron and inflammation status indicators;
- To generate evidence based knowledge that allows to guide and monitoring ESA therapy starting from lower doses;
- To evaluate the comparative efficacy of two fixed ESA doses (low versus high) on clinical outcomes (death, vascular events, safety) and QoL.

Primary end point

The two therapeutic strategies (fixed low dose versus fixed high dose) are compared on the basis of the following outcomes, considered as potential biomarkers of the ESA resistance/hypersensitivity profile:

➔ End of treatment mean differences between arms (high dose versus low dose) of:

- Serum ferritin;
- Serum transferrin;
- TSAT (%);
- Serum albumin;
- C Reactive Protein (CRP);
- Mean ESA dose.

Secondary end points

The two therapeutic strategies (fixed low dose versus fixed high dose) are compared also by reviewing the following secondary endpoints:

Clinical efficacy

- All-cause mortality;
- Fatal stroke;
- Non-fatal stroke;
- Fatal myocardial infarction;
- Non-fatal myocardial infarction;

- Hospitalization for cardiovascular causes (acute coronary syndrome, transient ischemic attack, non-planned coronary revascularization procedures and peripheral revascularization procedures);
- The composite end point of all-cause mortality, fatal and non-fatal stroke, fatal and non-fatal myocardial infarction, hospitalization for cardiovascular causes.

Safety

- Vascular access thrombosis;
- Seizures;
- Hypertension.

Quality of life

- Assessed by administration of the self-administration KDQOL-SFTM1.3 questionnaire.

Clinical feasibility

- Average variation of allocated ESA dose (IU/week for erythropoietins or microgram/week for darbepoetin) in the two arms;
- Average ESA dose variation based on weight and body mass index;
- Number of patients in each arm who maintained stable Hb levels between 10.0 and 12 g/dL, without need for >50% change in the allocated dose of ESA;
- Number of patients requiring one or more blood transfusion;
- Number of ESA dose variations from the randomization time to the time of Hb level stabilization (between 10.0-12.0 g/dL);
- Time from randomization to the first ESA dose variation;
- Time from randomization to Hb level stabilization (between 10.0-12.0 g/dL).

Definition of efficacy and safety end points is reported in ***Appendix 1***.

Methods

Study design

This is a pragmatic, multicenter, randomized controlled trial (**Figure 1**) based on the intention-to-treat principle and the Prospective Randomized Open Blinded End-Point (PROBE) technique. Eligible patients will be randomized (1:1) to ESA 4000 IU/week intravenously versus 18000 IU/week intravenously of erythropoietin alfa, beta or equivalent doses of any other commercially available agent. The steering committee has the task of planning and coordinating the study, conducting interim analyses (when needed), interpreting data, reviewing and approving the manuscripts for publication. During follow up, patients will receive (in a non-randomized fashion) additional co-interventions (e.g. iron, lipid lowering agents, bone disease agents, antihypertensive agents, etc.) as per their usual attending physician's practice to achieve and maintain standard dialysis clinical performance measures, relating to key CKD-related comorbidities.

Figure 1. Flow chart describing selection, randomization and follow-up process

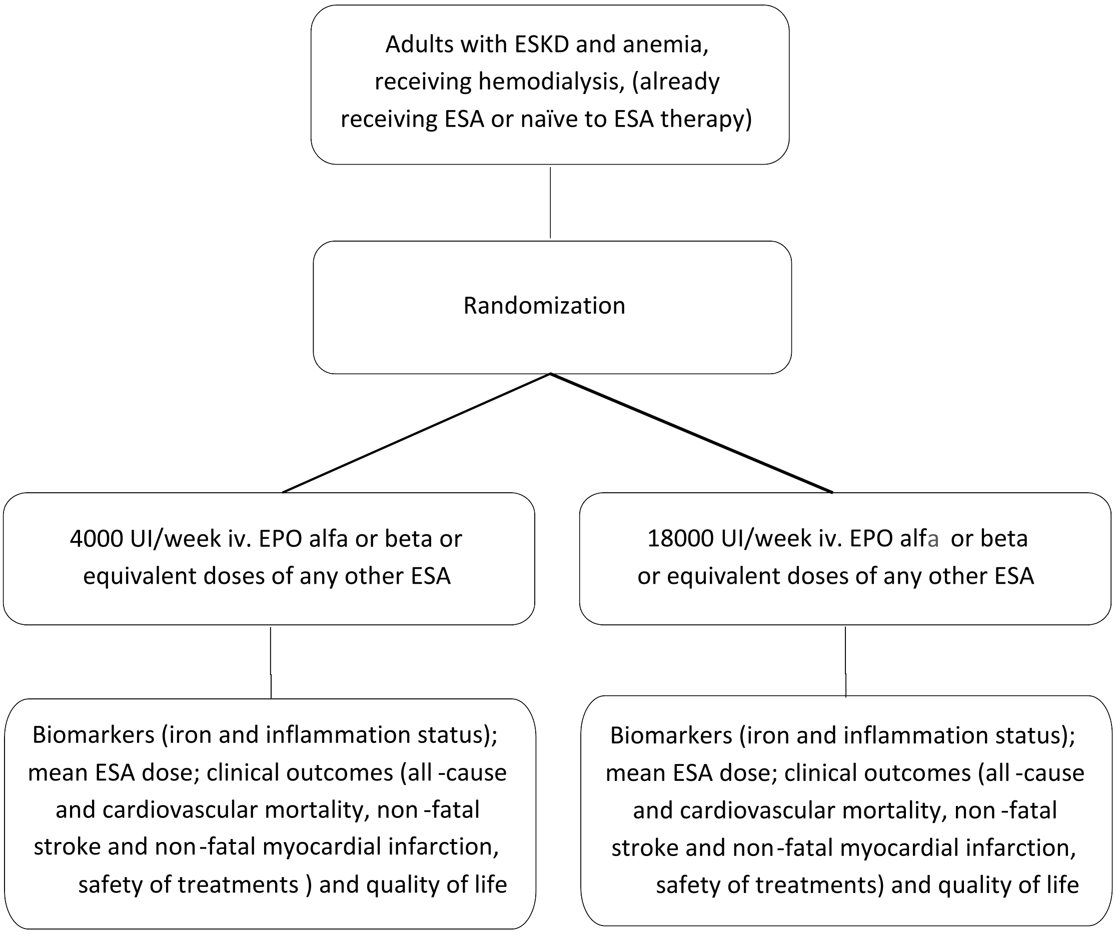

Study population

Patients >18 years aged old who fulfil the following criteria will be included:

Inclusion criteria

1) Presence of anemia related to ESKD. Any patient who is already receiving ESA is eligible, as well as any patient in whom the managing physician would initiate ESA treatment;
2) Renal replacement therapy with hemodialysis (bicarbonate dialysis, hemofiltration, hemodiafiltration, on-line hemodiafiltration, or acetate-free biofiltration).

Exclusion criteria

1) Contraindication to ESA treatment;
2) Patients will be excluded if they have a Hb>10 g/dL and are not currently receiving ESA treatment.

Interventions

Patients will be randomized to two fixed doses of ESA, administered intravenously, and without a washout period: a minimum ESA dose (4000 I/U per week) and a maximum ESA dose (18000 I/U per week), independently on the Hb target level achieved. The physician will choose the type of ESA: 4000 IU/week iv versus 18000 IU/week iv of erythropoietin alfa, beta or equivalent doses of any other commercially available agent.

Methods for assigning subjects to treatment groups

Patients will be randomly assigned to low dose ESA or high dose ESA with randomization stratified by dialysis clinic and in randomly permuted blocks of six. The random sequence for the allocation program will be created in FileMaker Pro 10 and allocation will be concealed to researchers by using remote, central assignment of treatment via telephone contact with masked researchers at the central trial coordination unit.

Dosage regimen

Although this is a fixed dose trial, after allocation to treatment, a safety mechanism will be in place to ensure that Hb values do not drop below 9.5 g/dL or do not exceed 12.5 g/dL, given existing evidence of potential harm outside this therapeutic range. An Hb value above or below this range will trigger an ESA dose change. The prescribed ESA dose will be gradually increased or decreased by 25% until Hb values return to between 9.5 and 12.5 g/dL. The therapeutic algorithm for management of ESA dose and hemoglobin levels is reported in ***Appendix 2***.

Study blinding
A prospective, randomized, open, blinded endpoint evaluation (PROBE) design will be adopted. Participants and investigators will be not masked to group allocation, but according to the PROBE design, an independent End-Point Committee of medical specialists within the disease of interest will be established. These physicians (cardiologists and nephrologists) will be unaware of the allocated treatment and will review all available documents (including charts, death certificates etc.) to provide a blinded adjudication of all outcomes.

Drug accountability
Study drugs will be prescribed by the investigator.

Treatment compliance
Patients received the study interventions during the routine dialysis treatment. Investigators will be responsible for treatment compliance. Any ESA dose change established by the investigator will be reported and motivated in an appropriate section of the Case Report Form (CRF).

Prior and concomitant medication

During follow up, patients will receive (in a non-randomized fashion) additional co-interventions (e.g. iron, lipid lowering agents, bone disease agents, antihypertensive agents, etc.) as per their usual attending physician’s practice to achieve and maintain standard dialysis clinical performance measures, related to key CKD-related comorbidities. Non-randomized standards of care are reported in ***Appendix 3***.

Schedule of measurement

Randomization visit (0 month)
After allocation to treatment, the following baseline data will be recorded in the appropriate CRF: social and demographic data; patients clinical history; blood count; lipid profile; glycemic profile; assessment of liver and kidney functions; systolic and diastolic blood pressure; heart rate; assessment of concomitant illness. Further, detailed information on dialysis, intradialysis and/or home therapy related to ESKD and concomitant medications will be reported. Finally, QoL will be assessed by self-administration of the KDQOL-SFTM1.3 questionnaire.

Follow up visit (months 1, 2, 3, 6, 12)
After randomization visit, patients will be scheduled to attend follow-up visits at 1, 2 , 3, 6 and 12 months. At each visit, a follow-up CRF will be completed; the occurrence of all clinical end-point will

be ascertained and minimum clinical workup and laboratory indicators (Hb levels, details on dialysis treatment, intradialysis and/or hometherapyrelated to ESKD,concomitant medication) will be recorded. At 6 and 12 months visits, QoL will be assessed by self-administration of the KDQOL-SF^{TM}1.3 questionnaire. Additional visits could be performed and additional information could be registered in special CRF. When a patient withdraw from the study (for example he moved on and didn't come back to dialysis facility), a special report form will be completed. **Table 1** shows details of visit timelines and detected parameters.

Table 1. Overview of visits and measurements

Detected Indicators	Visit timelines					
	Randomization time* 0	Month 1#	Month 2#	Month 3#	Month 6	Month 12
Consent	X					
Clinical history	X					
Hb	X	X	X	X	X	X
Htc, MCV, MCH, MCHC, platelet		X	X	X		
Red blood cell count, PDW,Pct, MPV,RDW	X				X	X
Lipid and glucose profile, liver and renal function	X				X	X
Blood pressure and heart rate	X	X	X	X	X	X
Safety and efficacy assessment		X	X	X	X	X
Detailed information on intradialysis and/or home therapy related to ESKD	X	X	X	X	X	X
Detailed information on dialysis (nPCR, Kt/V, dry weight, interdialytic weight gain, dialysis blood flow rate, duration and type of dialysis, type of filter)	X	X	X	X	X	X
KDQOL-SFTM1.3	X				X	X

*At randomization time Hb, Kt/V and dry weight relative to months 1 and 2 before randomization must be reported

#Hb levels must be also detected at 2, 6 and 10 weeks thereafter randomization and recorded at visits at 1, 2 and 3 months respectively

Assessment of treatment efficacy

The End-Point Committee, unaware of allocated treatment, will provide a blinded adjudication of all outcomes on the basis of anonymous documents provided by the investigators. The efficacy of the experimental interventions will be compared reviewing the primary and secondary outcomes previously described (Paragraph "Study Objectives").

Quality of life assessment

Quality of life (QoL) will be assessed at baseline, six and 12 months thereafter by administration of the KDQOL-SFTM[1.3] questionnaire. This self-administered tool includes 2 QoL instruments, the SF36 and the KDQOL, which are, respectively, a generic and CKD-specific QoL measure. The KDQOL-SFTM[1.3] questionnaire consists of 18 scales: 8 from the SF-36 questionnaire (13, 14) (physical function, role limitations caused by physical health problems, role limitations caused by emotional health problems, bodily pain, general health perception, vitality, social activities, and mental health) and 10 from the KDQOL questionnaire (15) (symptoms, burden of kidney disease, work status, cognitive function, quality of social interaction, sexual function, sleep, social support, patients satisfaction). Each scale is scored on a 0 to 100 possible range, with higher scores representing better quality of life.

Safety assessment

Adverse events

An adverse event is defined as any unfavourable sign, symptom or medical event, whether or not due to study treatments, that occurs from the first study related activity after the patient's signature of the informed consent. After the first study treatment consumption, worsening of pre-existing illness will be considered adverse events.

All details of adverse events, including adverse drug reactions, will be recorded on the Adverse Event Form, including: 1) description; 2) duration; 3) severity; 4) relationship to study drugs; 5) action taken to adverse event (treatments, diagnostic tests); 5) event resolution. All adverse events must be followed until they are resolved.

Serious adverse events

Serious adverse events are defined as those adverse events that results in any of the following:

- Death;
- A life threatening experience;
- Patient hospitalization or prolongation of existing hospitalization;
- A persistent or significant disability or incapacity;
- Important medical events in the opinion of the responsible investigator (i.e. any event that is not immediately life-threatening and does not result in death or hospitalization but which may jeopardize the participation to study or may require intervention to prevent one or the other outcomes listed above).

The physician will report all serious adverse events to the Coordinating Center within 24 hours after knowledge of the event. The Coordinating center will provide this information to Ministry of Health. If the patient dies, physician will alert the Ethic Committee and the Coordinating Center within 24 hours. All details of any serious adverse event, whether or not due to study drugs, will be described on the appropriate Serious Adverse Event Form. If the physician considers an adverse event due to study drugs, the "Single suspected adverse reaction reporting form" will be completed and provided to the responsible for pharm vigilance healthcare facility according to the current pharm vigilance rules (April 8, 2003, n.95).

Data Quality Assurance

A quality team specific for the study has been established at Fondazione Mario Negri Sud (FMNS). After the IEC approval, each center will receive an on-site initiation visit or a phone call by one of the study monitors. During this contact the study staff will receive information about study protocol, CRF, SOPs specific for the investigators, GCP.

During the study, the clinical monitor will visit and/or phoned the investigator center in order to assess completeness and accuracy of data, progress of enrolment and coherence with protocol and GCP. The investigator will cooperate during the on-site visits, by providing access to clinical data so that the consistency with data reported on CRF could be ascertained. The clinical monitor will ensure secrecy on all information. Standard monitor procedures will include: checking written informed consent, implementation of inclusion/exclusion criteria, adverse events documentation, recording of safety and efficacy data, random source data verification.

The investigator will fill the CRF (an original signed one and a copy) and report information according to the SOPs. After completing it, he/she will keep a copy of the CRF, along with the other study documentation (protocol, protocol amendments, etc.) in the Investigator's File, which will be kept in a safe place. The original one will be sent to the FMNS. Patient information will be provided on the CRF so that the subjects will be identified by a number only.

Investigators will store all the original records of patients, demographic and medical information and a copy of the signed informed consent from the patient. All information recorded on CRF will be traceable in the original clinical documentation contained in the patient case history. Essential documents must be filed by the investigators for at least 7 years after the end of the study. The Coordination Center will notify investigators and institutions when it is no longer necessary to store the material of the study. Investigators must accept the storage procedures by signing the protocol.

Data management procedures

All required information will be reported on CRF, in two copies. Clinical monitors will ascertain completeness and accuracy of collected data at random and will help investigator staff to review information, if bias or missing data are found. CRFs, originally signed by investigator, will be provided to the Quality Team by the Clinical Monitor or by the investigator.

The Quality Team will check and enter all data from any CRF into an appropriate database. The software used for the data input will be FileMaker. The study electronic files will be stored in a central server and the access will be regulated by identification and password. The backup of these files will take place daily.

Collection, validation and data entry will take place according to the specific SOPs. Information reported on CRF will be checked by the Quality Team and then entered into a validated database. Bias and missing data will be identified by an appropriate electronic system and by manual review of any CRF. In these occasions Data Clarification Forms (DCFs) will be generated and provided to the investigator center for resolving all queries. The signed DCF copy will be stored by the investigator center together with the respective CRF;

the originally signed one will be sent to the Quality Team, which will enter the answers to queries into the database. Finally, all the CRFs will be stored in a safe place.

Sample size estimates

In order to evaluate a “small effect size” (0.2), with alpha=0.05 and 1-beta=0.80, related to the primary end point of the difference between groups in serum ferritin or transferrin or TSAT (%) or PCR or albumin or mean ESA dose at the end of treatment, a sample size of 900 patients is needed (N=450 for each group).

In order to evaluate a “medium effect size” (0.5), with alpha=0.05 and 1-beta=0.80, related to the primary end point when looking at the subgroup of ESA resistant/hypersensitive patients only-assumed to be the 15% of the total cohort- a sample size of 150 subjects is needed (N=75 for each arm).

A sample size of 900 subjects allows determining a 30% reduction in the composite end point of all-cause mortality, fatal and non-fatal stroke, fatal and non-fatal myocardial infarction, hospitalization for cardiovascular causes in the experimental group compared to the control group, when assuming:

a) 35% annual incidence of this composite end-point;
b) 80% statistic power (alpha=of 0.05);
c) 30% (hazard ratio=0.85) relative risk reduction in the composite end-point;
d) 5% dropout rate.

Statistical analyses

All the efficacy analyses will be based on the intent to treat population, consisting of all randomized patients.

Demographic and clinical characteristics will be summarized by treatment arm. Continuous variables will be reported as mean ± standard deviation for normally distributed continuous variables, median and interquartile range for non-normally distributed variables. Categorical variables will be reported as frequency and percentage. Between-arms comparisons will be assessed using Mann-Whitney U-test for continuous variables and Pearson chi-square or Fisher exact test for the categorical ones.

Changes from randomization time to 1 year follow up in serum levels of ferritin, transferrin, albumin TSAT, CRP and ESA dose (primary end points) will be analysed using generalized linear mixed model with an auto-regressive correlation structure, with treatment groups and visits as fixed factors. Comparison of the two arms at each visit will be also reported using the appropriate contrasts.

For the secondary end-points (all-cause mortality, fatal and non-fatal stroke, fatal and non-fatal myocardial infarction, hospitalization for cardiovascular causes, vascular access thrombosis, seizures and hypertension), the time to the first event will be estimated using a Cox proportional hazards model. Additionally, the Kaplan-Meier method and comparisons between arms will be reported in terms of log-rank tests.

The treatments effect on QoL will be assessed comparing the two groups in terms of changes from randomization time to 1 year follow up in the physical function scale of the SF-36 questionnaire. We will conduct additional analyses to evaluate any QoL scale of the KDQOL-SFTM1.3 questionnaire.

All statistical analyses will be performed using SAS Statistical Package Release 9.3 (SAS Institute, Cary, NC, USA) on a Windows XP platform. All statistical tests were two-sided at alpha level of 0.05, and precision of the estimates referred to 95% confidence intervals (CIs).

We will repeat all the analyses considering the subgroup of ESA resistant/hypersensitive patients only. The response to ESA (ESA resistance/hypersensitive) of each patient should be assessed according to:

- Change (%) in Hb levels two weeks after randomization, when adjusting for the baseline ESA dose;
- Ratio between Hb levels achieved and ESA dose administered at the end of the study, when adjusting for the baseline ESA dose;
- Ratio between mean Hb levels achieved and mean ESA dose administered over the course of the study, when adjusting for the baseline ESA dose;
- Number of ESA dose variations from randomization time to the time of Hb level stabilization (two consecutive measures between 10.0-12.0 g/dL).

We will evaluate the treatments effect on clinical outcomes and QoL also based upon a series of potential effect modifiers (baseline information) including:

- Socio-demographic factors (gender, age, ethnicity, marital status, education, occupational status);
- Presence/absence of cardiovascular risk factors (diabetes mellitus, hypertension, smoking, family history of cardiovascular disease);
- Presence/absence of previous major cardiovascular event;
- Presence/absence of other major concomitant illness;
- Type of ESA administered;
- Average ESA dose administered during the study;
- Metabolic control (quartiles of total/LDL/HDL-cholesterol and HbA1c);
- Other cointerventions (iron, vitamin D or vitamin D analogues, phosphate binders, calcimimetics, antihypertensive agents, statins, anticoagulants);
- Baseline and end of treatment levels of calcium, phosphorus, iron, parathyroid hormone; Kt/V, dry weight, dialysis blood flow rate, interdialytic weight gain;
- Type of dialysis;
- Dialysis duration (minutes per dialysis session).

The Mantel-Haenszel procedure will be applied to test for the linearity of effects across subgroups, while the chi-square test will be applied to test for heterogeneity of effects among the subgroups.

Interim analyses

During the study, when needed, the Steering Committee will conduct efficacy and safety interim analyses in order to verify the study assumptions and the need for carrying out recruitment and/or follow up. The rules for early recruitment completion are:

- Converging hemoglobin levels and ESA doses between groups;
- Equivalent primary end points between groups.

Ethics

Independent ethics committees (IECS)

Study protocol, patient's information sheet and consent form will be approved by the Ethics Committees of all the participating centers. Every change to the study protocol will be performed as a substantial or a non-substantial amendment, according to the Italian law. Amendments to the original study protocol approved by Coordinating and local Ethics Committees are summarized in ***Appendix 4***.

Ethical conduct of the study

This study will be conducted according to the protocol, good clinical practice (GCP), current legislation and Standard Operating Procedures (SOPs) drawn up by Fondazione Mario Negri Sud. SOPs will be written according to the GCP as described in the following documents:

1. ICH Harmonized Tripartite Guidelines for Good Clinical Practice 1996.
2. Recommendations guiding Physicians in Biomedical Research involving Human Subjects, Helsinki (1964 amendments Tokyo 1975, Venezia 1983, Hong Kong 1989, Sommerset West 1996, Edinburgh 2000).
3. Legislative Decree n° 211 of June 24th2003 Transposition of Directive 2001/20/EC relating to the implementation of good clinical practice in the conduct of clinical trials on medicinal products for clinical use.
4. Ministerial Decree December 17th2004 Prescriptions and conditions of a general nature referring to the conduct of clinical trials of medicines with special reference to those designed to enhance clinical practice as an integral part of health and medical care.
5. Ministerial Decree of July 15th1997: "Transposition of guidelines of the European Union in good clinical practice for the conduction of clinical trials with medicines".

Principal investigators will accept procedures described above, and will declare that the study will be conducted according to GCP by signing the protocol.

Subject / patient informed consent

Patients will be enrolled after reading the information sheet and discussing the rationale and aims of the study with the site personnel. In obtaining and documenting informed

consent, the principal investigator will comply with the applicable regulatory requirement(s) and adhered to the ICH GCP guidelines and the requirements in the Declaration of Helsinki.

- Patients will express their willingness to participate in the study by signing the informed consent form, according to the GCP rules and the Italian law. A voluntary, signed and dated Informed consent form will be obtained from the subject prior to any study-related activity. The written informed consent will be signed and dated also by the site personnel who will conduct the informed consent procedure.

Appendix 1: Definitions of study endpoints

1. **All-cause mortality**: death due to any cause.
2. **Cardiovascular death:** any death which happens within 28 days of the onset of acute myocardial infarction or stroke, in the absence of concurrent causes (e.g. infection, trauma etc.), all sudden deaths, all deaths due to congestive heart failure and all deaths which are coded as cardiovascular. The absence of any evidence of non-cardiovascular cause is sufficient to define death as cardiovascular. If patient affected by a severe non cardiovascular disease dies due to a cardiovascular event, such event will be considered as the cause of death. In all other cases, the death will be considered as "death due to other causes".
3. **Fatal myocardial infarction:**
   - Death that occurs within 7 days after a documented myocardial infarction in which there is no conclusive evidence of another cause of death
   - Autopsy evidence of a recent infarct with no other conclusive evidence of another cause of death
   - Suggestive criteria for an infarct but does not meet the strict definition of a myocardial infarction:
     i. ECG changes indicative of an acute injury
     ii. Abnormal markers levels without evolutional changes (death before next assessments)
     iii. Other important abnormalities
4. **Non-fatal myocardial infarction:** the presence of two or more of the following:
   - Typical ischemic chest pain, pulmonary edema, syncope or shock;
   - Development of pathological Q-waves and/or appearance or disappearance of localized ST-elevation followed by T-wave inversion in two or more of twelve standard electrocardiograph leads;
   - Raised concentration of serum markers consistent with myocardial damage (e.g. rise and fall of CK >2 x ULN, elevated CK-MB, elevated troponin).

   "Silent" myocardial infarctions are not to be included.
5. **Fatal stroke:** death which occurs within 7 days after a documented stroke. Fatal stroke may have occurred before 24 hours of the onset of symptoms (neurological deficit).
6. **Non-fatal stroke:** rapid (or uncertain) onset of focal or global neurological deficit lasting more than 24 hours or leading to death. For any stroke reported, information will be sought for review of the likely etiology (ischemic or hemorrhagic), on the base of clinical and instrumental data (TAC, RM).

7. **Acute coronary syndrome (hospitalization for):** hospitalizations due to ischemic episodes at rest lasting more than 5 minutes and when at least one of the following is present:
    a. ECG evidence of myocardial ischemia with ST>0.5 mm deviation;
    b. Persisting deviation of ST segments (>30 minutes) <0.5 mm;
    c. Increased CPK or CPK-MB levels above normal range;
    d. Increased troponin T and I levels.
8. **Transient ischemic attack (hospitalization for):** onset of focal or monocular neurological deficit (amaurosis fugax) lasting less than 24 hours and probably due to vascular cause.
9. **Non-planned coronary revascularization procedures (hospitalization for):** hospital treatment consisting of PCI-Percutaneous Trans luminal Coronary Intervention (e.g. angioplasty, stenting, atherectomy, laser ablations) or CABG-Coronary Artery Bypass Graft within 14 days before another acute event or signs/symptoms worsening of acute coronary syndrome.
10. **Peripheral revascularization procedures (hospitalization for):** all interventions for peripheral revascularization following peripheral vasculopathy.
11. **Vascular access thrombosis:** total or partial occlusion of vascular access such as it is no longer useable for hemodialysis treatment and it requires surgical intervention for reviewing or reuse
12. **Hypertension:** hypertensive crisis with encephalopathy-like symptoms and tonic-clonic seizures that require immediate medical care (as reported by leaflet) and / or increases in blood pressure for which the physician investigator carries out the acute administration of medication.
13. **Seizures:** tonic-clonic seizures

Appendix 2: Therapeutic algorithm for management of ESA dose

As for usual clinical practice, we suggest to treat patients with ESA by 2-3 epoetin alfa or beta (EPO) administrations at week or by darbepoetin alfa administration once a week, as follows:

- High ESA dose arm. Patients randomized to ESA high dose will receive:
    - EPO alfa 6000 IU for 3/week or
    - EPO beta 6000 IU for 3/week or
    - darbepoetin 90 (40+50 or 60+30 or 80+10) micrograms/once a week
- Low ESA dose arm. Patients randomized to low dose will receive:
    - EPO alfa 2000 UI for 3/week or
    - EPO beta 2000 UI for 3/week or
    - darbepoetin 20 micrograms/ once a week

If hemoglobin values, detected in two consecutive blood samples, are below 9.5 g/dL or above 12.5 g/dL, ESA dose should be gradually changed by 25%, according to the current clinical practice guidelines. A therapeutic algorithm for management of these changes is below.

Lowering ESA dose in patients randomized to fixed high dose (epoetin alfa or beta 18000 IU/week or darbepoetin 90 micrograms/week) for persisting hemoglobin levels > 12.5 g/dL

If patients randomized to fixed high ESA dose have hemoglobin levels >12.5 g/dL, detected after two consecutive assessments, ESA dose should be decreased, as follows:

- EPO alfa or beta 14000 IU/week or
- Darbepoetin 70 micrograms/week

If at next assessment hemoglobin level is still >12.5 g/dL and this value is confirmed by a second blood sample, ESA dose should be decreased as follows:

- EPO alfa or beta 10000 IU/week or
- Darbepoetin 50 micrograms/ week

If at next assessment hemoglobin level persists >12.5 g/dL and this value is confirmed by a second blood sample, ESA dose should be decreased as follows:

- EPO alfa or beta 8000 IU/week or
- Darbepoetin 40 micrograms/week

If at next assessment hemoglobin level persists >12.5 g/dL and this value is confirmed by a second blood sample, ESA dose should be decreased as follows:

- EPO alfa or beta 6000 IU/week or
- Darbepoetin 30 micrograms/week

If at next assessment hemoglobin level persists >12.5 g/dL and this value is confirmed by a second blood sample, ESA dose should be decreased as follows:

- EPO alfa or beta 4000 IU/week or
- Darbepoetin 20 micrograms/ week

For persisting hemoglobin level >12.5 g/dL (two consecutive assessments), detected during follow up visits (at 1, 2, 3, 6, 12, 18, 24, 30, 36, 42, 48 months) or during routine monitoring provided by standard dialysis clinical practice, ESA treatment should be interrupted until hemoglobin levels return to < 12.5 g/dL.

Increasing ESA dose in patients randomized to fixed high dose (epoetin alfa or beta 18000 IU/week or darbepoetin 90 micrograms/week) for persisting hemoglobin levels <9.5 g/dL

If patients randomized to fixed high ESA dose have hemoglobin levels <9.5 g/dL, detected after two consecutive assessments, ESA dose should be increased, as follows:

- EPO alfa or beta 22000 IU/week or
- Darbepoetin 110 micrograms/week

If at next assessment hemoglobin level is <9.5 g/dL and this value is confirmed by a second blood sample, ESA dose should be increased as follows:

- EPO alfa or beta 28000 UI/ week or
- Darbepoetin 140 micrograms/ week

If at next assessment hemoglobin level persists <9.5 g/dL and this value is confirmed by a second blood sample, ESA dose should be increased as follows:

- EPO alfa or beta 35000 UI/ week or
- Darbepoetin 175 micrograms/ week

For persisting hemoglobin levels <9.5 g/dL (two consecutive assessments), detected during follow up visits (at 1, 2, 3, 6, 12, 18, 24, 30, 36, 42, 48 months) or during routine monitoring provided by standard dialysis clinical practice, ESA dose should be of 35000 EPO alfa or darbepoetin 175 micrograms/week.

Lowering ESA dose in patients randomized to fixed low dose (epoetin alfa or beta 4000 IU/week or darbepoetin 20 micrograms/week) for persisting hemoglobin levels > 12.5 g/dL

If patients randomized to fixed low ESA dose have hemoglobin levels >12.5 g/dL, detected after two consecutive assessments, ESA dose should be decreased, as follows:

- EPO alfa or beta 3000 IU/week
- Darbepoetin 15 micrograms/week

If at next assessment hemoglobin level persists <9,5 g/dL and this value is confirmed by a second blood sample, ESA dose should be decreased as follows:

- EPO alfa o beta 2000 IU/week
- Darbepoetin 10 micrograms/ week

For persisting hemoglobin level >12.5 g/dL (two consecutive assessments), detected during follow up visits (at 1, 2, 3, 6, 12, 18, 24, 30, 36, 42, 48 months) or during routine monitoring provided by standard dialysis clinical practice, ESA treatment should be interrupted until hemoglobin levels return to < 12.5 g/dL.

Increasing ESA dose in patients randomized to low fixed ESA dose (epoetin alfa or beta 4000 IU/week or darbepoetin 20 micrograms/week) for persisting hemoglobin levels <9.5 g/dL

If patients randomized to fixed low ESA dose have hemoglobin levels <9.5 g/dL, detected after two consecutive assessments, ESA dose should be decreased, as follows:

- EPO alfa or beta 5000 IU/week or
- Darbepoetin 25 micrograms/week

If at next assessment hemoglobin level is still <9.5 g/dL and this value is confirmed by a second blood sample, ESA dose should be increased as follows:

- EPO alfa or beta 6000 IU/week or
- Darbepoetin 30 micrograms/ week

If at next assessment hemoglobin level persists <9.5 g/dL and this value is confirmed by a second blood sample, ESA dose should be increased as follows:

- EPO alfa or beta 8000 IU/week or
- Darbepoetin 40 micrograms/ week

For persisting hemoglobin level <9.5 g/dL (two consecutive assessments), detected during follow up visits (at 1, 2, 3, 6, 12, 18, 24, 30, 36, 42, 48 months) or during routine monitoring provided by standard dialysis clinical practice, ESA dose could be increased until hemoglobin levels become >9.5 g/dL.

Appendix 3: Non-randomized standards of care

During follow up, patients received, in a non-randomized fashion, additional co-interventions as per their usual attending physician's practice to achieve and maintain the following standard dialysis clinical performance measures:

- $Kt/V_{urea} \geq 1.3$
- Serum Albumin >35 g/L
- nPCR>1.0 g/kg/day
- Ferritin 200-500 μg/L
- TSAT 30-40%
- Calcium 8.4-9.5 mg/dL (2.1–2.4 mmol/L)
- Phosphorus 3.5–5.5 mg/dL (1.1–1.8 mmol/L)
- PTH: 150–300 pg/mL (16.5–33 pmol/l)
- Systolic blood pressure (predialysis) ≤140 mmHg
- Diastolic blood pressure (predialysis) ≤90 mmHg
- Average inter-dialytic weight gain for month ≤4% of dry weight
- Dialysis blood flow rate >300 mL/min
- Total Cholesterol <175 mg/dl (4.5 mmol/l)
- LDL Cholesterol <100 mg/dl (2.59 mmol/l)
- HDL Cholesterol ≥40 mg/dl (1.0 mmol/l)
- Triglycerides <180 mg/dl (2.0 mmol/l)

Appendix 4: Amendments to the original protocol

Three amendments to the original study protocol were performed and approved by the IECs of the participating centers during the study.

Amendment n° 1

Amendment type: non–substantial

Amendment date: December 11th 2008

Amendment content:

- Correcting typographical errors;
- Extension of the steering committee members;
- Extension of the data collection related to dialysis treatment and blood count;
- Deleting any referring to initial patient name;
- Updating reference law concerning the protection of personal data.

Amendment n° 2

Amendment type: substantial

Amendment date: October 19th 2010

Amendment content

- Revision of the primary end point with sample size reformulation due to slow recruitment in the context of the publication of the TREAT study ("Trial to Reduce Cardiovascular Events with Aranesp Therapy", showing an increased risk of ictus in patients receving darbepoetin to achieve Hb elevels of around 13 g/dL compared to placebo) and a more conservative approach to ESA treatment adopted in the clinical practice.

  Primary end point in the original protocol was:

  Composite endpoint of death from any cause and cardiovascular events including nonfatal myocardial infarction, nonfatal stroke, or hospitalization for cardiovascular cause (acute coronary syndrome, transient ischemic attack, unplanned coronary revascularization, or peripheral arterial revascularization).

  The original sample size was:

  2104 adults to detect a risk reduction in the primary composite end-point with the experimental intervention (high fixed ESA dose) of 15% (HR=0.85) at 4 years with an expected annual incidence of

the primary composite endpoint of 15% based on data from existing trials. This number of participants would have provided the study with a power of 80%, with a two–sided type 1 error of 5% and allowing for a non-adherence rate of 5%.

Reviewed primary end point and sample size are reported in the current protocol version 3.

- 18 months extension of the enrolment phase (30 months in total);
- Follow up period reduction (from 4 years to 12 months) in agreement with the primary end point revision.

Amendment n° 3

Amendment type: substantial

Amendment date: May 27th 2014

Amendment content:

- Early completion of the recruitment phase (656 patients in total) due to slow recruitment and convergence of ESA dose and primary end-points in the two treatment groups.
