# Supplementary material for: Low versus high dose erythropoiesis-stimulating agents in hemodialysis patients with anemia: A randomized clinical trial
Source: PLoS One. 2017 Mar 1;12(3):e0172735. doi: 10.1371/journal.pone.0172735 (PMC5332066; doi:10.1371/journal.pone.0172735)
Supplement: S2 File — (PDF) [file pone.0172735.s008.pdf]

Effetti della dose degli agenti stimolanti l'eritropoiesi su esiti cardio-cerebrovascolari, qualità di vita in emodialisi.
The Clinical Evaluation of the DOSe of Erythropoietins (C.E. DOSE) trial

Studio approvato e finanziato dall'Agenzia Italiana del Farmaco (AIFA) nell'ambito del programma per la ricerca indipendente sui farmaci anno 2006

Protocollo dello studio
N. FARM6X822T

Versione finale n° 3.0
27 maggio 2014

Firma del Presidente del Comitato Scientifico

Protocollo Studio C.E. DOSE

Approvato da:

Giovanni FM Strippoli, M.D.
Fondazione Mario Negri Sud
Via Nazionale 8/a
66030 Santa Maria Imbaro (CH)

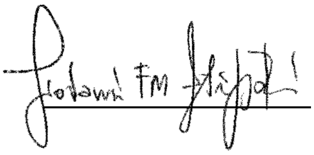

27/05/2014

firma data

Firma del Responsabile Medico del Comitato Scientifico

Protocollo Studio C.E. DOSE

Approvato da:

Gianni Tognoni, M.D.
Direttore Scientifico Fondazione Mario Negri Sud
Via Nazionale 8/a
66030 Santa Maria Imbaro (CH)

Mi impegno a seguire questo studio in accordo con quanto stipulato nel protocollo, con le Buone Norme di Pratica Clinica (GCP) e con la normativa vigente.

	27/05/2014
firma	data

Firma del Responsabile del Quality Team

Protocollo Studio C.E. DOSE

Miriam Valentini, M.D.
Fondazione Mario Negri Sud
Via Nazionale 8/a
66030 Santa Maria Imbaro (CH)

Mi impegno a seguire questo studio in accordo con quanto stipulato nel protocollo, con le Buone Norma di Pratica Clinica (GCP) e con la normativa vigente.

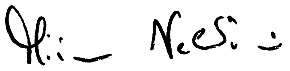

27/05/2014

firma data

Centro di Coordinamento

È possibile contattare il Centro di Coordinamento dal lunedì al venerdì dalle 9:00 alle 18:00.

Dott.ssa Valeria Saglimbene
Dipartimento di Farmacologia Clinica e Epidemiologia
Fondazione Mario Negri Sud
Via Nazionale 8/A
66030 S. Maria Imbaro (CH)
Tel. 0872 570.257.260.261
Fax 0872 570263
e-mail: cedose@negrisud.it

Firma del Medico Ricercatore

Protocollo Studio C.E. DOSE

Prof./Dott. ______________________________

Reparto ______________________________

Ospedale ______________________________

Indirizzo ______________________________

Città ______________________________

Mi impegno a condurre questo studio in accordo con quanto stipulato nel protocollo, con le Buone Norma di Pratica Clinica (GCP) e con la normativa vigente.

__________________ ______________

firma data

(Medico Ricercatore)

INDICE

INDICE ... 7

ORGANIZZAZIONE DELLO STUDIO ... 9

Steering Committee ... 9
Centro di Coordinamento ... 9
ABBREVIAZIONI RICORRENTI ... 10

SINTESI DEGLI ASPETTI PRATICI DELLO STUDIO ... 11

1. RAZIONALE ... 12

2. OBIETTIVI DELLO STUDIO ... 13

3. POPOLAZIONE IN STUDIO ... 13

4. DISEGNO DELLO STUDIO ... 13

5. TRATTAMENTI ... 15

6. VALUTAZIONE COMPARATIVA DEI TRATTAMENTI ... 15

6.1. Valutazione della qualità di vita ... 17

7. MODALITÀ OPERATIVE ... 17

7.1. Processo di informazione e coinvolgimento ... 17
7.2. Randomizzazione ... 17
7.3. Gestione del farmaco in studio ... 17
7.4. Visita basale (randomizzazione-tempo 0) ... 18
7.5. Visite di follow-up (tempo mesi 1, 2, 3, 6, 12) ... 18
7.6. Cointerventi terapeutici ... 18
7.7. Interruzione del trattamento ... 19
7.8. Uscita dallo studio ... 19

8. GESTIONE EVENTI AVVERSI ... 19

9. DIMENSIONE CAMPIONARIA DELLO STUDIO ... 19

10. DURATA DELLO STUDIO ... 20

11. ASPETTI STATISTICI E ANALISI DEI DATI ... 20

11.1. Analisi dei dati ... 20
11.2 Analisi ad interim ... 21

12. GESTIONE DEI DATI ... 21

12.1. Raccolta dei dati ... 21
12.2. Gestione dei dati su database e controllo di qualità ... 22

13. PUBBLICAZIONI ... 22

14. EMENDAMENTI AL PROTOCOLLO, ALTRI CAMBIAMENTI NELLA CONDUZIONE DELLO STUDIO ... 22

14.1. Emendamenti al protocollo ... 22
14.2. Altri cambiamenti nella conduzione dello studio ... 22

15. ALTRE INFORMAZIONI 22
BIBLIOGRAFIA 23
APPENDICE 1 24
ALGORITMO TERAPEUTICO 24
APPENDICE 2 30
DEFINIZIONE DEGLI ESITI CLINICI 30
APPENDICE 3 32
TIMETABLE DELLO STUDIO 32
APPENDICE 4 33
STANDARDS DI CURA 33
APPENDICE 5 34
EVENTI AVVERSI ed END POINT 34
Eventi avversi 34
Gestione e notifica eventi avversi 34
Eventi avversi gravi 34
Gestione e notifica eventi avversi 35
Gestione e notifica degli end-point 35
APPENDICE 6 36
STRUTTURA ORGANIZZATIVA E AREE DI RESPONSABILITÀ 36
Steering Committee 36
Data Safety and Monitoring Committee 36
Centro di Coordinamento 36
Quality Team 36
Centri partecipanti 36
APPENDICE 7 37
ATTIVITÀ DI MONITORAGGIO 37
Steering Committee 37
Data and Safety Monitoring Committee (DSMC) 37
End-point Committee 37
PROCEDURE DI MONITORAGGIO 38
REGISTRAZIONE DEI DATI E ARCHIVIAZIONE DEI DOCUMENTI 38
Protezione dei dati personali 39
Finalità e modalità di raccolta e trattamento dei dati 39
Ambito di comunicazione e di diffusione 39
Diritti dell'interessato ai sensi dell'Art. 7, D. Lgs. 196/03 39
APPENDICE 8 40
DIVULGAZIONE DELLE INFORMAZIONI, ETICA E CONFIDENZIALITÀ 40
SOSPENSIONE DELLO STUDIO 40
ETICA E GOOD CLINICAL PRACTICE 40
Comitato Etico Indipendente 41
Consenso informato 41
APPENDICE 9 42
DICHIARAZIONE DI HELSINKI 42
A. Introduzione 42
B. Principi basilari per tutta la ricerca medica 43
C. Principi aggiuntivi per la ricerca medica associata alle cure mediche 44

ORGANIZZAZIONE DELLO STUDIO

Steering Committee

Dr. Craig Jonathan C, School of Public Health University of Sydney, Australia

Dr. Di Giulio Salvatore, A.O. San Camillo Forlanini, Roma, Italia

Dr. Nicolucci Antonio, Fondazione Mario Negri Sud, S. Maria Imbaro (CH), Italia

Dr. Pellegrini Fabio, Fondazione Mario Negri Sud, S. Maria Imbaro (CH), Italia

Dr. Perkovic Vlado, the George Institute for International Health, Sydney, Australia

Dr. Procaccini Deni A, Divisione di Nefrologia, Ospedali Riuniti, Foggia, Italia

Dr. Santoro Antonio, U.O di Nefrologia e Dialisi, Policlinico S. Orsola – Malpighi, Bologna, Italia

Dr. Strippoli Giovanni FM, (Study Coordinator and Chairman Steering Committee) Fondazione Mario Negri Sud, S. Maria Imbaro (CH), Italia; School of Public Health University of Sydney, Australia

Dr. Strippoli Paolo, Reparto di Nefrologia e Dialisi, Presidio Ospedaliero " A. Perrino", Brindisi, Italia

Dr. Tognoni Giovanni, (Principal Investigator) Fondazione Mario Negri Sud, S. Maria Imbaro (CH), Italia

Dr. Triolo Giorgio, U.O.A. Nefrologia e Dialisi Azienda Ospedaliera, C.T.O./C.R.F./ M. Adelaide, Torino, Italia

Centro di Coordinamento

Dipartimento di Farmacologia Clinica e Epidemiologia
Fondazione Mario Negri Sud
Via Nazionale 8/A
66030 S. Maria Imbaro (CH)
Tel. 0872 570.257.260.261
Fax 0872 570263
e-mail: cedose@negrisud.it

ABBREVIAZIONI RICORRENTI

Hb	Emoglobina
ESA	Erythropoiesis stimulating agents (agenti stimolanti l'eritropoiesi)
CKD	Chronic Kidney Disease
ESKD	End stage kidney disease (insufficienza renale terminale)
QDV	Qualità di vita
RCT	Randomized controlled trial (studio randomizzato controllato)
PROBE	Prospective Randomized Open Blinded End-Point
r-HuEPO	recombinant-Human Erythropoietin
CRF	Case Report Form (scheda raccolta dati)

SINTESI DEGLI ASPETTI PRATICI DELLO STUDIO

POTENZIALMENTE ELEGGIBILI

- Soggetti di sesso maschile/femminile (età ≥ 18 anni)
- Soggetti con ESKD ed anemia in trattamento sostitutivo con dialisi extracorporea (bicarbonato-dialisi, emodialisi, emodiafiltrazione, emofiltrazione, acetate-free biofiltration)
- Soggetti che non presentino controindicazioni al trattamento con ESA o che siano già in trattamento con questi farmaci

VISITA DI RANDOMIZZAZIONE (tempo 0)

- Presa visione e firma del consenso informato
- Randomizzazione e assegnazione della terapia
- Anamnesi del paziente
- Esami ematochimici: profilo lipidico, glicemico, valutazione della funzionalità epatica e renale, esame emocromocitometrico completo
- Rilevazione dei livelli di emoglobina
- Dettagli relativi alla dialisi (peso secco, incremento ponderale interdialitico, flusso del sangue in dialisi, nPCR, Kt/V, durata e tipo di dialisi, filtro)
- Misurazione della pressione arteriosa sistolica e diastolica e della frequenza cardiaca
- Dettagli delle terapie intradialitiche e/o domiciliari relative all'ESKD
- Dettagli relativi alle altre terapie in uso (nome commerciale, dosaggio, posologia, indicazione)
- Rilevazione delle patologie concomitanti
- Questionario sulla qualità della vita

VISITA DI FOLLOW-UP A 1, 2, 3 MESI

- Esami ematochimici: Htc, MCV, MCH, MCHC, piastrinemia
- Rilevazione dei livelli di emoglobina
- Misurazione della pressione arteriosa sistolica e diastolica e della frequenza cardiaca
- Dettagli relativi alla dialisi (peso secco, incremento ponderale interdialitico, flusso del sangue in dialisi, nPCR, Kt/V, durata e tipo di dialisi, filtro)
- Dettagli delle terapie intradialitiche e/o domiciliari relative all'ESKD
- Dettagli relativi alle altre terapie in uso (nome commerciale, dosaggio, posologia, indicazione)
- Valutazione del valore di Hb riportato a seguito della terapia assegnata. Nel caso in cui i livelli di Hb (in seguito a duplice rilevazione) siano inferiori a 9.5 g/dL o superino i 12.5 g/dL, il dosaggio della terapia con ESA nei due gruppi di trattamento dovrà essere modificato con progressivi aumenti o riduzioni del 25% secondo le indicazioni della normale pratica. Un algoritmo terapeutico per l'agevole gestione clinica di queste modifiche (valido per eritropoietina e darbepoietina e facilmente applicabile per altre eritropoietine) è presente nel protocollo.

VISITA DI FOLLOW-UP A 6, 12

- Esami ematochimici: profilo lipidico, glicemico, valutazione della funzionalità epatica e renale, esame emocromocitometrico completo
- Rilevazione dei livelli di emoglobina
- Misurazione della pressione arteriosa sistolica e diastolica e della frequenza cardiaca
- Dettagli relativi alla dialisi (peso secco, incremento ponderale interdialitico, flusso del sangue in dialisi, nPCR, Kt/V, durata e tipo di dialisi, filtro)
- Dettagli delle terapie intradialitiche e/o domiciliari relative all'ESKD
- Dettagli relativi alle altre terapie in uso (nome commerciale, dosaggio, posologia, indicazione)
- Valutazione del valore di Hb riportato a seguito della terapia assegnata. Nel caso in cui i livelli di Hb (in seguito a duplice rilevazione) siano inferiori a 9.5 g/dL o superino i 12.5 g/dL, il dosaggio della terapia con ESA nei due gruppi di trattamento dovrà essere modificato con progressivi aumenti o riduzioni del 25% secondo le indicazioni della normale pratica clinica. Un algoritmo terapeutico per l'agevole gestione clinica di queste modifiche (valido per eritropoietina e darbepoietina e facilmente applicabile per altre eritropoietine) è presente nel protocollo.
- Questionario sulla qualità della vita

MONITORAGGIO DELLA SICUREZZA E DELL'EFFICACIA

- Monitoraggio centrale della sicurezza e dell'efficacia da parte del Data Safety and Monitoring Committee (DSMC) in aperto e monitoraggio qualitativo degli outcome da parte dell'End-point Committee in cieco

1. RAZIONALE

L'anemia è una condizione che interessa una elevata percentuale di soggetti con insufficienza renale cronica ed in particolare circa il 5% dei soggetti con insufficienza renale nella fase 1-2, il 2-10% di soggetti in fase 3 (1) secondo la classificazione Kidney Disease Outcomes Quality Initiative (K-DOQI) e quasi la totalità dei soggetti con insufficienza renale terminale (end stage kidney disease, ESKD) (1). L'utilizzo di agenti stimolanti l'eritropoiesi (Erythropoietin Stimulating Agents, ESA) rappresenta la principale e consolidata strategia terapeutica per il controllo dell'anemia nei soggetti con insufficienza renale cronica (Chronic Kidney Disease, CKD).

Studi osservazionali (2,3) hanno messo in evidenza che, nei soggetti con ESKD in trattamento sostitutivo emodialitico ed in terapia con ESA, livelli di emoglobina (Hb) progressivamente più bassi (inferiori a 11.0 g/dL) sono associati ad una prognosi sfavorevole in termini di sopravvivenza (incremento del rischio di morte per tutte le cause) ed ospedalizzazioni. D'altra parte valori progressivamente più elevati di Hb si associano invece ad una migliore qualità di vita (QdV) e ad una riduzione del rischio di eventi cardio-cerebrovascolari, ospedalizzazione e morte. Questi dati derivanti da grosse casistiche di registro secondo modelli di studio osservazionale non hanno trovato conferma in studi clinici randomizzati. Detti studi (4-6), che hanno arruolato oltre 5000 pazienti con CKD di vario stadio, dimostrano infatti evidenza contraria. La terapia con ESA mirante al raggiungimento di livelli di Hb vicini ai valori considerati "normali" (Hb ≥ 13.0 g/dL) determina un incremento significativo del rischio di eventi cardio-cerebrovascolari fatali e non fatali, pari a circa il 30%. Due meta-analisi di studi randomizzati disponibili in letteratura confermano l'incremento del rischio di eventi cardio-cerebrovascolari in soggetti trattati con ESA in cui si raggiungano valori di Hb>13.0 (7,8); i singoli trials e le loro meta-analisi inoltre individuano un miglioramento della QdV con il ricorso a valori target di Hb progressivamente più elevati.

In considerazione di questi dati, attualmente si ritiene che la correzione dello stato di anemia, seppur importante per una buona QdV, non debba essere spinta oltre livelli di Hb >13.0 g/dL, pena un incremento del rischio cardiovascolare.

Sempre alla luce dei risultati di questi recenti studi (4-6), le principali agenzie del farmaco, inclusa l'Agenzia Italiana del Farmaco (A.I.F.A.) e la Food and Drug Administration (F.D.A.), raccomandano di somministrare il dosaggio minore possibile di ESA utile a mantenere la concentrazione di Hb nel range di 10.0-12.0 g/dL.

Il motivo per cui l'utilizzo di dosaggi progressivamente più elevati di ESA per raggiungere livelli normali di Hb determina un incremento del rischio cardio-cerebrovascolare non è chiaro. Esiste la possibilità di una tossicità dose-dipendente: è possibile che l'incremento di mortalità sia direttamente correlato ai livelli di Hb o che esista una tossicità degli ESA dose-dipendente, non correlata all'emoglobinemia in via diretta. Quest'ultima ipotesi sarebbe avvalorata dal noto, anche se non totalmente definito, fenomeno dell'ESA resistenza, per il quale una risposta pronta a dosi medie di ESA non si osserva in alcune categorie di soggetti in cui il perseguimento di un valore target fisso elevato di Hb induce il ricorso a dosi particolarmente elevate di ESA. È plausibile che questi soggetti sperimentino un danno cardiovascolare ESA dose-dipendente e non soltanto un incremento dei valori di Hb. Non sono tuttavia mai stati condotti studi clinici in cui tale ipotesi ed i relativi meccanismi siano stati formalmente testati, né sono chiari i criteri che definiscono il fenomeno della ESA resistenza o ipersensibilità.

Al fine di individuare un approccio ottimale per il controllo dell'anemia in pazienti in dialisi, restano attivi i seguenti quesiti:

- quale sia il motivo, da un punto di vista meccanicistico, per cui alcuni pazienti rispondano prontamente a dosi medie di ESA rispetto ad altri che necessitano di dosi più elevate che possono risultare tossiche;
- quale sia il dosaggio di ESA che consenta una riduzione del rischio di eventi cardiovascolari, di mortalità e di eventi avversi correlati all'anemia;
- quale sia il dosaggio di ESA che si associ ad un miglioramento nella percezione soggettiva del paziente rispetto al proprio stato di salute, espresso in termini di QdV;

2. OBIETTIVI DELLO STUDIO

Obiettivi dello studio C.E. DOSE sono i seguenti:

1. valutare il rapporto tra dose ed effetto con una focalizzazione specifica su markers biochimici al fine di meglio comprendere i determinanti/predittori di una variabilità individuale di risposta alla terapia con ESA, sia rispetto alla efficacia che alla sicurezza (specificamente, esplorando l'associazione tra indicatori routinariamente testati nella pratica clinica (stato del ferro corporeo e dell'infiammazione);
2. produrre con questo approccio informazioni mirate per guidare e monitorare in modo efficiente la terapia a partire da dosi minori;
3. valutare l'efficacia comparativa di due differenti strategie terapeutiche basate sull'utilizzo di un dosaggio minimale o massimale di ESA relativamente a esiti clinici (mortalità, eventi cardio-cerebrovascolari, sicurezza dei trattamenti e qualità di vita);

3. POPOLAZIONE IN STUDIO

La popolazione eleggibile è costituita da soggetti adulti (età≥ 18 anni) che rispondano ai seguenti criteri di inclusione:

- soggetti con ESKD ed anemia in trattamento sostitutivo con dialisi extracorporea (bicarbonato-dialisi, emodiafiltrazione, emofiltrazione, emodiafiltrazione on-line, acetate-free biofiltration);
- soggetti che non abbiano controindicazioni al trattamento con ESA o che siano già in trattamento con questi farmaci.

Poiché lo studio confronta una strategia di trattamento basata sul dosaggio minimale ed una basata sul dosaggio massimale di ESA con il ricorso ad una randomizzazione, indipendentemente dalla terapia in corso, non potranno essere inclusi soggetti che presentino valori di Hb >10 g/dL senza terapia con ESA.

4. DISEGNO DELLO STUDIO

Si tratta di uno studio clinico randomizzato, multicentrico, aperto, con valutazione in cieco degli esiti ad opera di una commissione esterna (PROBE-Prospective Randomized Open Blinded End-Point). I pazienti eleggibili verranno randomizzati (1:1) a ricevere due dosi fisse di ESA ed in particolare la dose di 4000 UI/settimana ev. di eritropoietina alfa o beta o dosi equivalenti di qualsiasi altra eritropoietina disponibile in commercio verso la dose di 18000 UI/settimana ev. di eritropoietina alfa o beta o dosi equivalenti di qualsiasi altra eritropoietina disponibile in commercio.

Lo studio prevede un follow-up di 1 anno nel corso del quale verrà valutato il rapporto tra dose di ESA (minimale verso massimale) ed effetto, con una focalizzazione specifica su markers biochimici, sia rispetto alla efficacia che alla sicurezza. Verranno anche misurati gli effetti dei due interventi (dosaggio minimale vs dosaggio massimale) su un composito di esiti cardio-cerebrovascolari maggiori fatali e non fatali ed ospedalizzazioni per cause cardio-cerebrovascolari; verrà anche valutato l'effetto delle due strategie terapeutiche sulla QdV e su indicatori complessivi di praticabilità/fattibilità delle due strategie terapeutiche. Il disegno dello studio è presentato in Figura 1.

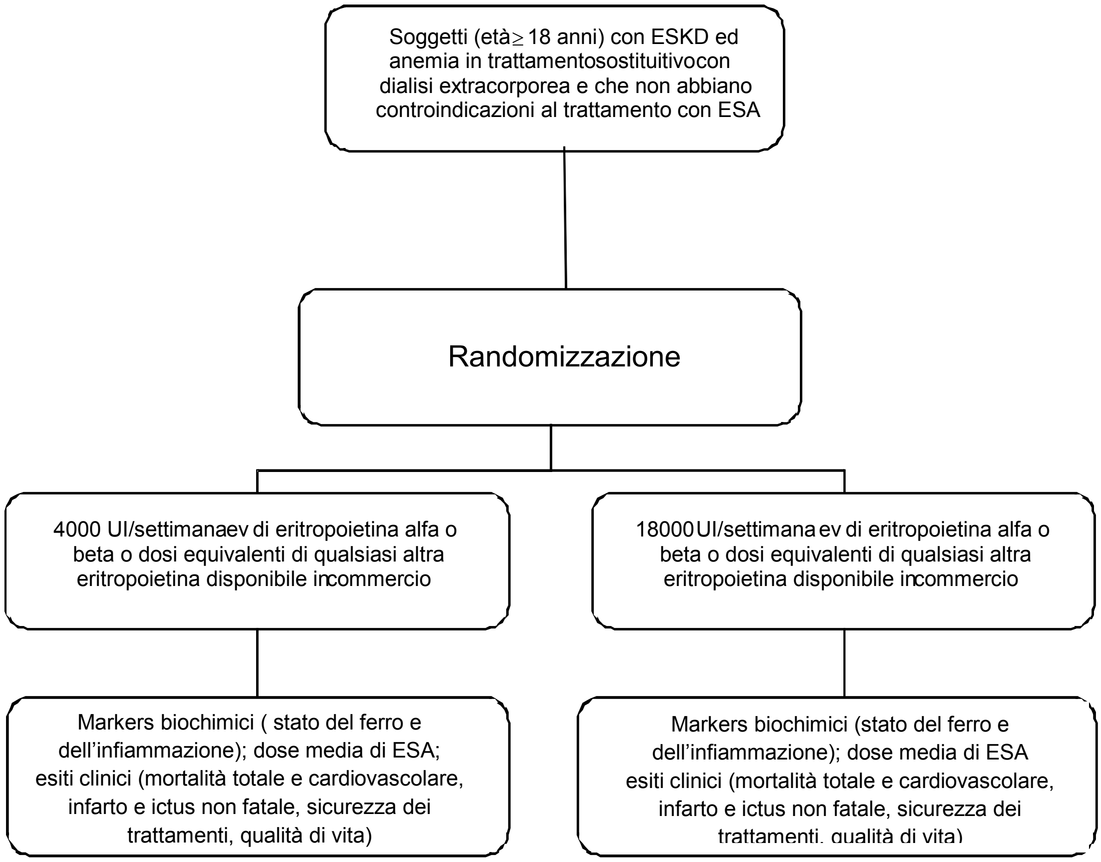

Figura 1 - Flow-chart indicante il processo di selezione, randomizzazione e follow-up dei pazienti coinvolti nello studio

5. TRATTAMENTI

I pazienti verranno randomizzati a ricevere 4000 UI/settimana ev. o 18000 UI/settimana ev. di eritropoietina alfa o beta o dosi equivalenti di qualsiasi altra eritropoietina disponibile in commercio. Laddove ne sussista la necessità (es. darbepoietina) si provvederà alla conversione del dosaggio del farmaco sperimentale nella dose equivalente del prodotto prescelto, seguendo gli algoritmi di conversione disponibili. Un esempio dell'algoritmo terapeutico di conversione eritropoieina-darbepoietina è fornito nell'appendice 1.

In considerazione del fatto che alla randomizzazione a questi interventi sperimentali faranno seguito oscillazioni dei valori di Hb che potrebbero essere al di fuori di un range di "sicurezza" (Hb 10.0-12.0 g/dL), come indicato dall'A.I.F.A., nel corso di questo studio, allorquando i valori di Hb scendessero al di sotto di 9.5 g/dL o si portassero al di sopra di 12.5 g/dL (valore confermato in due differenti rilevazioni), si procederà a modificare la dose nel senso di un aumento o diminuzione mirando al raggiungimento di valori di Hb tra 10.0 e 12.0 g/dL. Un algoritmo terapeutico per l'agevole gestione clinica di queste modifiche (valido per eritropoietina e darbepoietina) è allegato in appendice 1.

6. VALUTAZIONE COMPARATIVA DEI TRATTAMENTI

Lo studio C.E. DOSE si propone di valutare il rapporto tra dose della terapia con ESA somministrata e profilo biochimico della risposta/resistenza agli ESA stessi.

Sarà pertanto valutato l'effetto di due dosi fisse di ESA sul seguente esito primario, possibile marcatore di ESA responsività/resistenza:

→differenza a fine trattamento nei valori medi di:

ferritinemia, transferrinemia, TSAT (%), proteina C reattiva (PCR), albuminemia e dose media di ESA utilizzata tra il gruppo sperimentale e il gruppo di controllo

In aggiunta, sarà valutata la fattibilità dei due approcci terapeutici in termini di gestibilità clinica, misurando i seguenti esiti secondari:

- numero di soggetti in ciascun braccio in grado di mantenere valori di Hb tra 10.0 e 12.0 g/dL senza che siano necessarie modifiche superiori al 50% della dose iniziale assegnata all'epoca della randomizzazione;
- numero totale di variazioni del dosaggio di ESA effettuate dalla data della randomizzazione all'epoca in cui i valori di Hb si stabilizzano (2 rilevazioni consecutive) tra 10.0 e 12.0 g/dL;
- entità delle variazioni di dosaggio di ESA effettuate rispetto alla dose assegnata alla randomizzazione (espressa come valore medio in UI/settimana per le epoetine o microg/settimana per la darbepoetina) nei due bracci di trattamento;
- variazione media del dosaggio di ESA in base al peso corporeo ed all'indice di massa corporea;
- tempo dalla randomizzazione alla 1° variazione di dosaggio di ESA;
- tempo necessario dalla randomizzazione alla stabilizzazione dei livelli di Hb riferimento tra 10.0 e 12.0 g/dL;

- numero di pazienti che necessitano di trasfusioni di sangue.

Infine, sarà indagata l'efficacia dei trattamenti in studio, valutando anche l'impatto delle due diverse strategie terapeutiche relativamente ai seguenti esiti clinici:

- mortalità totale,
- infarto fatale
- infarto non fatale,
- ictus fatale
- ictus non fatale,
- ospedalizzazioni per sindromi coronariche acute, attacchi ischemici transitori, interventi di rivascolarizzazione coronarica non programmati ed interventi di rivascolarizzazione periferica
- qualità di vita valutata con il ricorso al questionario KDQOL-SF^{TM}1.3 (già disponibile e validato in lingua italiana)
- sicurezza dei trattamenti (trombosi dell'accesso vascolare, convulsioni, eventi ipertensivi).

Le definizioni relative agli esiti clinici sono riportati in appendice 2.

6.1. Valutazione della qualità di vita

La valutazione della QdV sarà eseguita con il ricorso al questionario KDQOL-SF™1.3.

Tale strumento comprende 18 scale: 8 relative allo strumento SF-36 (funzionalità fisica, limitazioni di ruolo dovute a problemi fisici, dolore fisico, salute in generale, vitalità, limitazioni di ruolo dovute a problemi emotivi, attività sociali, salute mentale) e 10 relative allo strumento KDQOL (sintomi/problemi, effetti della patologia renale, impatto della patologia renale, stato occupazionale, funzioni cognitive, qualità delle relazioni sociali, attività sessuale, qualità del sonno, supporto sociale, soddisfazione del paziente).

Per ciascuna scala è possibile un punteggio variabile da 0 a 100 e a punteggi più alti corrisponde una migliore QdV.

Nell'ambito dell'analisi, anche al fine di confrontare la gestibilità e fattibilità delle due strategie di trattamento, si procederà in primo luogo al calcolo del numero di pazienti nei due bracci di trattamento che presentano una riduzione della funzionalità fisica ritenuta clinicamente rilevante (Effect Size ≥ 0.5 sulla scala della Physical Functioning-Attività Fisica del questionario SF-36). In analisi secondarie, le valutazioni verranno estese a tutte le dimensioni della QdV indagate.

7. MODALITÀ OPERATIVE

Dopo aver verificato l'eleggibilità di un paziente, la sua partecipazione allo studio prevede le seguenti procedure:

7.1. Processo di informazione e coinvolgimento

L'inclusione di un paziente nello studio deve essere preceduta da un'approfondita discussione del razionale e delle finalità dello studio, facendo riferimento a quanto riportato nel modulo informativo. La volontà del soggetto di partecipare andrà ratificata tramite firma del modulo di consenso informato, secondo quanto richiesto dalle norme di buona pratica clinica (GCP-ICH).

7.2. Randomizzazione

L'assegnazione del trattamento in studio avverrà tramite randomizzazione telefonica centralizzata al numero dedicato (Tel. 0872 570244) nel corso della prima visita, dopo aver verificato che il paziente soddisfi i criteri di eleggibilità e abbia firmato il consenso informato. Le liste di randomizzazione saranno stratificate per singolo centro arruolante. Per garantire che in ogni servizio arruolante il numero di pazienti assegnato a ciascun braccio sia bilanciato, saranno utilizzate liste di randomizzazione a blocchi permutati.

Ad ogni paziente verrà assegnato un numero di identificazione unico (Numero di Randomizzazione). Tale numero, assegnato dal Centro di Coordinamento, è composto da due parti. La prima parte è rappresentata dal numero del centro mentre l'ultima parte è rappresentata dal numero del soggetto.

7.3. Gestione del farmaco in studio

Dopo l'assegnazione del trattamento mediante randomizzazione, lo sperimentatore provvederà alla prescrizione di uno qualsiasi degli ESA disponibili in commercio, alla dose a cui è stato randomizzato. Al

paziente verrà consegnata una lettera di accompagnamento con indicazioni chiare sulla partecipazione allo studio, sul farmaco e sul dosaggio prescritto. Giacché trattasi di terapia per somministrazione cronica, qualora il paziente dovesse essere ospedalizzato o ricevere il trattamento di dialisi presso struttura esterna rispetto al centro sperimentatore, saranno rilasciate unitamente alla scheda di dialisi indicazioni chiare relative alla partecipazione allo studio ed al dosaggio in corso.

7.4. Visita basale (randomizzazione-tempo 0)

Dopo l'arruolamento, si procederà alla raccolta delle seguenti informazioni, così come previsto nella scheda di inizio studio: dati socio-demografici, anamnesi del paziente e tutti i dati clinici e laboratoristici relativi alla patologia in esame ed al rischio cardiovascolare (esame emocromocitometrico completo, profilo lipidico e glicemico, funzionalità renale, funzionalità epatica, valori di pressione arteriosa, patologie concomitanti). Verranno inoltre raccolte informazioni relative alla terapia dialitica, alla terapia farmacologica intra-dialitica e domiciliare ed alle altre terapie in corso. Infine, si procederà alla somministrazione del questionario di valutazione della QdV (KDQOL-SF^{TM}1.3).

7.5. Visite di follow-up (tempo mesi 1, 2, 3, 6, 12)

Dopo la visita di randomizzazione, le visite di follow-up successive sono previste ogni mese per i primi 3 mesi e successivamente a 6 e 12 mesi; queste visite comprendono la rivalutazione sistematica dell'incidenza degli esiti clinici oggetto dello studio e la rivalutazione dei parametri clinici e laboratoristici relativi alla patologia in esame, alla terapia dialitica, alla terapia farmacologica intradialitica e domiciliare ed alle altre terapie in corso. Per ogni paziente eleggibile tutti i dati raccolti nel corso di tali visite andranno riportati sulle apposite "Schede raccolta dati". Una descrizione più dettagliata della cadenza delle visite e della relativa rilevazione dei parametri è riportata nell' Appendice 3 "Timetable dello studio".

Inoltre nel corso di ogni visita semestrale verrà somministrato ai pazienti il questionario per la valutazione della QdV (KDQOL-SF^{TM}1.3). Questa frequenza delle visite è quanto strettamente previsto per la conduzione dello studio e non si sostituisce alle visite che classicamente i pazienti sottoposti a trattamento emodialitico ricevono ad ogni seduta dialitica ed a scadenza mensile per la valutazione della terapia dialitica, della terapia farmacologica intra-dialitica e della terapia domiciliare. Pertanto potranno essere effettuate visite aggiuntive nel corso delle quali verranno raccolti ulteriori informazioni qualora il medico lo ritenesse necessario, che potranno essere riportate su specifiche "Schede raccolta dati".

Il follow-up dovrà essere garantito per tutta la durata dello studio anche dopo l'eventuale interruzione precoce del trattamento o il verificarsi di uno degli eventi in studio.

Allorquando non sia più possibile garantire un follow-up completo (ad esempio pazienti trasferiti e non più rientranti presso il centro di emodialisi), è prevista la compilazione di un'apposita scheda di interruzione precoce dello studio.

7.6. Cointerventi terapeutici

I pazienti verranno seguiti in base alla normale pratica clinica e pertanto riceveranno in maniera non randomizzata qualsiasi altro farmaco di uso comune nella terapia intra- ed extra-dialitica. Una indicazione dei principali standards di cura cui il medico potrà, a suo imprescindibile giudizio, fare riferimento e che si

basano sulle indicazioni delle principali evidenze scientifiche, linee guida ed organizzazioni scientifiche italiane ed internazionali, è riportata in appendice 4.

7.7. Interruzione del trattamento

Trattandosi di uno studio gestito secondo la normale pratica clinica, le interruzioni dei trattamenti avverranno solo per indicazioni cliniche ritenute rilevanti dal medico sperimentatore. Tra queste, ad esempio, la tossicità clinicamente inaccettabile (peggioramento dell'ipertensione non altrimenti controllabile con il ricorso a farmaci anti-ipertensivi, eventi tromboembolici, convulsioni) o la interruzione volontaria da parte del paziente.

Tutti gli eventi saranno registrati indipendentemente dal fatto che il paziente riceva ancora il trattamento assegnato.

7.8. Uscita dallo studio

Un paziente uscirà dallo studio prima della sua fine solo in caso di decesso, ritiro del consenso o trasferimento. Un paziente uscito dallo studio potrà rientrarvi, se lo vorrà; in questo caso bisognerà ottenere un secondo consenso informato. Il reingresso nello studio non è condizionato da limiti di tempo e può verificarsi dopo giorni, settimane, mesi o anni.

I pazienti considerati persi al follow-up, ai fini dell'analisi rimarranno nello studio fino alla fine dello stesso o fino al loro decesso (se noto). Ad ogni visita fino alla fine dello studio o fino al decesso del paziente, qualsiasi sforzo andrà fatto per contattare il paziente e recuperare le informazioni. Almeno lo stato vitale dovrà essere riportato ad ogni visita programmata fino alla fine dello studio.

8. GESTIONE EVENTI AVVERSI

Poiché lo studio prevede farmaci già abitualmente adottati negli stessi pazienti nella pratica corrente vigono le normali regole di segnalazione delle reazioni avverse previste dal sistema di farmacovigilanza.

Una descrizione dettagliata del sistema di comunicazione degli eventi avversi è riportata in appendice 5.

9. DIMENSIONE CAMPIONARIA DELLO STUDIO

Al fine di individuare uno "small effect size" (0.2), con un α=0.05 ed un 1-β=0.80, per l'esito primario della differenza nei valori a fine trattamento di ferritinemia o transferrinemia o TSAT (%) o PCR o albuminemia e dose media di ESA utilizzata, nell'intera coorte di pazienti tra il gruppo sperimentale (bassi dosaggi di ESA) ed il gruppo di controllo, sarà necessaria una dimensione campionaria di 900 soggetti (n=450 per gruppo).

Al fine poi di individuare un "medium effect size' (0.5)", con un α=0.05 ed un 1-β=0.80, per lo stesso esito nel solo sottogruppo di soggetti ESA resistenti/iperesensibili, ipotizzando che esso sia pari al 15% del campione totale, sarà necessaria una dimensione campionaria pari a 150 soggetti (n=75 per gruppo).

La dimensione campionaria prevista di 900 soggetti permetterà inoltre di rilevare una riduzione attesa del 30% nell'esito composito di mortalità totale, infarto fatale e non fatale, ictus fatale e non fatale, ospedalizzazioni per cause cardiovascolari nel gruppo sperimentale rispetto al gruppo di controllo qualora essa esista, in presenza di:

- incidenza annuale di questo esito composito pari a 35%;
- potenza statistica 80% (alfa=0.05);
- riduzione attesa del rischio dell'esito composito pari al 30% (HR=0.70%);
- drop-out pari al 5%.

10. DURATA DELLO STUDIO

La durata dell'arruolamento nello studio sarà di 30 mesi. La durata del periodo di follw-up dello studio sarà di mesi 12.

11. ASPETTI STATISTICI E ANALISI DEI DATI

11.1. Analisi dei dati

Tutte le valutazioni di efficacia saranno eseguite in base al principio dell'"intention to treat" (valutazione di tutti i pazienti inizialmente randomizzati, a prescindere dal completamento o meno della terapia assegnata). Le analisi comparative saranno anche eseguite nel solo sottogruppo dei soggetti ESA resistenti/ipersensibili.

La risposta dei pazienti all'eritropoietina (ESA resistenza/ipersensibilità) sarà valutata in base al:

- cambiamento % di Hb a due settimane aggiustato per la dose di ESA assunta al baseline
- rapporto tra il valore di Hb raggiunto e la dose di ESA somministrata alla fine dello studio, aggiustato per dose di ESA assunta al baseline
- rapporto tra il valore medio di Hb e la dose media di ESA aggiustato per la dose di ESA assunta al baseline
- numero totale di variazioni del dosaggio di ESA effettuate dalla data della randomizzazione all'epoca in cui i valori di Hb si stabilizzano (2 rilevazioni consecutive) tra 10.0 e 12.0 g/dL

La distribuzione della misura della risposta all'eritropoietina sarà categorizzata in quartili.

Le differenze medie per l'esito primario tra i due gruppi saranno valutate con i modelli lineari gerarchici con una struttura di covarianza di tipo simmetrico, tenendo conto dei valori di baseline. La misura finale dell'esito sarà quindi espressa come differenza pre-post delle differenze tra gruppi.

L'incidenza degli eventi sarà stimata utilizzando le curve di Kaplan Meier, che saranno confrontate utilizzando il log-rank test. Saranno inoltre applicate analisi multivariate, utilizzando il modello di Cox.

Ulteriori analisi riguarderanno la valutazione di efficacia degli interventi in studio (alte dosi o basse dosi di ESA) su ognuno degli esiti dello studio e sulla QdV. Tali analisi verranno eseguite utilizzando la statistica univariata e multivariata (test parametrici e non parametrici in base alla tipologia dei dati).

L'efficacia clinica del trattamento e il suo impatto sulla QdV saranno inoltre valutati in relazione ad alcune caratteristiche dei pazienti alla data della randomizzazione:

- Caratteristiche socio-demografiche (età, sesso, etnia, stato civile, scolarità, stato occupazionale)
- Presenza/assenza di fattori di rischio cardiovascolare (diabete, ipertensione, fumo, familiarità per patologie cardio-cerebrovascolari)
- Presenza/assenza pregresso evento cardio-cerebrovascolare
- Presenza/assenza di altre patologie concomitanti ritenute rilevanti
- Tipo di ESA
- Dose media di ESA raggiunta durante lo studio
- Livello di controllo metabolico (quartili dei livelli di colesterolo e di HbA1c)
- Trattamento con altri farmaci (terapia anti-ipertensiva, terapia con ferro, terapia anticoagulante, trattamento con vitamina D o analoghi, con chelanti del fosforo, con calcio mimetici, con statine o con altri cointerventi terapeutici)
- Livelli di controllo pressorio
- Livelli di calcio, fosforo, ferro, paratormone; valori di Kt/V, peso secco, flusso dialisi, incremento ponderale interdialitico
- Tipo di dialisi
- Durata della dialisi

L'esistenza di eventuali trend nell'entità dei benefici in base ai sottogruppi identificati sarà testata utilizzando il test di Mantel-Haenszel, mentre il test del chi quadrato verrà utilizzato per testare l'eventuale eterogeneità nell'entità dei benefici nei diversi sottogruppi.

11.2 Analisi ad interim

Qualora lo Steering Committee lo ritenesse necessario, nel corso dello studio saranno condotte delle analisi ad interim di efficacia e sicurezza al fine di verificare le assunzioni fatte per la realizzazione dello studio e la necessità di proseguire con il reclutamento e/o il follow-up.

Indipendentemente dal numero di pazienti reclutati, si deciderà di interrompere il reclutamento se si verificherà almeno una delle seguenti condizioni:

- convergenza dei valori di emoglobina e delle dosi di ESA nei due bracci di trattamento,
- convergenza degli end point primari nei due bracci di trattamento.

12. GESTIONE DEI DATI

12.1. Raccolta dei dati

Tutte le informazioni richieste dovranno essere riportate all'interno delle schede raccolta dati; le schede saranno realizzate in carta autocopiante in modo da disporre di una doppia copia di ogni scheda. I monitor dovranno controllare la completezza e l'accuratezza dei dati raccolti ed istruire il personale dei centri partecipanti allo studio ad effettuare ogni correzione o aggiunta richiesta. Le schede raccolta dati in originale

con firma dello sperimentatore saranno inviate al Quality Team direttamente dai monitor o dal centro partecipante allo studio e una copia resterà presso il centro stesso. Una volta ricevute dal Quality Team, le schede saranno registrate e la copia originale sarà conservata in un archivio centrale. Le schede inviate al Quality Team saranno controllate prima dell'inserimento dei dati nel computer.

12.2. Gestione dei dati su database e controllo di qualità

Tutte le informazioni relative a questo studio verranno inserite in un apposito database gestito dal Quality Team, che curerà il controllo delle informazioni prima del loro inserimento nel database stesso. Il Centro di Coordinamento produrrà dei rapporti sugli errori rilevati tramite i programmi di controllo di qualità. Errori od omissioni saranno registrati su una apposita scheda di richiesta dati ("Data Clarification Form") che verrà inviata ai centri per la risoluzione del problema. Una copia firmata del "Data Clarification Form" dovrà essere conservata con la scheda raccolta dati presso il centro e, una volta che l'originale corretto sia pervenuto al Quality Team, le rettifiche saranno inserite nel database.

13. PUBBLICAZIONI

I risultati dello studio saranno presentati a congressi e pubblicati a nome dei membri dello Steering Committee che parteciperanno alla loro stesura, come e per conto del Gruppo Collaborativo C.E.DOSE. I nomi di tutti gli Sperimentatori e dei componenti dei vari Comitati saranno riportati in un'apposita appendice.

La responsabilità di pubblicazione è del Promotore dello studio.

Qualsiasi proposta per analisi ancillari da condurre nei pazienti randomizzati in studio dovrà essere approvata dallo Steering Committee prima che gli studi stessi abbiano inizio. Nel prendere in considerazione queste proposte, lo Steering Committee dovrà valutare che lo studio ancillare proposto sia di elevata qualità scientifica e che non comprometta in alcun modo i risultati, la conduzione e la presentazione dello studio principale (ad esempio, riducendo il tasso di reclutamento).

14. EMENDAMENTI AL PROTOCOLLO, ALTRI CAMBIAMENTI NELLA CONDUZIONE DELLO STUDIO

14.1. Emendamenti al protocollo

Qualsiasi cambiamento al protocollo sarà effettuato sotto forma di emendamento, approvato dallo Steering Committee.

14.2. Altri cambiamenti nella conduzione dello studio

Non sono permessi cambiamenti del piano di studio senza che sia stato emesso prima un emendamento al protocollo. Qualsiasi deviazione dal protocollo deve essere documentata sulla cartella clinica.

15. ALTRE INFORMAZIONI

Informazioni relative alla struttura organizzativa e aree di responsabilità, alle attività di monitoraggio, alla divulgazione di informazioni, etica e confidenzialità ed ai principi della ricerca clinica sono riportate nelle appendici 6, 7, 8 e 9.

BIBLIOGRAFIA

1. Hsu CY, McCulloch CE, Curhan GC. Epidemiology of anemia associated with chronic renal insufficiency among adults in the United States: results from the Third National Health and Nutrition Examination Survey. J Am Soc Nephrol. 2002;13:504-10.
2. Collins AJ, Li S, St Peter W, Ebben J, Roberts T, Ma JZ, Manning W. Death, hospitalization, and economic associations among incident hemodialysis patients with hematocrit values of 36 to 39%. J Am Soc Nephrol. 2001;12:2465-73.
3. Collins AJ, Ma JZ, Xia A, Ebben J. Trends in anemia treatment with erythropoietin usage and patient outcomes. Am J Kidney Dis. 1998;32(6 Suppl 4):S133-41.
4. Besarab A, Bolton WK, Browne JK, Egrie JC, Nissenson AR, Okamoto DM, Schwab SJ, Goodkin DA. The effects of normal as compared with low hematocrit values in patients with cardiac disease who are receiving hemodialysis and epoetin. N Engl J Med. 1998;339:584-90.
5. Singh AK, Szczech L, Tang KL, Barnhart H, Sapp S, Wolfson M, Reddan D; CHOIR Investigators. Correction of anemia with epoetin alfa in chronic kidney disease. N Engl J Med. 2006;355:2085-98.
6. Drüeke TB, Locatelli F, Clyne N, Eckardt KU, Macdougall IC, Tsakiris D, Burger HU, Scherhag A; CREATE Investigators. Normalization of hemoglobin level in patients with chronic kidney disease and anemia. N Engl J Med. 2006;355:2071-84.
7. Strippoli GF, Craig JC, Manno C, Schena FP. Hemoglobin targets for the anemia of chronic kidney disease: a meta-analysis of randomized, controlled trials. J Am Soc Nephrol. 2004;15:3154-65.
8. Strippoli GF, Tognoni G, Navaneethan SD, Nicolucci A, Craig JC. Haemoglobin targets: we were wrong, time to move on. Lancet 2007; 369:346-50.
9. Phrommintikul A, Haas SJ, Elsik M, Krum H. Mortality and target haemoglobin concentrations in anaemic patients with chronic kidney disease treated with erythropoietin: a meta-analysis. Lancet 2007 Feb 3;369:381-8.
10. Ware JE, Sherbourne CD. The MOS 36-item Short-Form Health Survey (SF-36). Medical Care 1992; 30:473-483.
11. Apolone G, Mosconi P. The Italian SF-36 Health Survey: translation, validation and norming. J Clin Epidemiol 1998; 51:1025-1036.
12. Labbrozzi D, Erle G, Meschi F, Nicolucci A, Apolone G, et al. La valutazione della qualità della vita nel paziente diabetico. Giornale Italiano di Diabetologia 1996; 16:69-78.

APPENDICE 1

ALGORITMO TERAPEUTICO

Poiché la somministrazione di questi farmaci si basa sulla disponibilità in commercio di siringhe pre-riempite in vario dosaggio e sulla possibilità di modificare la frequenza di somministrazione nell'arco della settimana (es. 1 somministrazione/settimana o 2-3 somministrazioni/settimana) è possibile il verificarsi di una variabilità inter/intra-centro. Al fine di evitare questa variabilità, è consigliabile ripartire la dose totale settimanale in 2-3 differenti somministrazioni di eritropoietina/settimana o in un'unica somministrazione di darbepoietina/settimana come di seguito e come, peraltro, è uso ricorrente nella normale pratica clinica:

- Gruppo n°1. il paziente viene randomizzato ad ESA 18000 UI/settimana e sarà trattato con:
  - EPO alfa 6000 UI x3/settimana o
  - EPO beta 6000 UI x3/settimana
  - darbepoietina 90 (40+50 o 60+30 o 80+10) microgrammi/1 volta settimana
  - dosi equivalenti di altro ESA

- Gruppo n°2. il paziente viene randomizzato ad ESA 4000 UI/settimana e sarà così trattato con:
  - EPO alfa 2000 UI x2/settimana o
  - EPO beta 2000 UI x2/settimana
  - o darbepoietina 20 microgrammi/1 volta settimana
  - o dosi equivalenti di altro ESA

Nel caso in cui i livelli di Hb (in seguito a duplice conferma) siano inferiori a 9.5 g/dL o superino i 12.5 g/dL, il dosaggio della terapia con ESA nei due gruppi di trattamento dovrà essere modificato con progressivi aumenti o riduzioni del 25% secondo le indicazioni della normale pratica clinica. Un algoritmo terapeutico per l'agevole gestione clinica di queste modifiche (valido per eritropoietina e darbepoietina) è riportato di seguito.

Tutte le rilevazioni di valori di Hb per le quali siano indicate modifiche del dosaggio di ESA devono essere riportate nella scheda “Modifiche del dosaggio di ESA”.

MODALITÀ DI RIDUZIONE DEL DOSAGGIO IN SOGGETTI RANDOMIZZATI AL DOSAGGIO FISSO ELEVATO (ESA 18000 UI/settimana o DARBO 90 microg/settimana ev.) NEL CASO DI PERSISTENZA DI VALORI DI Hb > 12.5 g/dL (scenario A)

Se nel gruppo 1 il valore di Hb misurato ad uno dei controlli mensili (e a seguito di un'ulteriore conferma) è >12.5 g/dL, il dosaggio dovrà essere ridotto, specificamente come segue:

- EPO alfa o beta 14000 UI/settimana o
- Darbepoietina 70 microg/settimana o
- dose equivalente di altro ESA

Se al controllo successivo (e a seguito di una ulteriore conferma) il valore di Hb persiste >12.5 g/dL, il dosaggio dovrà essere ridotto, specificamente come segue:

- EPO alfa o beta 10000 UI/settimana o
- Darbepoietina 50 microg/settimana o
- dose equivalente di altro ESA

Se al controllo successivo (e a seguito di una ulteriore conferma) il valore di Hb persiste >12.5 g/dL, il dosaggio dovrà essere ridotto, specificamente come segue:

- EPO alfa o beta 8000 UI/settimana o
- Darboepoietina 40 microg/settimana o
- dose equivalente di altro ESA

Se al controllo successivo (e a seguito di una ulteriore conferma) il valore di Hb persiste >12.5 g/dL, il dosaggio dovrà essere ridotto, specificamente come segue:

- EPO alfa o beta 6000 UI/settimana o
- Darboepoietina 30 microg/settimana o
- dose equivalente di altro ESA

Se al controllo successivo (e a seguito di una ulteriore conferma) il valore di Hb persiste >12.5 g/dL, il dosaggio dovrà essere ridotto, specificamente come segue:

- EPO alfa o beta 4000 UI/settimana o
- Darboepoietina 20 microg/settimana o
- dose equivalente di altro ESA

Successivamente, indipendentemente dalle visite previste per la conduzione dello studio (MESI 1, 2, 3, 6, 12), se nell'ambito di qualsivoglia controllo previsto per la normale gestione clinica dei trattamenti il valore di Hb persista >12.5 g/dL, a seguito di una ulteriore conferma, la somministrazione di ESA andrà interrotta fino a che i valori di Hb non scendano al di sotto di 12.5 g/dL.

MODALITÀ DI INCREMENTO DEL DOSAGGIO IN SOGGETTI RANDOMIZZATI AL DOSAGGIO FISSO ELEVATO (ESA 18000 UI/settimana o DARBO 90 microg/settimana ev.) NEL CASO DI PERSISTENZA DI VALORI DI Hb <9.5 g/dL (scenario B)

Se nel gruppo 1 il valore di Hb ad uno dei controlli (e a seguito di una ulteriore conferma) è <9.5 g/dL, il dosaggio dovrà essere aumentato, specificamente come segue:

- EPO alfa o beta 22000 UI/settimana o
- Darbepoietina 110 microg/settimana o
- dose equivalente di altro ESA

Se al controllo successivo (e a seguito di una ulteriore conferma) il valore di Hb persiste <9.5 g/dL, il dosaggio dovrà essere aumentato, specificamente come segue:

- EPO alfa o beta 28000 UI/settimana o
- Darbepoietina 140 microg/settimana o
- dose equivalente di altro ESA

Se al controllo successivo (e a seguito di una ulteriore conferma) il valore di Hb persiste <9.5 g/dL, il dosaggio dovrà essere aumentato, specificamente come segue:

- EPO alfa o beta 35000 UI/settimana o
- Darboepoietina 175 microg/settimana o
- dose equivalente di altro ESA

Successivamente, indipendentemente dalle visite previste per la conduzione dello studio (MESI 1, 2, 3, 6, 12), se nell’ambito di qualsivoglia controllo previsto per la normale gestione clinica dei trattamenti il valore di Hb persiste <9.5 g/dL a seguito di una ulteriore conferma il dosaggio dovrà permanere a dosaggi di 35000 EPO alfa o beta UI/settimana o Darboepoietina 175 microg/settimana.

MODALITÀ DI RIDUZIONE DEL DOSAGGIO IN SOGGETTI RANDOMIZZATI AL DOSAGGIO FISSO BASSO (ESA 4000 UI/settimana o DARBO 20 microg/settimana ev.) NEL CASO DI PERSISTENZA DI VALORI DI Hb >12.5 g/dL (scenario C)

Se nel gruppo 2 il valore di Hb ad uno dei controlli (e a seguito di una ulteriore conferma) è >12.5 g/dL, il dosaggio dovrà essere ridotto, specificamente come segue:

- EPO alfa o beta 3000 UI /settimana o
- Darbepoietina 15 microg/settimana o
- dose equivalente di altro ESA

Se nel gruppo 2 il valore di Hb al controllo (e a seguito di una ulteriore conferma) è >12.5 g/dL, il dosaggio dovrà essere ridotto, specificamente come segue:

- EPO alfa o beta 2000 UI /settimana
- Darbepoietina 10 microg/settimana
- dose equivalente di altro ESA

Successivamente, indipendentemente dalle visite previste per la conduzione dello studio (MESI 1, 2, 3, 6, 12), se nell'ambito di qualsivoglia controllo previsto per la normale gestione clinica dei trattamenti il valore di Hb persiste >12.5 g/dL, a seguito di una ulteriore conferma, la somministrazione di ESA andrà interrotta fino a che i valori di Hb non scendano al di sotto di 12.5 g/dL.

MODALITÀ DI INCREMENTO DEL DOSAGGIO IN SOGGETTI RANDOMIZZATI AL DOSAGGIO FISSO BASSO (ESA 4000 UI/settimana o DARBO 20 microg/settimana ev.) NEL CASO DI PERSISTENZA DI VALORI DI Hb <9.5 g/dL (scenario D)

Se nel gruppo 2 il valore di Hb ad uno dei controlli (e a seguito di una ulteriore conferma) è <9.5 g/dL, il dosaggio dovrà essere aumentato, specificamente come segue:

- EPO alfa o beta 5000 UI o
- Darbepoietina 25 microg/settimana o
- dose equivalente di altro ESA

Se al controllo successivo (e a seguito di una ulteriore conferma) il valore di Hb persiste <9.5 g/dL, il dosaggio dovrà essere aumentato, specificamente come segue:

- EPO alfa o beta 6000 UI/settimana o
- Darbepoietina 30 microg/settimana o
- dose equivalente di altro ESA

Se al controllo successivo (e a seguito di una ulteriore conferma) il valore di Hb persiste <9.5 g/dL, il dosaggio dovrà essere aumentato, specificamente come segue:

- EPO alfa o beta 8000 UI/settimana o
- Darboepoietina 40 microg/settimana o
- dose equivalente di altro ESA

Successivamente, indipendentemente dalle visite previste per la conduzione dello studio (MESI 1, 2, 3, 6, 12), se nell'ambito di qualsivoglia controllo mensile previsto per la normale gestione clinica dei trattamenti il valore di Hb persiste <9.5 g/dL a seguito di una ulteriore conferma il dosaggio potrà essere aumentato fino al raggiungimento di livelli di Hb >9.5 g/dL.

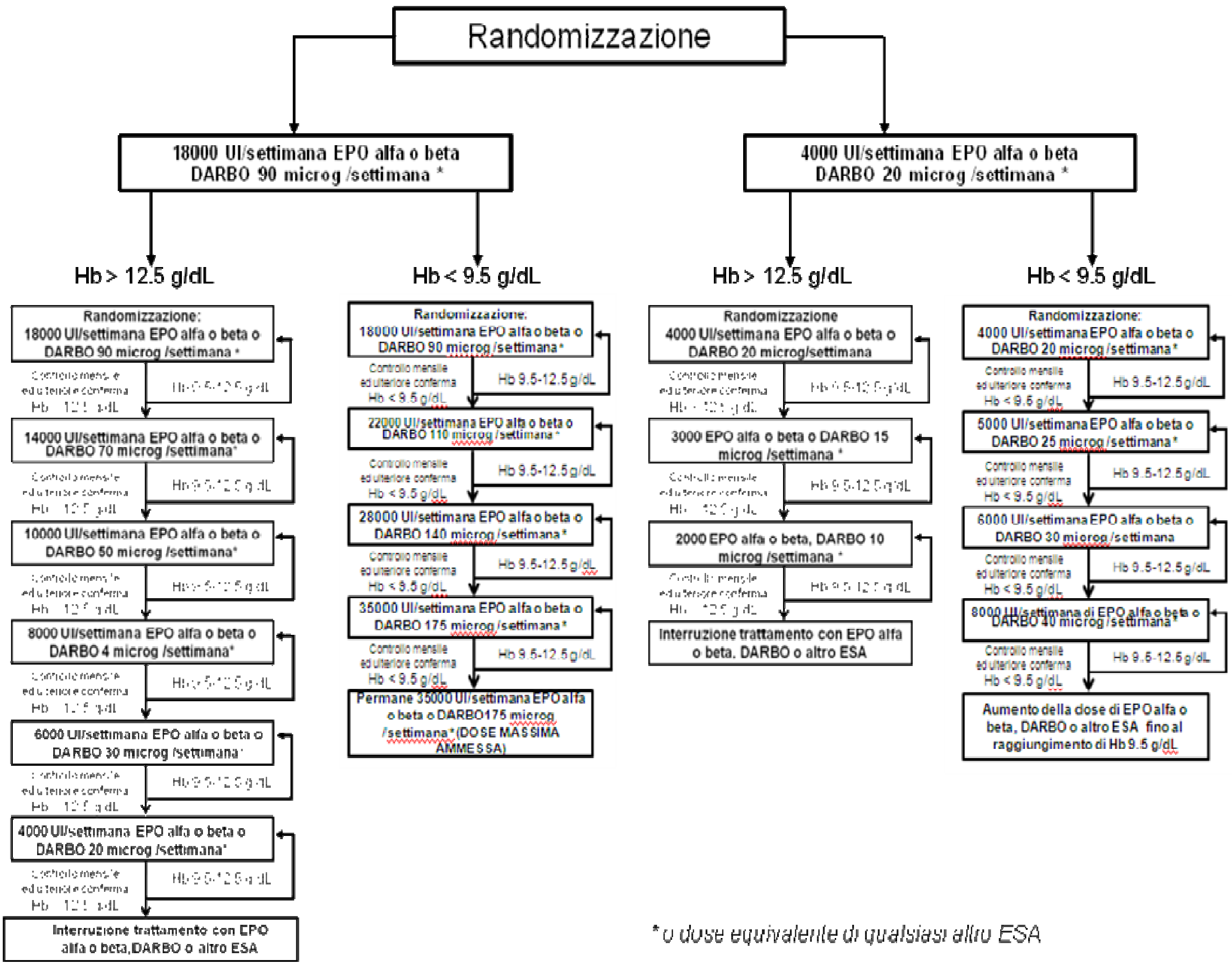
Randomizzazione
18000 UI/settimana EPO alfa o beta DARBO 90 microg /settimana *
4000 UI/settimana EPO alfa o beta DARBO 20 microg /settimana *
Hb > 12.5 g/dL
Hb < 9.5 g/dL
Hb > 12.5 g/dL
Hb < 9.5 g/dL
Randomizzazione: 18000 UI/settimana EPO alfa o beta o DARBO 90 microg /settimana *
14000 UI/settimana EPO alfa o beta o DARBO 70 microg /settimana*
10000 UI/settimana EPO alfa o beta o DARBO 50 microg /settimana*
8000 UI/settimana EPO alfa o beta o DARBO 4 microg /settimana*
6000 UI/settimana EPO alfa o beta o DARBO 30 microg /settimana*
4000 UI/settimana EPO alfa o beta o DARBO 20 microg /settimana*
Interruzione trattamento con EPO alfa o beta,DARBO o altro ESA
Randomizzazione: 18000 UI/settimana EPO alfa o beta o DARBO 90 microg /settimana*
Controllo mensile ed ulteriore conferma Hb < 9.5 g/dL
Hb 9.5-12.5 g/dL
22000 UI/settimana EPO alfa o beta o DARBO 110 microg /settimana*
28000 UI/settimana EPO alfa o beta o DARBO 140 microg /settimana*
35000 UI/settimana EPO alfa o beta o DARBO 175 microg /settimana*
Permane 35000 UI/settimana EPO alfa o beta o DARBO175 microg /settimana* (DOSE MASSIMA AMMESSA)
Randomizzazione 4000 UI/settimana EPO alfa o beta o DARBO 20 microg/settimana
3000 EPO alfa o beta o DARBO 15 microg /settimana *
2000 EPO alfa o beta, DARBO 10 microg /settimana *
Interruzione trattamento con EPO alfa o beta, DARBO o altro ESA
Randomizzazione: 4000 UI/settimana EPO alfa o beta o DARBO 20 microg /settimana*
5000 UI/settimana EPO alfa o beta o DARBO 25 microg /settimana*
6000 UI/settimana EPO alfa o beta o DARBO 30 microg /settimana
8000 UI/settimana di EPO alfa o beta o DARBO 40 microg/settimana*
Aumento della dose di EPO alfa o beta, DARBO o altro ESA fino al raggiungimento di Hb 9.5 g/dL
* o dose equivalente di qualsiasi altro ESA

APPENDICE 2

DEFINIZIONE DEGLI ESITI CLINICI

1. **Mortalità totale**: si intende il decesso del paziente avvenuto per qualsiasi causa.
2. **Infarto del miocardio fatale:**
   a) morte che si verifica entro 7 giorni dall'insorgenza di un infarto del miocardio validato/documentato, in cui non ci sia stata evidenza di un'altra causa di morte.
   b) Evidenza autoptica di infarto recente e assenza di evidenza di altre cause di mortalità
   c) Un infarto del miocardio fatale potrebbe essere identificato come causa di morte a seguito della presenza di criteri indicativi di infarto che tuttavia non rispecchiano la stretta definizione di infarto del miocardio.
   I criteri indicativi sono:
      - Variazioni anomale nell'elettrocardiogramma indicative di un evento acuto
      - Anomalie dei livelli di marker senza evoluzioni (decesso del paziente prima delle successive misurazioni)
      - Altre evidenti anomalie.
3. **Infarto non fatale:** presenza di almeno due dei seguenti criteri: a) dolore toracico tipico, edema polmonare, sincope o shock; b) sviluppo all'ECG di onda Q patologica e/o comparsa o scomparsa di un sopraslivellamento del tratto ST, seguito da inversione dell'onda T in due o più delle 12 derivazioni elettrocardiografiche standard; c) incremento dei livelli dei marker biochimici indicativi di infarto miocardico (CPK >2 volte il limite normale superiore, o troponine elevate). Le ischemie miocardiche silenti non verranno considerate come infarto del miocardio.
4. **Ictus fatale:** morte che si verifica entro 7 giorni dall'insorgenza di ictus. La diagnosi di morte in seguito a ictus potrebbe verificarsi in meno di 24 ore dalla comparsa dei sintomi (deficit neurologici).
5. **Ictus non fatale:** presenza di segni tipici di deficit neurologico focale o globale, ad inizio improvviso, di durata superiore a 24 ore e giudicati di genesi vascolare. Gli ictus saranno ulteriormente classificati in funzione della causa probabile (ischemica o emorragica), definita sulla base dei dati clinici e strumentali (TAC, RM).
6. **Sindrome coronarica acuta (ospedalizzazione per):** trattamento in ambito ospedaliero come conseguenza di uno o più episodi ischemici a riposo di durata superiore a 5 minuti e supportati da almeno uno dei seguenti criteri: a) comparsa di un sottoslivellamento del tratto ST >0.5 mm; b) comparsa di un sopraslivellamento del tratto ST >0.5 mm; c) comparsa di un sopraslivellamento persistente (>30 minuti) del tratto ST<0.5 mm; d) incremento dei livelli di CPK o CPK-MB oltre il limite normale superiore; e) incremento dei livelli di troponine T e I.
7. **Attacchi ischemici transitori (ospedalizzazione per):** disturbi acuti della funzione neurologica focale o monoculare (amaurosis fugax) con sintomi che durano meno di 24 ore e che si presumono essere dovuti ad un meccanismo vascolare
8. **Intervento di rivascolarizzazione coronarica non progammato (ospedalizzazione per):** trattamento in ambito ospedaliero consistente in intervento coronarico percutaneo (PTCI-Percutaneous Transluminal Coronary Intervention) (es. angioplastica, stenting, aterectomia, ablazione laser) o by-

pass aortico-coronarico (CABG-Coronary Artery Bypass Graft) entro 14 giorni da un nuovo episodio acuto o dal peggioramento di segni/sintomi di sindrome coronarica acuta.

9. **Interventi di rivascolarizzazione periferica (ospedalizzazione per):** tutti gli interventi di rivascolarizzazione periferica successivi a vasculopatia periferica.
10. **Trombosi dell'accesso vascolare:** Occlusione totale o parziale dell'accesso che lo renda non idoneo al trattamento di emodialisi e per la quale si renda necessario intervento chirurgico di revisione o ricostituzione di accesso
11. **Eventi ipertensivi:** crisi ipertensive con sintomi simili a quelli dell'encefalopatia e convulsioni tonico-cloniche che richiedono immediata assistenza medica (come riportato da foglietto illustrativo) e/o incrementi dei valori pressori per i quali il medico sperimentatore proceda alla somministrazione acuta di terapia farmacologica
12. **Convulsioni:** convulsioni tonico-cloniche

APPENDICE 3

TIMETABLE DELLO STUDIO

Questo elenco si riferisce alle indagini ed alle attività previste dal protocollo dello studio ma non si sostituisce all'elenco delle indagini svolte nell'ambito di una normale pratica clinica emodialitica.

Rilevazione Parametri	Cadenza visite					
	Visita randomizzazione	Visita 1 mese	Visita 2 mesi	Visita 3 mesi	Visita 6 mesi	Visita 12 mesi
Consenso	X					
Anamnesi del paziente	X					
Rilevazione livelli di Hb	X	X	X	X	X	X
Htc, MCV, MCH, MCHC, piastrinemia		X	X	X		
Emocromo completo	X				X	X
Valutazione profilo lipidico, glicemico, funzionalità renale ed epatica	X				X	X
Pressione arteriosa e frequenza cardiaca	X	X	X	X	X	X
Valutazione efficacia e sicurezza		X	X	X	X	X
Dettagli relativi alle terapie intradialitiche e/o domiciliari	X	X	X	X	X	X
Dettagli relativi alla dialisi (peso secco, incremento ponderale interdialitico, flusso del sangue in dialisi, nPCR, Kt/V,* durata e tipo dialisi, filtro)	X	X	X	X	X	X
Questionario sulla Qualità di Vita	X				X	X

**Nell'ambito della visita di randomizzazione dovranno essere raccolti i valori di Hb, peso secco, Kt/V relativi a 2 mesi e 1 mese precedenti la randomizzazione stessa.* (preceded by *)

E' pratica comune eseguire tutti i prelievi durante la seduta dialitica centrale di una delle settimane del mese e riportare i valori nella scheda raccolta dati relativa alla visita in corso in quel mese.

*# **RILEVAZIONE DEI LIVELLI DI EMOGLOBINA:** i livelli di Hb dovranno essere rilevati anche al tempo: 2 settimane, 6 settimane e 10 settimane dalla data di randomizzazione e registrati rispettivamente alla visita 1 mese, visita 2 mesi e visita 3 mesi. Qualora vi fosse un valore di Hb < 9.5 g/dL o > 12.5 g/dL, sarà necessario effettuare una seconda rilevazione e se tale valore fosse nuovamente riconfermato si dovrà procedere alla modifica del dosaggio di ESA seguendo l'algoritmo in appendice 1. Inoltre tutte le rilevazioni di Hb per le quali siano state necessarie modifiche del dosaggio di ESA andranno riportate nella scheda "Modifiche del dosaggio di ESA".*

APPENDICE 4

STANDARDS DI CURA

Nel corso dello studio si mirerà al raggiungimento delle seguenti misure di performance clinica con il ricorso agli interventi terapeutici utilizzati nella normale pratica clinica emodialitica:

- Kt/V (SP)≥ 1.3
- Albuminemia >35 g/L
- nPCR>1.0 g/kg/die
- Ferritinemia 200-500 μg/L
- TSAT 30-40%
- Calcemia 8.4-9.5 mg/dL
- Fosforemia 3.5-5.5 mg/dL
- Paratormone (PTH): 150-300 pg/mL
- Pressione arteriosa sistolica (predialisi) ≤140 mmHg
- Pressione arteriosa diastolica (predialisi) ≤90 mmHg
- Incremento ponderale interdialitico medio mensile ≤4% del peso secco
- Flusso sangue (Qb) in dialisi >300 mL/min
- Colesterolemia totale <175 mg/dl
- Colesterolemia LDL <100 mg/dl
- Colesterolemia HDL ≥40 mg/dl
- Trigliceridemia <180 mg/dl

APPENDICE 5

EVENTI AVVERSI ed END POINT

Per la valutazione della sicurezza dei trattamenti oggetto di questo studio, è previsto il monitoraggio e la registrazione di tutti gli eventi avversi e degli eventi avversi gravi.

La valutazione della sicurezza dei trattamenti in studio sarà effettuata da un Data Safety and Monitoring Committee, che procederà annualmente alla revisione dei dati dello studio.

Eventi avversi

Un evento avverso è definibile come un sintomo, segno o condizione clinica indesiderabile che si verifica dopo l'inizio del trattamento in studio, anche nel caso in cui l'evento non sia correlato al trattamento stesso.

Le condizioni cliniche e/o patologiche presenti prima dell'inizio del trattamento in studio, sono considerate eventi avversi solo se vi è un peggioramento dopo l'inizio del trattamento suddetto.

Gli eventi di tossicità correlati alla terapia con ESA devono essere considerati eventi avversi e per questo riportati sull'apposita scheda "Evento Avverso".

Gestione e notifica eventi avversi

Gli eventi avversi devono essere riportati se si verificano in qualsiasi momento dall'inizio del trattamento in studio e fino a 14 giorni dopo la fine del trattamento.

Le informazioni relative a tutti gli eventi avversi, compresi gli effetti collaterali dei farmaci in studio, dovranno essere registrate sulla relativa scheda di raccolta dati (scheda "Evento Avverso"), fornendo le seguenti informazioni: descrizione dell'evento, durata (data di inizio e di fine), grado di severità, correlazione con i farmaci in studio, azioni intraprese (trattamento, test diagnostici), esito.

Tutti gli eventi avversi devono essere trattati fino alla loro risoluzione.

Eventi avversi gravi

Un evento avverso grave è un segno o sintomo o condizione clinica non desiderata che risponde ai seguenti requisiti:

- risultino in un decesso;
- rappresentino un pericolo per la vita;
- richiedano l'ospedalizzazione del paziente o un prolungamento dell'ospedalizzazione in pazienti già ospedalizzati;
- determinino una disabilità persistente o significativa;
- rappresentino eventi di interesse medico importanti in base all'opinione del medico responsabile (ovvero qualsiasi evento che non sia immediatamente pericoloso per la vita e non risulti in un decesso o in ospedalizzazione ma che potrebbe arrecare nocumento al paziente o necessitare la somministrazione di interventi terapeutici per prevenire una qualsiasi delle condizioni precedentemente elencate).

Gestione e notifica eventi avversi

Durante tutta la durata dello studio, qualsiasi evento avverso grave dovrà essere comunicato tempestivamente (entro 24 ore) al Centro di Coordinamento, che provvederà ad inoltrare la notifica al Ministero della Salute". In caso di decesso, lo sperimentatore dovrà darne comunicazione al Centro di Coordinamento entro 24 ore e al Comitato Etico tempestivamente. Tutti i dettagli su qualsiasi evento avverso grave, anche se non correlabile con i trattamenti in studio, andranno inoltre riportati su un'apposita scheda (scheda "Evento Avverso Grave").

Nel caso in cui si verificasse un evento avverso serio che lo sperimentatore giudica in relazione ai farmaci in studio (agenti stimolanti l'eritropoiesi), in base alla normativa vigente in tema di farmacovigilanza (Decreto legislativo 8 Aprile 2003, n.95) deve essere compilata la "Scheda unica di segnalazione di sospetta reazione avversa", da inviare al responsabile di farmacovigilanza della struttura sanitaria di appartenenza.

Gestione e notifica degli end-point

In caso di insorgenza di uno degli eventi che rappresentano gli end-point dello studio, sarà necessario notificare l'evento/gli eventi al Quality Team e compilare l'apposita scheda (scheda "End-point"), che dovrà essere accompagnata da copia della documentazione clinica (schede di dimissione ospedaliera, certificati di morte, ecc., da cui dovrà essere eliminato ogni dettaglio relativo alle informazioni anagrafiche del paziente) che ne attesti la natura.

APPENDICE 6

STRUTTURA ORGANIZZATIVA E AREE DI RESPONSABILITÀ

Steering Committee

Lo Steering Committee è responsabile di quanto segue:

- il disegno e le modalità di conduzione dello studio C.E. DOSE
- la preparazione del protocollo, delle successive revisioni e valutazione delle versioni finali
- l'organizzazione delle riunioni dello Steering Committee
- la pubblicazione dei risultati dello studio
- la revisione dell'andamento dello studio e, laddove necessario, la valutazione di variazioni al protocollo e/o alle modalità operative
- la revisione della letteratura e la segnalazione di eventuali nuovi studi rilevanti per la conduzione dello studio

Data Safety and Monitoring Committee

Il Data Safety and Monitoring Committee, organo indipendente, è responsabile di:

- rivedere i dati ad interim derivanti dallo studio C.E. DOSE
- valutare sicurezza, efficacia, conformità con il protocollo e andamento dello studio

Centro di Coordinamento

I responsabili del Centro di Coordinamento curano quanto segue:

- aspetti contrattuali ed amministrazione del budget
- randomizzazione centralizzata
- assistenza ai centri, operazioni di Institutional Review ed aspetti etici
- organizzazione scientifica di riunioni con i partecipanti, ivi inclusi i medici di medicina generale
- il disegno, la valutazione e la cura dei database

Quality Team

I responsabili del Quality Team, organizzazione interna al Centro di Coordinamento, si occuperanno di quanto segue:

- aspetti legali e normativi
- monitoraggio
- aspetti informatici (il disegno, la valutazione e la cura dei database)
- organizzazione tecnica di riunioni con i partecipanti, ivi inclusi i medici di medicina generale
- preparazione delle documentazioni relative alle modalità operative
- gestione dei dati ed archiviazione dei documenti

Centri partecipanti

Lo studio sarà condotto da ambulatori di terapia dialitica.

Responsabilità dei Centri partecipanti sono le seguenti:

- fornire adeguati ambienti clinici per la valutazione e il follow-up dei pazienti
- accordarsi con i colleghi (es. medici di medicina generale) per le eventuali prescrizioni di farmaci e le dovute attenzioni da adoperare nei confronti dei pazienti randomizzati
- identificazione ed arruolamento dei pazienti
- organizzazione degli appuntamenti per le visite di follow-up
- tutte le procedure cliniche
- gestione delle problematiche riportate dai pazienti
- valutazione e reporting degli end-point, degli eventi avversi e delle reazioni avverse

APPENDICE 7

ATTIVITÀ DI MONITORAGGIO

La responsabilità della gestione dello studio e del monitoraggio della sicurezza dei soggetti in studio sarà garantita da alcuni comitati. Ciascun comitato sarà tenuto a dotarsi di procedure scritte (ad eccezione dello Steering Committee) e a redigere verbali per ogni riunione effettuata.

Steering Committee

Le funzioni più importanti dello Steering Committee comprendono: rivedere e approvare tutti gli emendamenti al protocollo e dei sottoprogetti; seguire l'andamento dello studio e trasmettere le informazioni agli sperimentatori coinvolti nello studio; intervenire in seguito alle raccomandazioni del Data and Safety Monitoring Committee e dell'End-point Committee; rivedere e approvare le pubblicazioni e le presentazioni.

Data and Safety Monitoring Committee (DSMC)

Il Data and Safety Monitoring Committee (DSMC) è un comitato indipendente i cui membri non possono partecipare direttamente alla conduzione dello studio ed hanno le seguenti responsabilità: salvaguardare il benessere dei pazienti arruolati nello studio; rivedere il protocollo e il piano dell'analisi ad interim; verificare a intervalli prestabiliti o quando richiesto dallo Steering Committee la sicurezza, l'efficacia, la conformità con il protocollo e l'andamento dello studio; produrre raccomandazioni per lo Steering Committee riguardo ogni problema esistente o potenziale. Sebbene il DSMC possa decidere autonomamente le procedure più idonee, nell'esame dei dati sull'efficacia e sulla sicurezza dovranno essere considerate le regole per l'interruzione dello studio specificate nel disegno del protocollo. Ogni raccomandazione maggiore, come ad esempio l'emissione di emendamenti al protocollo e l'interruzione dello studio, deve essere esaminata e ratificata dallo Steering Committee prima della sua applicazione.

End-point Committee

L'End-point Committee ha le seguenti responsabilità: trovare un accordo sulle definizioni e classificazioni standard per l'attribuzione degli end-point; fornire una valutazione indipendente ed in cieco degli end-point di efficacia, come definiti nel protocollo, basandosi su classificazioni e definizioni standardizzate. Le decisioni prese dall'End-point Committee devono essere considerate definitive e costituiranno la base di tutte le analisi finalizzate alla produzione di documenti per le autorità sanitarie o per pubblicazioni. L'End-point Committee dovrà procedere all'attribuzione degli end-point in base al "Manuale Codifica degli eventi" sviluppato in accordo con lo Steering Committee.

PROCEDURE DI MONITORAGGIO

Le procedure di monitoraggio verranno affidate al Quality Team della Fondazione Mario Negri Sud.

Prima dell'inizio dello studio, un monitor illustrerà il protocollo e la scheda raccolta dati agli sperimentatori ed al loro staff, in occasione di una visita di inizio studio o di un incontro con i clinici partecipanti. Durante lo studio i monitor visiteranno e/o contatteranno per via telefonica il centro regolarmente per verificare la completezza dei dati, l'accuratezza della compilazione delle schede di raccolta dati, l'aderenza al protocollo ed alle Good Clinical Practice (GCP) e l'andamento dell'arruolamento. I monitor devono essere sottoposti a regole di condotta analoghe al segreto professionale. Il referente clinico del centro partecipante allo studio dovrà essere disponibile ad assistere il monitor durante queste visite.

Lo sperimentatore dovrà fornire al monitor l'accesso ai dati clinici o a quelli dell'ospedalizzazione, per confermare la consistenza delle informazioni registrate nelle schede raccolta dati. Tutte le informazioni relative all'identità del soggetto dovranno rimanere presso il centro partecipante allo studio. Le procedure standard di monitoraggio prevedono una completa verifica del consenso informato, l'aderenza ai criteri di inclusione/esclusione, la documentazione degli eventi avversi seri e la registrazione delle variabili di sicurezza e di efficacia.

REGISTRAZIONE DEI DATI E ARCHIVIAZIONE DEI DOCUMENTI

Lo sperimentatore dovrà compilare le schede di raccolta dati e trasmettere le informazioni secondo le modalità stabilite all'inizio dello studio dal Quality Team; infine dovrà conservare una copia della scheda, insieme agli altri documenti dello studio (protocollo, emendamenti al protocollo, ecc.) nell'Investigator's File, che dovrà essere custodito in un posto sicuro. Le schede raccolta dati saranno compilate secondo quanto previsto dalle linee guida per la compilazione delle schede dello studio.

Le informazioni dei pazienti saranno riportate sulla scheda in modo che i soggetti siano identificati solamente da un numero. Se, per motivi eccezionali di sicurezza o per richiesta delle autorità, sorgesse la necessità di identificare un paziente, sia il Centro di Coordinamento sia lo sperimentatore saranno vincolati a trattare queste informazioni in maniera strettamente confidenziale.

Gli sperimentatori dovranno archiviare tutti i documenti originali dei pazienti in studio, le informazioni mediche e demografiche ed una copia del consenso informato firmato dal paziente. Tutte le informazioni riportate sulle schede raccolta dati dovranno essere rintracciabili nella documentazione clinica originale, contenuta nella cartella clinica del paziente.

I documenti essenziali (elencati di seguito) dovranno essere archiviati dagli sperimentatori per almeno 7 anni dopo la chiusura dello studio. Il Centro di Coordinamento notificherà agli sperimentatori e alle istituzioni quando non sarà più necessario conservare il materiale dello studio. Gli sperimentatori aderiranno alle procedure di archiviazione dei documenti firmando il protocollo. La documentazione essenziale include:

1. Approvazione del protocollo e di tutti gli emendamenti da parte del Comitato Etico Indipendente locale
2. Tutti i documenti originali
3. Le copie delle schede raccolta dati (Case Report Form, CRF)

4. Il consenso informato (con il numero e nome dello studio)
5. Qualsiasi altro documento pertinente allo studio

Protezione dei dati personali

Ad ogni paziente verrà presentata l'informativa al trattamento dei dati personali e prima di entrare nello studio dovrà firmare il consenso al trattamento dei dati personali.

Ogni informazione che riguardi il paziente, la cui raccolta ed elaborazione risulti connessa ed indispensabile al conseguimento degli obiettivi della presente valutazione preliminare, sarà trattata da tutto il personale implicato nella gestione dello studio, dalle autorità regolatorie deputate al controllo dei dati e dai medici che seguiranno il paziente con modalità idonee a garantirne l'assoluta riservatezza, confidenzialità e sicurezza in conformità a quanto previsto dalle norme per la tutela dei dati personali e del diritto alla riservatezza (legge 31 dicembre 1996 n. 675 e successive modificazioni/integrazioni). In particolare, ai sensi e per gli effetti dell'Articolo 10, 1° comma della Legge citata, sarà rispettato quanto segue:

Finalità e modalità di raccolta e trattamento dei dati

I dati raccolti saranno costituiti da: età, sesso, gruppo etnico di appartenenza e dati clinici altrimenti sensibili in quanto idonei a rivelare lo stato di salute. Detti dati, contenuti nella cartella ambulatoriale e nei referti degli esami di laboratorio citati nel presente documento, saranno trascritti dal medico responsabile dello studio su supporto cartaceo e/o informatico per le esclusive finalità connesse all'espletamento della valutazione preliminare.

Ambito di comunicazione e di diffusione

Per il controllo di qualità della ricerca è molto importante poter esaminare la cartella ambulatoriale ed i referti degli esami di laboratorio allo scopo di verificare la correttezza, la completezza e la congruità dei dati raccolti. Di conseguenza verrà chiesto al paziente di autorizzare il medico dell'ospedale a far esaminare - per tali scopi - la documentazione clinica originale al personale qualificato per il monitoraggio ed alle autorità regolatorie deputate al controllo dei dati.

Diritti dell'interessato ai sensi dell'Art. 7, D. Lgs. 196/03

In qualità di interessato al trattamento dei propri dati personali il paziente avrà pieno accesso per il tramite del medico dell'ospedale alle informazioni che lo riguardano con facoltà di esercitare in merito a tali informazioni tutti i diritti di cancellazione, trasformazione, integrazione, aggiornamento, rettifica e blocco entro i limiti previsti dall'art.7 D. Lgs. 196/03 in materia di tutela dei dati personali.

APPENDICE 8

DIVULGAZIONE DELLE INFORMAZIONI, ETICA E CONFIDENZIALITÀ

Firmando il protocollo, gli sperimentatori accettano di considerare le informazioni fornite dalla Fondazione Mario Negri Sud (FMNS) come strettamente confidenziali; la stessa confidenzialità sarà richiesta anche allo staff della FMNS stesso ed al Comitato Etico Indipendente Locale. I documenti dello studio, distribuiti dalla FMNS (protocollo, CRF ed altro materiale), dovranno essere conservati nel Trial Master File che dovrà essere custodito in modo appropriato in un luogo in grado di garantire la riservatezza. Le informazioni fornite dalla FMNS agli sperimentatori non potranno essere divulgate ad altri senza che la FMNS dia una autorizzazione scritta; fanno eccezione le informazioni contenute nel modulo informativo che sarà fornito ai pazienti che vogliono partecipare allo studio.

SOSPENSIONE DELLO STUDIO

La Fondazione Mario Negri Sud si riserva i diritti di sospendere lo studio in base alle condizioni specificate nel protocollo.

ETICA E GOOD CLINICAL PRACTICE

Questo studio verrà condotto in accordo con il protocollo, con le ICH-GCP e con le procedure operative standard della Fondazione Mario Negri Sud (SOP). Queste sono state stilate per assicurare l'aderenza alle GCP come descritto in:

1. ICH Harmonized Tripartite Guidelines for Good Clinical Practice 1996.
2. Dichiarazione di Helsinki, riguardante le ricerche mediche nell'uomo (Recommendations guiding Physicians in Biomedical Research involving Human Subjects, Helsinki 1964 emendamenti Tokyo 1975, Venezia 1983, Hong Kong 1989, Sommerset West 1996, Edimburg 2000).
3. DM 15 luglio 1997 n°162: Recepimento delle linee guida dell'Unione Europea di Buona Pratica Clinica per la esecuzione delle sperimentazioni cliniche dei medicinali.
4. DLgs 24 giugno 2003 n°211: Attuazione della direttiva 2001/20/CE relativa all'applicazione della buona pratica clinica nell'esecuzione delle sperimentazioni cliniche di medicinali per uso clinico.
5. DM 17 dicembre 2004: Prescrizioni e condizioni di carattere generale, relative all'esecuzione delle sperimentazioni cliniche dei medicinali, con particolare riferimento a quelle ai fini del miglioramento della pratica clinica, quale parte integrante dell'assistenza sanitaria.
6. DLgs 6 novembre 2007 n°200: Attuazione della direttiva 205/58/CE recante i principi e linee guida dettagliate per la buona pratica clinica relativa ai medicinali in fase di sperimentazione a uso umano, nonché requisiti per l'autorizzazione alla fabbricazione o importazione di tali medicinali.

Gli sperimentatori, firmando il protocollo, sottoscrivono l'aderenza alle istruzioni e procedure descritte in esso ed inoltre dichiarano la propria aderenza ai principi di GCP.

Comitato Etico Indipendente

Prima dell'inizio dello studio il protocollo, il modulo di consenso informato e le informazioni fornite ai pazienti dovranno essere visionati dal Comitato Etico Indipendente Locale. Un documento firmato e datato dell'avvenuta approvazione del modulo di consenso informato e del protocollo dovrà essere consegnato alla Fondazione Mario Negri Sud prima dell'inizio dello studio. Qualsiasi emendamento sostanziale al protocollo dovrà essere approvato da questo comitato. Qualsiasi emendamento non sostanziale verrà inviato per notifica a tale comitato.

Consenso informato

Tutti i partecipanti allo studio saranno informati, in maniera chiara e semplice, degli scopi dello studio e dei rischi in esso coinvolti. Verrà loro spiegato che possono rifiutare di partecipare allo studio o abbandonarlo in qualsiasi momento, senza che vi possa essere in futuro pregiudizio alcuno relativamente alla fornitura della migliore assistenza sanitaria necessaria nel loro interesse. Il consenso informato dovrà essere ottenuto da tutti i partecipanti. Detto consenso dovrà essere ottenuto verbalmente e per iscritto dopo che il partecipante avrà letto la relativa modulistica.

Qualsivoglia modifica relativa al consenso ed al modulo informativo per il paziente dovrà prevedere l'approvazione da parte del Comitato Etico Indipendente.

Lo sperimentatore dovrà illustrare ad ogni soggetto la natura dello studio, lo scopo, le procedure utilizzate, la durata, i potenziali rischi e benefici ed ogni disagio che esso potrà comportare. Ogni soggetto deve essere informato che la partecipazione allo studio è volontaria e che pertanto potrà abbandonarlo in qualsiasi momento, e che questa decisione non influenzerà in alcun modo la sua assistenza o i rapporti con il medico curante.

Il consenso informato dovrà essere scritto in un linguaggio comprensibile e non tecnico. Il soggetto, prima di apporre la propria firma e data, dovrà leggere e valutare attentamente il consenso, e, una volta firmato, dovrà conservarne una copia. Nessun paziente può entrare nello studio prima di fornire il proprio consenso.

La scheda di consenso informato è considerata parte del protocollo; entrambi dovranno essere sottoposti dagli sperimentatori al Comitato Etico Indipendente Locale per l'approvazione.

APPENDICE 9

DICHIARAZIONE DI HELSINKI

Gli sperimentatori dovranno condurre lo studio in accordo con la Dichiarazione di Helsinki. Una copia di tale dichiarazione sarà fornita insieme al materiale dello studio ad ogni singolo centro.

Associazione Medica Mondiale (AMM)
Dichiarazione di Helsinki

Principi etici per la ricerca medica che coinvolge soggetti umani

Adottata dalla 18ª Assemblea Generale dall'AMM a Helsinki, Finlandia, nel giugno 1964 ed emendata dalla 29ª Assemblea Generale a Tokyo, Giappone, nell'ottobre 1975, dalla 35ª Assemblea Generale a Venezia, Italia, nell'ottobre 1983, dalla 41ª Assemblea Generale a Hong Kong, nel settembre 1989, dalla 48ª Assemblea Generale a Somerset West, Repubblica del Sud Africa, nell'ottobre 1996 e dalla

52ª Assemblea Generale a Edimburgo, Scozia, nell'ottobre 2000.

A. Introduzione

1. L'Associazione Medica Mondiale ha elaborato la Dichiarazione di Helsinki come dichiarazione di principi etici che forniscano una guida per i medici e per gli altri partecipanti ad una ricerca medica che coinvolge soggetti umani. La ricerca medica che coinvolge soggetti umani include la ricerca su materiale umano identificabile o su altri dati identificabili.
2. È dovere del medico promuovere e salvaguardare la salute delle persone. Le sue conoscenze e la sua coscienza sono finalizzate al compimento di questo dovere.
3. La Dichiarazione di Ginevra dell'Associazione Medica Mondiale impegna il medico con le parole "La salute del mio paziente sarà la mia preoccupazione principale", e il Codice Internazionale di Etica Medica dichiara che "Un medico dovrà agire solo nell'interesse del paziente quando fornisca una cura medica che possa avere l'effetto di indebolire lo stato fisico e mentale del paziente".
4. Il progresso medico è fondato sulla ricerca la quale a sua volta si deve basare in qualche misura su una sperimentazione che coinvolga soggetti umani.
5. Nella ricerca su soggetti umani, le considerazioni correlate con il benessere del soggetto umano devono avere la precedenza sugli interessi della scienza e della società.
6. Lo scopo primario della ricerca medica che coinvolga soggetti umani è quello di migliorare le procedure preventive, diagnostiche e terapeutiche e di comprendere l'eziologia e la patogenesi della malattia. Anche i più comprovati metodi preventivi, diagnostici e terapeutici devono continuamente essere messi in discussione mediante la ricerca sulla loro efficacia, efficienza, accessibilità e qualità.
7. Nella pratica medica corrente e nella ricerca medica, la maggior parte delle procedure preventive, diagnostiche e terapeutiche implicano rischi ed aggravi.
8. La ricerca medica è sottoposta agli standard etici che promuovono il rispetto per tutti gli esseri umani e proteggono la loro salute e i loro diritti. Alcuni soggetti di ricerca sono vulnerabili e richiedono una speciale protezione. Devono essere riconosciuti le particolari necessità di coloro che sono economicamente e medicalmente svantaggiati. Una speciale attenzione è pure richiesta per coloro che non possono dare o che rifiutano il consenso personale, per coloro che possono essere esposti a dare il consenso sotto costrizione, per coloro che non beneficeranno personalmente dalla ricerca e per coloro per i quali la ricerca è associata alla cura.
9. I ricercatori devono essere al corrente dei requisiti etici, giuridici e regolatori della ricerca sui soggetti umani, sia i requisiti nazionali sia quelli internazionali, ove applicabili. Nessun requisito nazionale di natura etica, giuridica o regolatoria deve poter ridurre o eliminare alcuna delle protezioni per i soggetti umani esposte in questa Dichiarazione.

B. Principi basilari per tutta la ricerca medica

10. Nella ricerca medica è dovere del medico proteggere la vita, la salute, la riservatezza e la dignità del soggetto umano.
11. La ricerca medica che coinvolge soggetti umani deve essere conforme ai principi scientifici universalmente accettati e deve essere basata su una approfondita conoscenza della letteratura scientifica, di altre rilevanti fonti di informazione, e su un'adeguata sperimentazione in laboratorio e, ove appropriato, sull'animale.
12. Un'appropriata cautela deve essere posta nella conduzione di ricerche che possano incidere sull'ambiente, e deve essere rispettato il benessere degli animali utilizzati per la ricerca.
13. Il disegno e l'esecuzione di ogni procedura sperimentale che coinvolga soggetti umani devono essere chiaramente descritti in un protocollo di sperimentazione. Tale protocollo deve essere sottoposto ad esame, commenti, orientamenti e, dove previsto, all'approvazione da parte di un comitato etico di revisione appositamente istituito, che deve essere indipendente dal ricercatore, dallo sponsor e da qualsiasi altro tipo di indebita influenza. Questo comitato indipendente deve essere conforme alle leggi ed ai regolamenti della nazione in cui la sperimentazione è condotta. Il comitato ha titolo per monitorare i trial in corso. Il ricercatore ha l'obbligo di fornire le informazioni di monitoraggio al comitato, specialmente quelle relative agli eventi avversi seri. Il ricercatore deve anche sottoporre al comitato, per la revisione, le informazioni relative a finanziamento, sponsor, appartenenze a istituzione, altri potenziali conflitti di interesse e incentivi per i soggetti di sperimentazione.
14. Il protocollo di ricerca deve sempre contenere una esposizione delle considerazioni etiche implicate e deve recare l'indicazione di conformità con i principi enunciati nella presente Dichiarazione.
15. La ricerca biomedica che coinvolge soggetti umani deve essere condotta solo da persone scientificamente qualificate e sotto la supervisione di un medico competente sul piano clinico. La responsabilità nei confronti del soggetto umano deve sempre ricadere sul personale medico qualificato e mai sul soggetto della ricerca, anche se questi ha dato il proprio consenso.
16. Ogni progetto di ricerca medica che coinvolga soggetti umani deve essere preceduto da un'attenta valutazione dei rischi e degli aggravi prevedibili in rapporto ai benefici attesi per il soggetto stesso o per altri. Ciò non preclude la partecipazione di volontari sani ad una ricerca medica. Il disegno di tutti gli studi deve essere pubblicamente disponibile.
17. I medici devono astenersi dall'intraprendere progetti di ricerca che coinvolgano soggetti umani a meno che non siano sicuri che i rischi implicati siano stati adeguatamente valutati e possano essere controllati in modo soddisfacente. I medici devono interrompere ogni ricerca se i rischi si presentano superiori ai potenziali benefici o se si è raggiunta già una prova definitiva di risultati positivi e benefici.
18. La ricerca medica che coinvolga soggetti umani deve essere condotta solo se l'importanza dell'obiettivo prevalga sui i rischi e gli aggravi connessi per il soggetto. Ciò è particolarmente importante quando i soggetti umani siano volontari sani.
19. La ricerca medica è giustificata solo se vi è una ragionevole probabilità che le popolazioni in cui la ricerca è condotta possano beneficiare dei risultati della ricerca.
20. I soggetti devono essere volontari e partecipare informati al progetto di ricerca.
21. Il diritto dei soggetti di sperimentazione alla salvaguardia della loro integrità deve essere sempre rispettato. Deve essere adottata ogni precauzione per rispettare la privacy del soggetto, la riservatezza sulle informazioni relative al paziente e per minimizzare l'impatto dello studio sulla integrità fisica e mentale del soggetto e sulla sua personalità.
22. In ogni ricerca su esseri umani ciascun potenziale soggetto deve essere adeguatamente informato degli scopi, dei metodi, delle fonti di finanziamento, di ogni possibile conflitto di interessi, della appartenenza istituzionale del ricercatore, dei benefici previsti e dei rischi potenziali connessi allo studio, nonché dei fastidi che esso potrebbe comportare. Il soggetto deve essere informato del diritto di astenersi dal partecipare allo studio o della possibilità di ritirare il consenso alla partecipazione in qualsiasi momento senza ritorsioni. Solo dopo essersi assicurato che il soggetto abbia compreso le informazioni, il medico deve ottenere dal soggetto il consenso informato, liberamente espresso, preferibilmente in forma scritta. Se il consenso non può essere ottenuto per iscritto, deve essere formalmente documentato e testimoniato un consenso non scritto.
23. Nell'ottenere il consenso informato al progetto di ricerca, il medico deve essere particolarmente attento quando il soggetto si trovi in una condizione di dipendenza nei suoi confronti o possa sentirsi costretto a dare il consenso. In questo caso il consenso informato deve essere ottenuto da un altro medico che conosca bene la ricerca ma non sia coinvolto in essa e che sia completamente indipendente nella relazione col soggetto.

24. Per un soggetto di ricerca che sia legalmente, fisicamente o mentalmente incapace di dare il consenso, o per un minore legalmente incapace, il ricercatore deve ottenere il consenso informato dal tutore legale, in accordo con la legislazione specifica. Questi gruppi di soggetti non devono essere inclusi in una ricerca a meno che la ricerca stessa non sia necessaria per promuovere la salute della popolazione rappresentata e tale ricerca non possa essere invece attuata su persone legalmente capaci.
25. Quando un soggetto giudicato legalmente incapace, come un minore, sia capace di dare un assenso alla decisione di partecipare in una ricerca, lo sperimentatore deve ottenere tale assenso in aggiunta a quello del tutore legale.
26. La ricerca su individui dai quali non sia possibile ottenere un consenso, incluso quello rappresentato o anticipato, deve essere attuata solo se la condizione fisica o mentale che impedisce di ottenere il consenso è una caratteristica necessaria della popolazione in studio. Le ragioni specifiche per coinvolgere soggetti di ricerca che si trovino in condizioni tali da renderli incapaci di dare un consenso informato devono essere dichiarate nel protocollo di sperimentazione per l’esame e l’approvazione da parte del comitato di revisione. Il protocollo deve dichiarare che il consenso a rimanere nella ricerca sarà ottenuto non appena possibile da parte dello stesso soggetto o da un rappresentante legalmente autorizzato.
27. Sia gli autori sia gli editori hanno obbligazioni etiche. Nella pubblicazione dei risultati della ricerca gli sperimentatori sono obbligati a salvaguardare l’accuratezza dei risultati. Sia i risultati negativi sia quelli positivi devono essere pubblicati o resi in qualche modo pubblicamente disponibili. Le fonti del finanziamento, l’appartenenza istituzionale ed ogni possibile conflitto di interessi devono essere dichiarati nella pubblicazione. Relazioni di sperimentazioni non conformi con i principi fissati in questa Dichiarazione non devono essere accettati per la pubblicazione.

C. Principi aggiuntivi per la ricerca medica associata alle cure mediche

28. Il medico può associare la ricerca medica con le cure mediche solo con il limite che la ricerca sia giustificata da un potenziale valore preventivo, diagnostico o terapeutico. Quando la ricerca medica è associata con le cure mediche si applicano degli standard addizionali per proteggere i pazienti che sono soggetti di ricerca.
29. I benefici, i rischi, gli aggravi e l’efficacia di un nuovo metodo devono essere valutati in confronto con quelli dei migliori metodi preventivi, diagnostici e terapeutici attualmente in uso. Ciò non esclude l’impiego di placebo, o l’assenza di trattamento, negli studi dove non esistono metodi comprovati di prevenzione, diagnosi o terapia.
30. A conclusione dello studio, ad ogni paziente entrato nello studio deve essere assicurato l’accesso ai migliori metodi preventivi, diagnostici e terapeutici di comprovata efficacia identificati dallo studio.
31. Il medico deve informare pienamente il paziente di quali aspetti della cura sono correlati con la ricerca. Il rifiuto di un paziente a partecipare in uno studio non deve mai interferire con la relazione medico-paziente.
32. Nel trattamento di un paziente, laddove non esistano comprovati metodi preventivi, diagnostici e terapeutici o questi siano stati inefficaci, il medico, con il consenso informato del paziente, deve essere libero di usare mezzi preventivi, diagnostici e terapeutici non provati o nuovi, se a giudizio del medico essi offrono speranza di salvare la vita, ristabilire la salute o alleviare la sofferenza. Laddove possibile, tali mezzi dovrebbero essere fatti oggetto di una ricerca disegnata per valutare la loro sicurezza ed efficacia. In tutti i casi, le nuove informazioni devono essere registrate e, dove opportuno, pubblicate. Tutte le altre linee-guida di questa Dichiarazione devono essere seguite.
