# Supplementary material for: Low versus high dose erythropoiesis-stimulating agents in hemodialysis patients with anemia: A randomized clinical trial
Source: PLoS One. 2017 Mar 1;12(3):e0172735. doi: 10.1371/journal.pone.0172735 (PMC5332066; doi:10.1371/journal.pone.0172735)
Supplement: S1 Table — Data are mean (standard error of mean), median (interquartile range), or number (proportion). Hemoglobin level stabilization was defined as two consecutive measurements between 10 and 12 g/dL. (DOCX) [file pone.0172735.s010.docx]

## **S1 Table. Intervention practice characteristics.**

| **Indicator** | **Low dose** | **High dose** | **p value** |
| --- | --- | --- | --- |
| Average variation of the allocated ESA at the end of treatment, mean difference (SEM) | 5158 (563) | -7285 (561) | **<0.001** |
| Average variation of the allocated ESA at the end of treatment, adjusted by body max index, mean difference (SEM) | 4776 (483) | -7352 (484) | **<0.001** |
| Number of patients with stable hemoglobin levels without need for >50% change in the allocated dose of ESA | 191 (62) | 186 (60) | 0.64 |
| Time from randomization to the first ESA dose variation (days) | 61 (32-110) | 54 (31-92) | 0.17 |
| Time from randomization to hemoglobin stabilization (days) | 34 (28-87) | 34 (28-92) | 0.41 |

Data are mean (standard error of mean), median (interquartile range), or number (proportion). Hemoglobin level stabilization was defined as two consecutive measurements between 10 and 12 g/dL.
