# Supplementary material for: Low versus high dose erythropoiesis-stimulating agents in hemodialysis patients with anemia: A randomized clinical trial
Source: PLoS One. 2017 Mar 1;12(3):e0172735. doi: 10.1371/journal.pone.0172735 (PMC5332066; doi:10.1371/journal.pone.0172735)
Supplement: S2 Table — (DOCX) [file pone.0172735.s011.docx]

## Table S2. Adverse events.

| **Adverse Events** | **Low**  **dose** | **High**  **dose** |
| --- | --- | --- |
| Blood and lymphatic system disorders | 7 | 1 |
| Cardiac disorders | 21 | 22 |
| Congenital, familial and genetic disorders | 1 | 1 |
| Endocrine disorders | 1 | 2 |
| Eye disorders | 1 | 0 |
| Gastrointestinal disorders | 11 | 22 |
| General disorders and administration site conditions | 21 | 12 |
| Hepatobiliary disorders | 6 | 3 |
| Infections and infestations | 47 | 53 |
| Injury, poisoning and procedural complications | 13 | 18 |
| Investigations | 2 | 1 |
| Metabolism and nutrition disorders | 9 | 8 |
| Musculoskeletal and connective tissue disorders | 5 | 7 |
| Neoplasms benign, malignant and unspecified | 9 | 9 |
| Nervous system disorders | 8 | 13 |
| Psychiatric disorders | 0 | 3 |
| Renal and urinary disorders | 9 | 0 |
| Reproductive system and breast disorders | 2 | 1 |
| Respiratory, thoracic and mediastinal disorders | 13 | 10 |
| Skin and subcutaneous tissue disorders | 7 | 3 |
| Surgical and medical procedures | 12 | 6 |
| Vascular disorders | 14 | 24 |
