# Supplementary material for: Low versus high dose erythropoiesis-stimulating agents in hemodialysis patients with anemia: A randomized clinical trial
Source: PLoS One. 2017 Mar 1;12(3):e0172735. doi: 10.1371/journal.pone.0172735 (PMC5332066; doi:10.1371/journal.pone.0172735)
Supplement: S3 Table — (DOCX) [file pone.0172735.s012.docx]

## **Table S3. Serious Adverse Events.**

| **Serious Adverse Events** | **Low dose** | **High dose** |
| --- | --- | --- |
| Blood and lymphatic system disorders | 4 | 2 |
| Cardiac disorders | 18 | 22 |
| Congenital, familial and genetic disorders | 0 | 1 |
| Gastrointestinal disorders | 4 | 7 |
| General disorders and administration site conditions | 16 | 6 |
| Hepatobiliary disorders | 3 | 2 |
| Infections and infestations | 25 | 33 |
| Injury, poisoning and procedural complications | 3 | 7 |
| Investigations | 2 | 0 |
| Metabolism and nutrition disorders | 3 | 7 |
| Musculoskeletal and connective tissue disorders | 0 | 2 |
| Neoplasms benign, malignant and unspecified | 5 | 6 |
| Nervous system disorders | 2 | 6 |
| Renal and urinary disorders | 2 | 0 |
| Respiratory, thoracic and mediastinal disorders | 7 | 9 |
| Skin and subcutaneous tissue disorders | 0 | 2 |
| Surgical and medical procedures | 9 | 8 |
| Vascular disorders | 7 | 13 |
