# Supplementary material for: Low versus high dose erythropoiesis-stimulating agents in hemodialysis patients with anemia: A randomized clinical trial
Source: PLoS One. 2017 Mar 1;12(3):e0172735. doi: 10.1371/journal.pone.0172735 (PMC5332066; doi:10.1371/journal.pone.0172735)
Supplement: S4 Table — a Data are unadjusted means (and standard error). b Data are unadjusted means (difference between groups from baseline to 12 months; delta of delta) and 95% confidence interval. The p value indicates the difference between low dose and high dose treatment over time (delta of delta [DD]). (DOCX) [file pone.0172735.s013.docx]

## S4 Table. Blood pressure end points.

|  | **Systolic blood pressure** | | | | **Diastolic blood pressure** | | | |
| --- | --- | --- | --- | --- | --- | --- | --- | --- |
| **Time point** | **Low dose group**  **(N=308) ^a^** | **High dose group**  **(N=318) ^a^** | **Difference between low dose and high dose over time (12 months versus baseline; 95% confidence interval) ^b^** | **p value** | **Low dose group**  **(N=307) ^a^** | **High dose group**  **(N=318) ^a^** | **Difference between high dose and low dose over time (12 months versus baseline; 95% confidence interval) ^b^** | **p value** |
| Baseline | 134.0 (22.0) | 133.1 (22.0) | Reference | -- | 70.6 (13.4) | 70.4 (13.5) | Reference | -- |
| 1 month | 134.4 (22.1) | 135.5 (22.4) | 2.0 (-1.3 to 5.3) | 0.24 | 71.0 (13.6) | 71.0 (13.7) | 0.1 (-1.8 to 2.0) | 0.89 |
| 2 months | 133.7 (22.4) | 134.0 (22.7) | 1.3 (-3.0 to 5.5) | 0.55 | 70.3 (13.7) | 71.2 (13.9) | 1.0 (-1.4 to 3.5) | 0.40 |
| 3 months | 132.1 (22.6) | 134.5 (23.0) | 3.2 (-1.5 to 8.0) | 0.18 | 69.4 (13.8) | 73.7 (14.4) | 2.1 (-0.7 to 4.8) | 0.14 |
| 6 months | 134.5 (25.3) | 135.2 (26.0) | 1.5 (-3.6 to 6.6) | 0.56 | 72.8 (14.1) | 71.6 (15.3) | 1.0 (-1.9 to 3.9) | 0.50 |
| 12 months | 132.6 (26.4) | 131.8 (27.2) | 0.1 (-5.3 to 5.5) | 0.97 | 71.4 (14.7) | 70.6 (13.4) | 0.4 (-2.7 to 3.4) | 0.82 |

^a^ Data are unadjusted means (and standard error). ^b^ Data are unadjusted means (difference between groups from baseline to 12 months; delta of delta) and 95% confidence interval. The p value indicates the difference between low dose and high dose treatment over time (delta of delta [DD]).
