# Supplementary material for: Low versus high dose erythropoiesis-stimulating agents in hemodialysis patients with anemia: A randomized clinical trial
Source: PLoS One. 2017 Mar 1;12(3):e0172735. doi: 10.1371/journal.pone.0172735 (PMC5332066; doi:10.1371/journal.pone.0172735)
Supplement: S5 Table — Data are expressed as frequency and percent. (DOCX) [file pone.0172735.s014.docx]

## S5 Table. Subgroup analyses for composite endpoint of death from any cause, nonfatal myocardial infarction, nonfatal stroke, or hospitalization for cardiovascular cause.

|  | **Low dose group**  **(N=324)** | **High dose group**  **(N=332)** | **Hazard ratio**  **(95% CI)** | **p value** |
| --- | --- | --- | --- | --- |
| **Age** |  |  |  |  |
| <60 years | 8 (9%) | 8 (9%) | 0.95 (0.33-2.78) | 0.92 |
| ≥60 years | 46 (21%) | 52 (22%) | 0.97 (0.65-1.45) | 0.90 |
| **Gender** |  |  |  |  |
| Men | 35 (18%) | 38 (20%) | 0.93 (0.58-1.49) | 0.20 |
| Women | 19 (17%) | 21 (16%) | 1.01 (0.53-1.89) | 0.74 |
| **Diabetes** |  |  |  |  |
| History of diabetes | 21 (30%) | 29 (32%) | 1.03 (0.58-1.82) | 0.90 |
| No history of diabetes | 33 (13%) | 31 (13%) | 1.02 (0.62-1.69) | 0.94 |
| **Cardiovascular disease** |  |  |  |  |
| History of myocardial infarction | 16 (27%) | 24 (30%) | 0.81 (0.43-1.52) | 0.50 |
| No history of myocardial infarction | 38 (14%) | 36 (14%) | 1.05 (0.66-1.69) | 0.82 |

Data are expressed as frequency and percent.
